# A Semi-Quantitative, Synteny-Based Method to Improve Functional Predictions for Hypothetical and Poorly Annotated Bacterial and Archaeal Genes

**DOI:** 10.1371/journal.pcbi.1002230

**Published:** 2011-10-20

**Authors:** Alexis P. Yelton, Brian C. Thomas, Sheri L. Simmons, Paul Wilmes, Adam Zemla, Michael P. Thelen, Nicholas Justice, Jillian F. Banfield

**Affiliations:** 1 Department of Environmental Science, Policy, and Management, University of California, Berkeley, California, United States of America; 2 Department of Earth and Planetary Sciences, University of California, Berkeley, California, United States of America; 3 Physical and Life Sciences Directorate, Lawrence Livermore National Laboratory, Livermore, California, United States of America; 4 Department of Plant and Microbial Biology, University of California, Berkeley, California, United States of America; University of California Davis, United States of America

## Abstract

During microbial evolution, genome rearrangement increases with increasing sequence divergence. If the relationship between synteny and sequence divergence can be modeled, gene clusters in genomes of distantly related organisms exhibiting anomalous synteny can be identified and used to infer functional conservation. We applied the phylogenetic pairwise comparison method to establish and model a strong correlation between synteny and sequence divergence in all 634 available Archaeal and Bacterial genomes from the NCBI database and four newly assembled genomes of uncultivated Archaea from an acid mine drainage (AMD) community. In parallel, we established and modeled the trend between synteny and functional relatedness in the 118 genomes available in the STRING database. By combining these models, we developed a gene functional annotation method that weights evolutionary distance to estimate the probability of functional associations of syntenous proteins between genome pairs. The method was applied to the hypothetical proteins and poorly annotated genes in newly assembled acid mine drainage Archaeal genomes to add or improve gene annotations. This is the first method to assign possible functions to poorly annotated genes through quantification of the probability of gene functional relationships based on synteny at a significant evolutionary distance, and has the potential for broad application.

## Author Summary

Based on trends between gene sequence divergence and gene order divergence over time, we developed a new synteny-based method to refine functional annotation. This method uses these trends to determine the probability that any two syntenous genes (genes that are sequential in two organisms) are functionally related. Organisms that are distant relatives have few syntenous genes, but these syntenous genes have a very high probability of functional relatedness. We applied this method to newly assembled genomes of co-occurring, uncultivated acid mine drainage Archaea in order to improve their gene annotations. This application revealed important physiological differences between the co-occurring organisms in this clade, including the ability of some but not all of the Archaea to manufacture vitamin B12 and to carry out anaerobic energy metabolism. We also used this method to identify new genes possibly involved in vitamin B12 synthesis, ether lipid synthesis, molybdopterin synthesis and utilization, and microbial immunity through the CRISPR system.

## Introduction

Gene function prediction is currently one of the fundamental problems in microbiology [1]. The improvement in DNA sequencing technologies has allowed for the sequencing of hundreds of full Bacterial and Archaeal genomes. However, in the dataset of full Bacterial and Archaeal genomes from NCBI, 874,583 genes out of 2,668,809 (∼33%) are annotated as hypothetical proteins, and 25% of the protein families in the PFAM database have unknown functions [1]. In addition to these un-annotated genes, many of the genes in these databases only have general function predictions or may have incorrect function predictions. Thus, improved protein functional prediction methods are urgently needed.

It has been proposed that correlations between synteny and evolutionary distance, in concert with homology, can be used for predicting protein function [2], [3], [4], [5]. Synteny has been used to predict the functional interaction of proteins, where interaction is defined as direct physical interaction, the regulation of one protein by the other, membership in a protein complex, or the sharing of a metabolic (or non-metabolic) pathway [4], [5], [6]. Various protein function prediction methods make use of synteny, as reviewed by Rogozin *et al.* in 2004 [3], [7], [8], [9], [10], [11], but do not consider evolutionary distance between genomes in their predictions. Preservation of synteny over large evolutionary distances should be weighted strongly in gene function prediction because it is likely the result of selection against rearrangements. Huynen and Snel noted the importance of finding the evolutionary distance at which gene order conservation becomes significant [12]. Snel *et al.* simulated random genome shuffling to determine the probability of conserved gene order in a specific number of genomes [13], and Von Mering *et al.* assessed the likelihood of protein relatedness based on the number of times gene order is conserved in the STRING database of genomes [4]. Here, we link the probability of syntenous protein relatedness and evolutionary distance so that we can determine which genomes are distant enough to accurately utilize synteny-based gene annotation. An overview of our method is provided in Figure S1. Our analyses included genomes of coexisting Archaea reconstructed from metagenomic sequence from biofilms growing in an extreme acid mine drainage (AMD) environment as well as published genomes. The inclusion of AMD Archaea allowed us to apply the method to newly assembled genomes from uncultivated organisms, and to show the utility of the method for comparative genomics and for improving annotations of proteins of unknown function.

## Results

### Genome rearrangement and genome reconstruction

**Figure 1:**
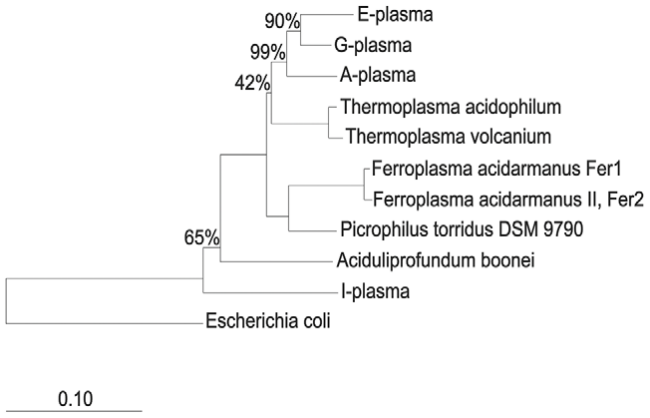
16S rRNA gene tree of AMD *Thermoplasmatales* Archaea. Bootstrapping values are indicated as percentages out of 1000 random samples.

We reconstructed four new genomes of uncultivated Archaea: A-, E-, G-, and I-plasma (Archaea; Euryarchaeota; Thermoplasmata; Thermoplasmatales). *Ferroplasma acidarmanus* (Ferroplasma Type I, Fer1) and *Ferroplasma* Type II (Fer2) have previously been described [14], [15], [16]. Only Fer1 has been isolated [14]. The phylogenetic placement of these organisms based on 16S rRNA gene sequences is shown in Figure 1. E- and G-plasma are most closely related, whereas I-plasma is distantly related and may not actually belong to the *Thermoplasmatales* lineage. Data describing the manually curated and binned composite genomes of these Archaea are listed in Table 1. Note that the estimates of the sizes of all genomes are similar. We used standard measures to evaluate genome completeness: a full suite of tRNAs, rRNAs, and orthologous marker genes in all genomes [17]. All of the genomes of the AMD *Thermoplasmatales* organisms except for A-plasma are near complete, according to our analysis (Table S1).

**Table 1:** Genome information for AMD *Thermoplasmatales* Archaea.

Genome	Length (Mb)	Coverage	GC content (%)	Number of tRNAs	rRNAs	Completeness
A-plasma	1.94	8×	46	48	16S, 23S, 5S	71.4%
E-plasma	1.66	9×	38	41	16S, 23S, 5S	100.0%
G-plasma	1.84	8×	38	44	16S, 23S, 5S	94.3%
I-plasma	1.69	20×	44	46	16S, 23S, 5S	100.0%
F-plasma 1	1.46	4.5×	36	44	16S, 23S, 5S	NA
F-plasma 2	1.82	10×	37	63*	16S, 23S, 5S	100.0%
F-plasma 1 isolate	1.94	13×	38	44	16S, 23S, 5S	100.0%

Genome completeness was estimated based on number of tRNAs, rRNAs, and orthologous marker genes.

*Reflects tRNAs in several closely related Fer2 strains sampled independently.

### Evolution

**Figure 2:**
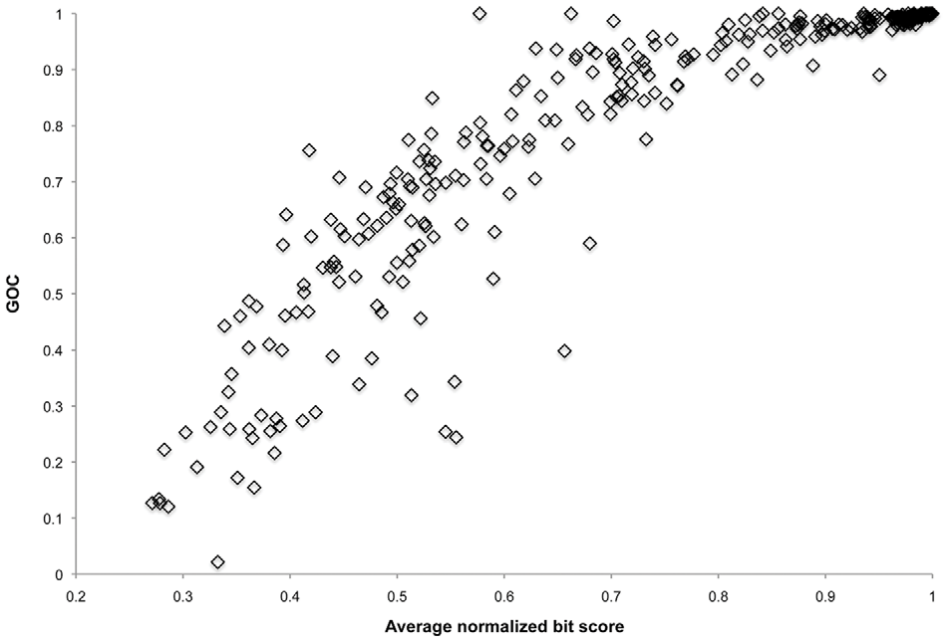
Synteny (gene order conservation) versus sequence divergence (average normalized bit score) in pairwise comparisons of 638 Bacterial and Archaeal genomes. The phylogenetic pairwise comparison method was used to carry out 319 comparisons. See Text S1 for details.

In order to carry out regression analysis on genome rearrangements and evolutionary distance, we used gene order conservation (GOC) as a measure of whole genome rearrangement. This metric is described by Rocha [18]. Figure 2 shows the relationship between GOC and evolutionary distance as measured by average normalized BLASTP bit score, a proxy for evolutionary distance.

Figure 2 includes results for genomes reconstructed for uncultivated AMD Archaea from metagenomic data. These genomes are incomplete (Table 1), and remaining gaps may affect our analyses. Thus, we investigated the effect of a limited amount of fragmentation on trends by shearing the genome of the *Ferroplasma acidarmanus* (Fer1) isolate into fragments that corresponded to the lengths of the fragments from our environmental datasets. The fragmented Fer1 pairwise comparisons followed the trend defined by all other genomes with a slight downward shift (Figure S2).

### Functional prediction of hypothetical and poorly annotated genes using synteny

Based on the clear relationship between evolutionary distance and synteny, we explored an improved neighborhood approach to protein functional prediction. We developed a method that involves an evolutionary distance-weighting for each pairwise comparison and incorporates the high probability of synteny due to chance in closely related organisms. We assumed that genes that remain syntenous in organisms separated by large evolutionary distances do so because of selective pressure to maintain function. Genes in predicted operons have previously been shown to rearrange at a slower rate than genes that are never found in operons [18]. We quantified the statistical significance of the difference between the populations for operon and non-operon genes using the phylogenetic pairwise comparison method [19] and the Wilcoxon signed-rank test. We used the phylogenetic pairwise comparison method to choose independent pairs of genomes for comparison and the Wilcoxon signed-rank test to test the hypothesis that there is a significant difference between the values of GOC for populations of pairwise comparisons that include operon genes versus those that include genes not in operons in the two genomes that were compared. The Wilcoxon test indicated a significant difference with a p-value of 1.017×10^−13^. We posit that this difference is due to stronger selection against the rearrangement of genes in operons because of co-regulation and functional linkage. As an approximation, we also assumed that genes that are not in operons and retain synteny do so solely by chance, that is, selection against rearrangements on non-operon genes is negligible for the purposes of our analysis.

**Figure 3:**
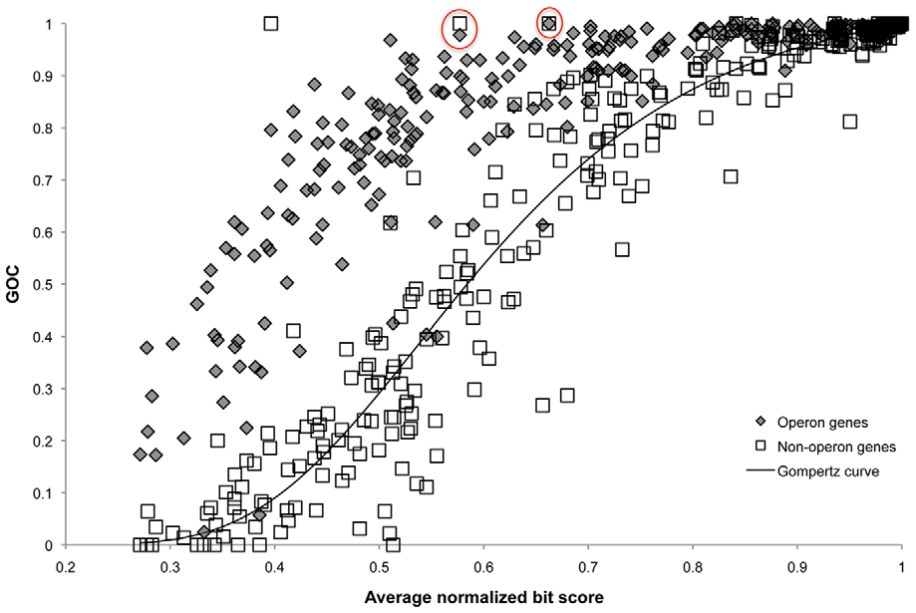
Synteny (gene order conservation) versus sequence divergence (average normalized bit score) in pairwise comparisons of 638 Bacterial and Archaeal genomes. Orthologs that are sometimes in predicted operons (operon genes) are compared separately from those that are never in operons (non-operon genes). The circled outliers come from comparisons of endosymbiont genomes, which have very small genomes and greater than expected conserved gene order in non-operon genes.

**Figure 4:**
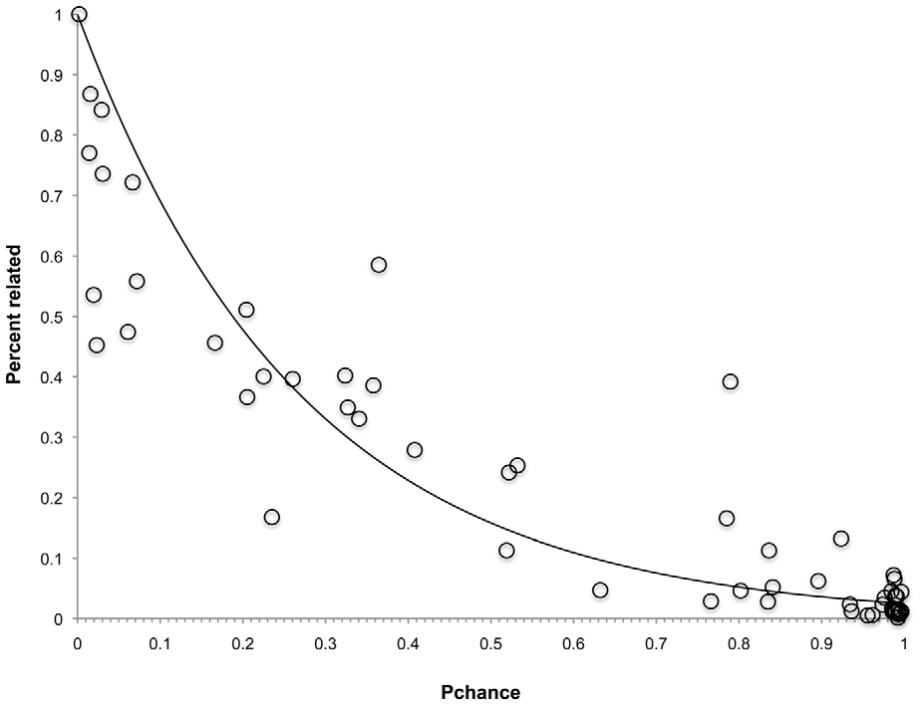
P_chance_ versus percent of syntenous genes that are related. P_chance_ is estimated from average normalized bit score, based on the model of GOC versus average normalized bit score. The percentage of syntenous genes that are related is based on data from the STRING protein database.

We used the trend between gene order conservation (GOC) and gene sequence divergence in genes not found in operons between the two genomes being compared (non-operon genes) to determine the degree of evolutionary divergence necessary to ensure that genes that retain synteny do *not* do so by chance. Because a GOC value approximates the probability of that any two genes retain synteny in a pairwise comparison at a given evolutionary distance, to estimate the probability that genes retained synteny due *solely to chance*, P_GOCn_, we modeled the relationship between the gene order conservation of non-operon genes (GOC_n_) and evolutionary distance (Figure 3). We calculated a measure of goodness of fit with the sum of squared errors (SSE) and the total sum of squares (TSS); 1-SSE/TSS = 0.9282. P_GOCn_ values were then compared to the percentage of syntenous genes that were functionally related in genomes included in the STRING database. We modeled this relationship as well, and interpreted the response variable as the probability that any two syntenous genes are functionally related, P_r_, (1-SSE/TSS = 0.7648, Figure 4). Both models were chosen from a set of models, using Akaike's information criterion (Table 2). We combined the models to predict P_r_ from measurements of evolutionary distance (Figure S1). Thus, for pairwise comparisons below a certain evolutionary distance threshold (a bit score value of 0.3129), P_r_ was statistically significant; syntenous genes have a 95% or greater probability of being functionally related (P_r_>0.95). A gene of unknown function in one such comparison is likely functionally related to its syntenous orthologs. In these cases, functional information for syntenous orthologs that would otherwise be disregarded due to low sequence similarity was used to improve annotations of genes of unknown function. Alternatively, if functional information was available for neighboring genes in a block for which synteny was preserved, the gene of unknown function was annotated as related to its neighbor.

**Table 2:** AIC comparison of regression models for NCBI and STRING data.

X variable	Y variable	Model type	AIC
Average normalized bit score	GOC	Linear	−455.4074
Average normalized bit score	GOC	Logarithmic	−493.2857
Average normalized bit score	GOC	Cubic	−573.4456
Average normalized bit score	GOC	Gompertz	−585.8681
GOC	Percent of syntenous genes that are related	Exponential	−69.750892
GOC	Percent of syntenous genes that are related	Linear	−7.646633
GOC	Percent of syntenous genes that are related	Quadratic	−51.991032

**Figure 5:**
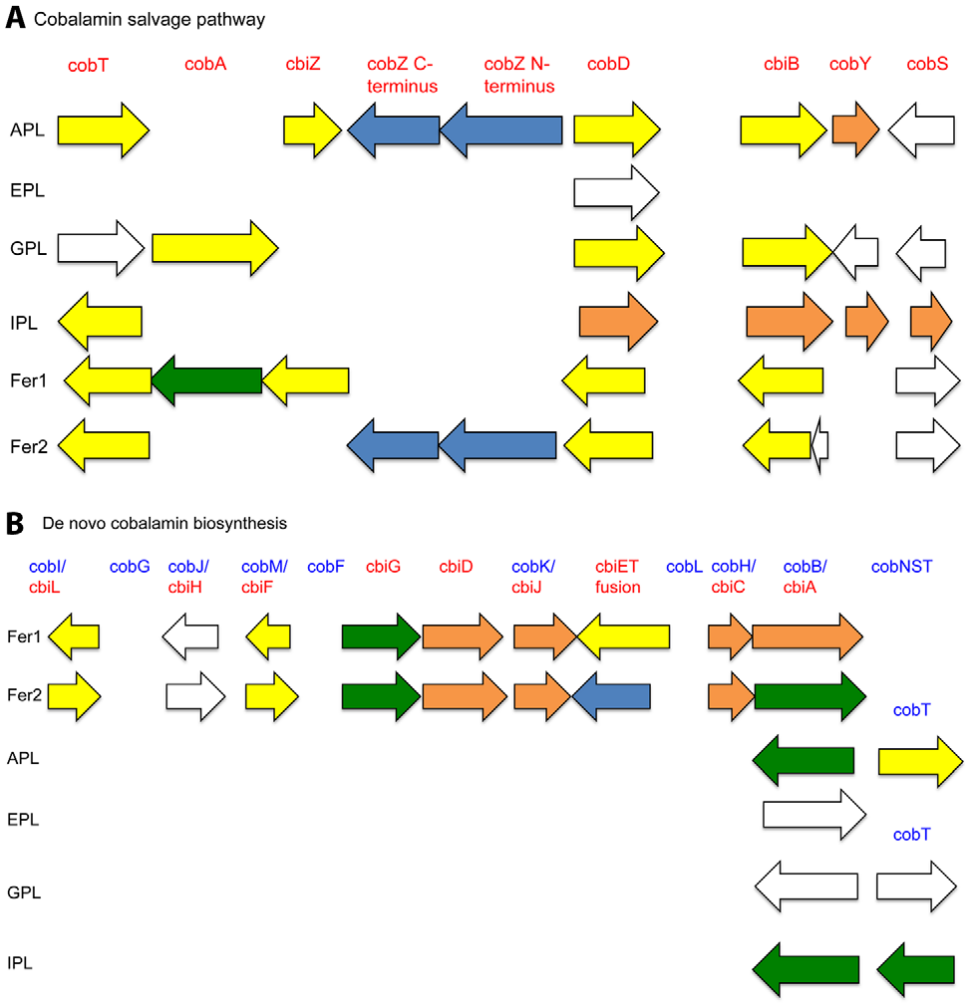
Cobalamin related genes. Arrow lengths are proportional to gene lengths. However, intergenic distances are not shown to scale. The colors indicate syntenous genes that we annotated with our synteny-based method. Genes of the same color are in the same syntenous block. Text in blue indicates de novo cobalamin synthesis genes. Text in red indicates cobalamin salvage pathway genes. A) Cobalamin salvage pathway genes. B) *De novo* cobalamin synthesis pathway genes.

We applied this evolutionary distance-weighted method to improve protein functional annotation in AMD Archaea for genes involved in the following pathways and processes i) cobalamin biosynthesis ii) molybdopterin guanine dinucleotide (MOB-DN) synthesis and MOB-DN-utilizing enzymes iii) ether lipid biosynthesis and iv) CRISPR-related proteins. We improved the annotation of 25 genes involved in cobalamin salvage in A, G, and I-plasma as well as Fer1 and Fer2 (Figure 5 and Table S2). An additional 34 genes were annotated with our method as part of the *de novo* cobalamin synthesis pathway or as cobalamin-related, including several cobalamin-binding proteins. We inferred a cobalamin-related function for two genes with very general annotations due to their synteny-based annotations (Table S2). The near complete *de novo* cobalamin synthesis pathway was found only in the Ferroplasma genomes, indicating a possible difference in these organisms' growth requirements.

**Figure 6:**
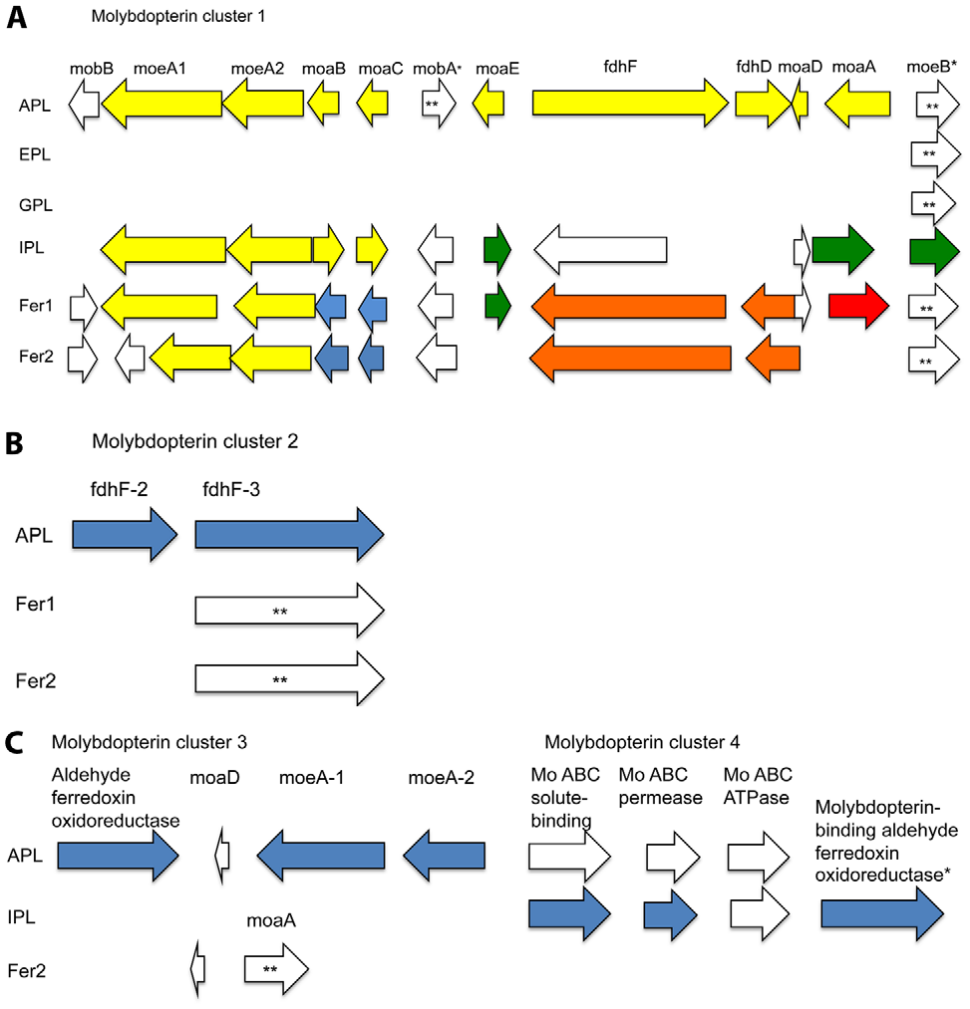
Molybdopterin synthesis clusters. Arrow lengths are proportional to gene lengths. However, intergenic distances are not shown to scale. * indicates annotations that are putative. ** indicates genes that are found outside of the cluster. Color indicates syntenous genes that we annotated with our synteny-based method and genes of the same color are in the same syntenous block. A) Cluster 1 B) Cluster 2 C) Clusters 3 and 4.

The synteny-based annotation of molybdopterin synthesis genes also differentiates the various AMD Archaea. Our synteny-based method improved annotations or provided annotations for seventeen genes in A-plasma, eleven genes in I-plasma, ten genes in Fer1, and six genes in Fer2 that were involved in molybdopterin synthesis, utilization or molybdate uptake (Figure 6 and Table S3). *In silico* protein structure modeling supported the functional annotation of a number of these genes (Table S3). The A, I, Fer1, and Fer2 genomes have full pathways for the synthesis of molybdopterin guanine dinucleotide (MOB-DN), a molybdopterin cofactor that is used by proteins involved in anaerobic energy metabolism, while E and G-plasma have very few annotated molybdopterin synthesis genes of any kind. Formate dehydrogenase subunit genes are found in A-plasma, I-plasma, Fer1, and Fer2 genomes within molybdopterin synthesis gene clusters. Formate dehydrogenase is a MOB-DN-utilizing enzyme. *In silico* protein modeling provided additional evidence for the formate dehydrogenase annotation of these genes (Table S3).

**Figure 7:**
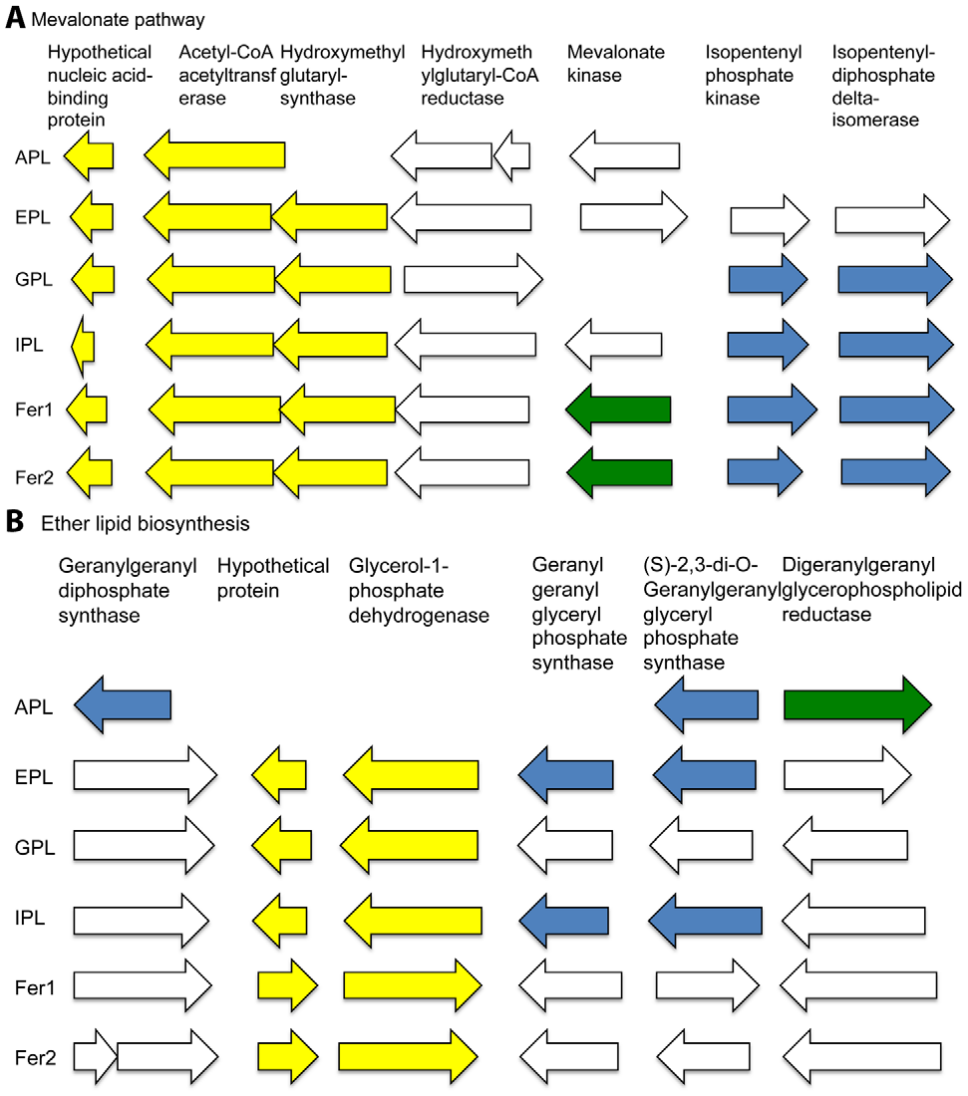
Genes related to ether lipid biosynthesis. Arrow lengths are proportional to gene lengths. However, intergenic distances are not shown to scale. The colors indicate syntenous genes that we annotated with our synteny-based method. Genes of the same color are in the same syntenous block. A) Mevalonate pathway genes B) Ether lipid biosynthesis pathway genes.

Ether lipid biosynthesis genes were found in all of the AMD *Thermoplasmatales* Archaea, as expected. Synteny-based annotation improved or provided annotations for a number of genes involved in ether lipid biosynthesis and its feeder pathway, the mevalonate pathway (Figure 7 and Table S4). This included five genes in A-plasma, seven genes in E-plasma and in G-plasma, ten genes in I-plasma, eight genes in Fer1 and eight genes in Fer2. A hypothetical protein was identified in all of the AMD Archaea studied that appeared to be associated with the mevalonate pathway based on synteny. Manual curation indicated that it may encode a nucleic-acid binding protein.

All of the AMD Archaeal genomes except for that of A-plasma contained most or all of the genes involved in the mevalonate and ether lipid biosynthesis pathways. A-plasma is missing key genes in the mevalonate pathway likely due to its incomplete genome assembly. A-plasma is also the only genome missing genes involved in the ether lipid synthesis pathway found in *Archaeoglobus fulgidus* [20]. Two genes that maintained synteny with the ether lipid synthesis genes were investigated for possible involvement in the final steps of ether lipid biosynthesis (e.g., polar head group attachment and side chain modifications). However, BLASTs against all available NCBI Bacterial genomes, indicated that these genes were also found in a number of Bacteria and were thus unlikely to be involved in Archaeal ether lipid synthesis pathways.

**Figure 8:**
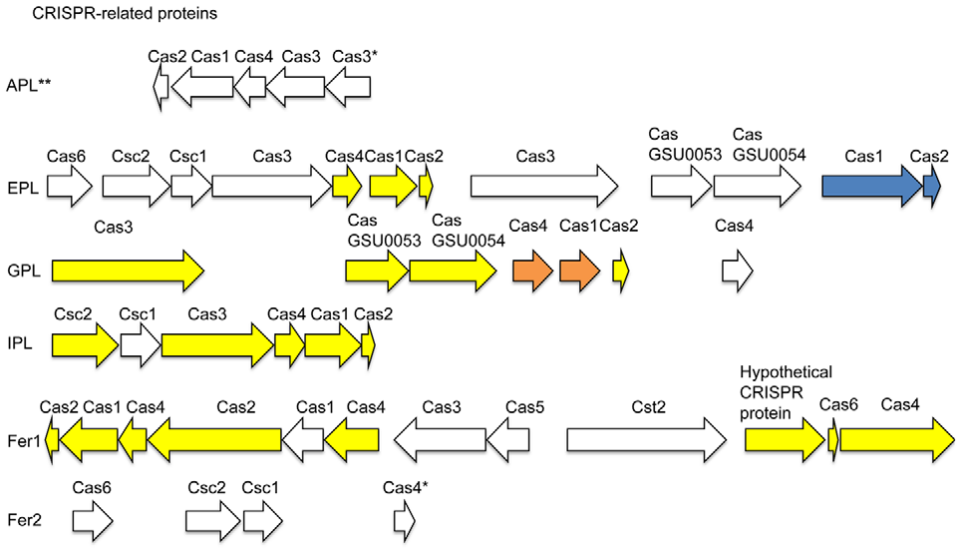
CRISPR-related protein genes. Arrow lengths are proportional to gene lengths. However, intergenic distances are not shown to scale. * indicates annotations that are putative. ** indicates a locus that is unbinned and potentially belongs in the A-plasma genome. The colors indicate syntenous genes that we annotated with our synteny-based method. Genes of the same color are in the same syntenous block.

All of the AMD *Thermoplasmatales* Archaeal genomes contain some CRISPR-associated proteins that occur in gene clusters with CRISPR spacer regions. A number of the CRISPR proteins in the AMD Archaea are syntenous with distant relatives, allowing us to improve annotations and annotate hypothetical proteins at these loci for twenty-seven CRISPR-associated proteins (Figure 8 and Table S5). All of the Archaeal genomes contained Cas1 genes, which are generally thought to be in all Cas systems as well as Cas2 genes that are found in most Cas systems [21].

### Method validation

In order to test this new synteny-based method, we compared four well characterized, very distantly related Bacteria and Archaea to one another. We made two comparisons, one between the two Bacteria and one between the two Archaea. We examined a total of 175 unique genes and their syntenous orthologs in the four organisms. Of these 175 genes we found that our method correctly annotated the genes in one organism (we chose the better characterized one in both cases) 97.1% of the time (Table S6). In five cases, the annotation appeared to be correct, but one organism had only the general annotation of ABC transporter with a likely substrate specificity instead of a specific ABC transporter protein. In three other cases, the annotations in the well characterized organism did not concur with our manual curation of the gene's function. In only two cases was the annotation method clearly incorrect, in this case substituting two very closely related protein functions that are sometimes found in the same bidirectional enzyme, fumarate reductase and succinate dehydrogenase subunits A and B.

The method was also able to reconstruct parts of the Trp operon for *E. coli* and *H. volcanii*. This is significant because not only are the functions of the genes in this operon well characterized, but their associations and regulatory systems are also well understood. In the case of *E. coli*, the method correctly predicted the functions of TrpA and TrpB (Table S8), while in the case of *H. volcanii*, the method correctly predicted the functions of TrpD, TrpE, and TrpG (Table S8).

## Discussion

We reconstructed four new genomes for acidophilic Archaea from environmental samples and compared them with the genomes of cultivated organisms. This investigation allowed us to develop a new, generally applicable, synteny-based method for improving annotations of poorly annotated genes and genes of unknown function in Bacteria and Archaea. We used this method to annotate a number of important genes in uncultivated *Thermoplasmatales* Archaeal genomes and therefore to better understand the functioning of these organisms in the AMD environment.

### Regression analysis and model selection

The trends reported here between synteny and sequence divergence and between protein functional relatedness and synteny were determined based on the phylogenetic pairwise comparison method. This method takes into account phylogeny in order to assign pairs of genomes for comparison that do not share recent phylogenetic history with other pairs. This produces phylogenetically independent data points and allows regression analysis to be carried out without pseudoreplication. We found high measures of goodness of fit for synteny and sequence divergence, as well as for synteny and percent of syntenous genes with related functions. Because there is no known mechanistic link between point mutations and gene rearrangements, these results indicate similar selective pressures on rearrangements and mutations.

Despite the advantages of the phylogenetic pairwise comparison method, we recognize that it has inherent biases. Specifically, picking the maximal number of pairs for analysis results in the choice of many closely related pairs. Pairs that are clustered in one portion of the tree may have similar levels of synteny and sequence divergence, but this correlation may be due to some third unknown trait that is also present in this clade. We chose to use all available data as opposed to more evenly spaced taxa in order to obtain enough information for regression analysis. For our analysis, we are interested in more distantly related organisms, thus partially resolving the problem of bias in close relatives. We also recognize that the use of all of the Bacterial and Archaeal genomes available in the NCBI and STRING databases has resulted in a bias in our data towards certain clades and organism types that are overrepresented (e.g., pathogens). Inclusion of genomes reconstructed from metagenomic sequence data from the natural environment slightly reduces this bias. However, this method could be greatly improved in the future when more fully sequenced genomes are available.

In a few cases, two unrelated blocks of syntenous genes were conserved adjacent to one another at significant evolutionary distances. This problem can be avoided by enforcing a stricter evolutionary distance cutoff in the cases where it can be observed that two syntenous blocks are sometimes, but not always conserved next to one another. The mechanism resulting in this type of synteny conservation is unknown.

It is important to note that the model we developed for synteny-based annotation assumes that all genes in operons are rearranged slowly compared to those that are not. This is consistent with data analysis shown in Figure 3 and with Rocha's analyses [18]. We also assumed that genes that are rearranged rapidly are not under significant selective pressure so that the trend for the non-operon genes could be used to estimate the probability that any two genes stay together due to chance. Deviation from this assumption in a subset of cases could contribute to scatter in the trend (thus poorer regressions and weaker correlations) and lead to a higher value of GOC for significance of synteny for annotation purposes. Thus, our method provides a conservative estimate of the evolutionary distance necessary for functional predictions and the probability of functional relatedness is higher than stated.

### Protein function prediction

Understanding the relationship between gene order and evolutionary distance is essential for accurate synteny-based gene functional annotation. In the case of the AMD Archaea, the weighting of conservation of gene order at large evolutionary distances resulted in improved annotations for genes involved in a number of processes, including cobalamin biosynthesis, molybdopterin guanine dinucleotide (MOB-DN) synthesis and MOB-DN-utilizing enzymes, ether lipid biosynthesis, and CRISPR-based immunity.

Synteny-based annotation of cobalamin biosynthesis genes indicated a clear difference between the nutritional requirements of A, E, G, and I-plasma versus those of the *Ferroplasma* species. Both of the Fer1 and Fer2 genomes contained full *de novo* anaerobic cobalamin synthesis pathways, while the other Archaeal genomes contained nearly complete cobalamin salvage pathways [22]. This difference may be important in differentiating the niches of the various types of AMD Archaea. It may allow the Ferroplasmas to compete better with other Archaea in low nutrient conditions, i.e., in early growth stage biofilms.

The synteny-based annotation of molybdopterin biosynthesis and molybdopterin-binding proteins in the AMD *Thermoplasmatales* Archaea also helped to differentiate their respective physiologies. The molybdopterin guanine dinucleotide synthesis protein (MobA) in A-plasma, I-plasma, Fer1, and Fer2 makes a specific type of molybdenum cofactor that is only used by dimethyl sulfoxide (DMSO) reductase family enzymes. These genomes also include a gene for a formate dehydrogenase protein (a member of the DMSO reductase family) in their molybdopterin synthesis gene clusters, indicating that they may be able to use this enzyme for nitrate reduction, mixed acid fermentation, or anaerobic carbon fixation. Previously published proteomic data demonstrate that some of these MobA genes are expressed and suggests that some AMD Archaea use one of these anaerobic energy or carbon metabolisms. E and G-plasma's genomes contain only one molybtoperin-related gene (moeB), which may be a misannotation, and thus likely do not use a MOB-DN cofactor.

*In silico* protein structure modeling supported the functional annotation of certain molybdopterin synthesis genes of interest (Table S3). Specifically, structural modeling suggested that the potential MobA genes in A-plasma and I-plasma do in fact make MobA. Interestingly, the I-plasma homolog for MoaB fits a protein model for MogA. This is intriguing because no Archaea to date have been shown to have true MogA homologs, but MoaB is thought to play the same role in molybdopterin biosynthesis for Archaea as MogA does for *E. coli* [23]. Structural modeling also supported the functional annotation of the FdhF alpha subunit genes found in the A-plasma, I-plasma, Fer1, and Fer2 genomes (Figure S3). These proteins fit the FdhF of the hydrogenase-linked formate dehydrogenase model from *Escherichia coli*, suggesting a potential involvement of these genes in a formate hydrogen lyase complex and mixed acid fermentation.

The synteny-based method identified two new genes that may be involved in MOB-DN synthesis. These are a thioredoxin family gene and a SurE: 5′/3′-nucleotidase. SurE is of particular interest, as it functions in *E. coli* to remove a phosphate group from nucleotides [24]. SurE has the highest affinity for AMP among nucleotides tested by Proudfoot *et al.* [24]. An intermediate in molydopterin biosynthesis, adenylated molybdopterin, contains a covalently-bound AMP. Thus, this SurE homolog is potentially involved in dephosphorylation related to molybdopterin biosynthesis or modification.

Ether lipid biosynthesis is a pathway common to all Archaea. The mevalonate pathway precedes ether lipid biosynthesis [20]. Thus, we looked for mevalonate pathway genes as well as ether lipid biosynthesis genes. Of the twenty-five mevalonate pathway genes annotated via our synteny-based approach, one hypothetical protein has orthologs in all of the AMD Archaea analyzed. This gene contains the PFAM domain of unknown function 35 that is hypothesized to bind nucleic acids. A possible ortholog of this gene is found in all Archaeal genomes available on NCBI, further supporting some role of this gene in the mevalonate pathway.

The CRISPR-related proteins annotated with synteny-based annotation included a number of genes previously annotated as hypothetical proteins. I-plasma and Fer1 included the typical operon configuration of Cas module family I [25], while the other genomes included novel Cas system arrangements. These annotations provide a starting point for further investigation of the biochemistry of the CRISPR/Cas system.

### Availability of computational tools

The Ruby scripts used for our analyses are open source and are available at https://github.com/pyelton/Synteny-based-annotator.

## Materials and Methods

### Sampling and genome reconstruction

For a detailed explanation of sampling, DNA extraction, sequencing and assembly protocol see Text S1. The completeness of the Archaeal genomes was evaluated based on the number of tRNA, rRNA, and other orthologous marker genes [17]. Binning accuracy was also evaluated by analysis of fragment clustering in emergent self-organizing maps (ESOM) created based on tetranucleotide frequencies of consensus contig sequences [26]. Genes missing from the pathways that we analyzed were searched for in the overall AMD DNA dataset. BLAST hits to these genes were then binned via tetranucleotide frequency, using ESOM, and assigned to organisms if possible.

### Orthology, synteny, and measures of evolutionary distance

Because the objective of this work was to analyze lineage divergence and develop a gene functional prediction method applicable to all Archaea and Bacteria, our analyses included all publicly available Archaeal genome sequences downloaded from NCBI. All available published Bacterial sequences were also added to the analysis for a more comprehensive comparison of genome rearrangements. Note that this consisted of 634 genomes because we used only the full genomes published on the NCBI website that also had full 16S rRNA sequences available on NCBI. These genomes were selected from across all major lineages of Archaea and Bacteria.

We identified orthologs and syntenous genes using pairwise comparisons between 638 organisms (Table S7) for Figures 2 and 3 and using pairwise comparisons between all 118 prokaryotic organisms from the STRING database for Figure 4 (Table S8). Orthologs were operationally defined as those genes that were reciprocal best BLASTP hits that shared 30% or greater amino acid identity over 70% or more of the gene length or BLASTP hits that shared 20% or greater amino acid identity over 50% or more of the gene length and maintained synteny. Synteny was initially defined as conservation of two or more adjacent genes in two genomes. Subsequent analyses defined synteny as the conservation of genes separated by no more than one intervening gene. Trends in synteny versus evolutionary distance did not differ substantially between these two definitions (data not shown). Thus, we generally refer to synteny in this paper as conservation of a gene pair with no more than one intervening gene.

We used an established measure of synteny, the fraction of orthologous genes that are syntenous based on at least one shared neighbor (allowing for a specified number of gene insertions) in the two genomes compared (gene order conservation; GOC) as described by Rocha [18]. For our measure of genome sequence divergence we chose average normalized BLASTP bit score normalized to the maximum possible bit score between two genes. Normalization consisted of dividing the bit score of the alignment by the average of the two maximum possible bit scores of the alignments of self to self for each respective gene (for details see Text S1). We chose this measure for two reasons. Firstly, previous work has shown that whole genome amino acid identity is a robust measure of evolutionary distance even between close relatives [27], while sequence insertions and deletions are important in sequence divergence for distant relatives [28]. Average normalized bit score is a measure that captures both insertions/deletions and amino acid identity. 16S rRNA gene sequence divergence was also considered in the analysis because it is a standard measure and for comparison to previous studies. Trends between GOC and 16S rRNA divergence were similar to those using average normalized bit score as a measure of evolutionary distance, but were more variable (data not shown).

### Manual curation

All genes used as examples in this analysis were manually curated according to the following criteria: Genes were aligned against the interpro and nr databases with a BLASTP algorithm. Genes were then annotated if they had a TIGR or Pfam domain hit that predicted a specific function with greater than 70% amino acid identity, an e-value of at least 1×10^−10^ and coverage of more than 70% of the protein. Genes were given a “putative” annotation if they met the previous criteria except they had an amino acid identity of 30–70%, an e-value between 1×10^−4^ and 1×10^−10^, and matched 50–70% of the protein, or if their domain-based hits provided only general functional information. In these cases, additional evidence from hits from the nr database was used if possible to provide a specific functional annotation. Genes were given a “probable” annotation if they had annotated hits in the nr database with greater than 30% amino acid identity over 70% of the length of the gene.

### Comparative method for correlation analysis

In order to determine the rates of synteny loss over different evolutionary distances, we looked for correlations and trends between average normalized bit score, GOC, and the percentage of syntenous genes that are known to be functionally related. Our initial regressions compared genome pairs from NCBI and our dataset and regressed GOC on average normalized bit score. The regression of percent syntenous genes that are related on GOC used genome pairs from the STRING database. Genes were considered related if they had a predicted association in STRING based on fusion events, experimental evidence, co-expression, database information (involvement in the same pathway or complex), and text mining information (co-occurrence in multiple papers). To avoid circularity in our method genome context was not used in predicting functional relatedness, that is, neither co-occurrence in genomes nor synteny was used to predict protein functional relatedness. Because of the inherent non-independence of pairwise comparisons between different taxa, we made use of a method to select phylogenetically independent pairs [19], [29]. For details on this method see Text S1.

### Operon prediction

Genes were predicted to be in operons when they had the same transcription direction and no more than thirty bases between the two. We compared genes that were in predicted operons in one or both of the two genomes in a pairwise comparison, “operon genes”, and genes that were not found in predicted operons in either genome of the pairwise comparison, “non-operon genes”. For a more detailed explanation of the operon prediction method see Text S1.

### Wilcoxon signed-rank test

In order to show that the GOC of pairwise comparisons of operon genes were significantly different from comparisons of non-operon genes, we chose to use a non-parametric test because of the unknown distribution of the data.

### Applicability to near-complete environmental genomes

In order to test the validity of this method to near-complete environmental genomes, the *Ferroplasma acidarmanus* (Fer1) isolate genome was sheared into fragments. For information on genome shearing see Text S1.

### Gene annotation

Open reading frames for the Archaeal genomes were identified using the Prodigal software [30]. Annotations were automatically generated through a pipeline that includes homology searches against KEGG and Uniref90, and domain/motif homology searches using InterProScan. Annotations were ranked in order of increasing confidence of a match: Rank A annotations are the most confident and Rank G annotations represent gene predictions with no functional assignment. For an explanation of rankings, see Text S1. All annotations specifically mentioned in this paper were manually curated based on conserved domains in InterProScan and similarity to the nr sequence database from NCBI. The remainder of the functional annotation and physiological inferences for the genomes of the AMD Archaea will be reported separately (Yelton *et al*., in preparation).

Our weighted synteny-based annotation approach is related to a previously published approach [18]. Rocha noted that GOC is an estimate of the probability that genes remain unshuffled over a certain evolutionary distancet. He also noted that genes in operons in either organism experience much slower rates of gene rearrangements than other orthologs [18]. We calculated the probability that genes are syntenous due solely to chance at a given evolutionary distance (P_GOCn_) by assuming that the GOC for rapidly shuffling genes (those not in operons; GOC_n_) was due entirely to chance. GOC_n_ was plotted against evolutionary distance and was fitted to the data by nonlinear regression.

The regressions were based on the following functions:

Average normalized bit score regression:
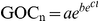
Where a, b, and c are constants, e is Euler's number, and t is the average normalized BLASTP bit score between two genomes.

Percent of syntenous genes that are related regression on P_chance_:
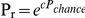
Where c is constant, e is Euler's number, and P_chance_ is the value of GOC calculated from the bit score of the comparison based on the GOC_n_ regression.

These functions were chosen for each regression based on comparison of the following types of models using Akaike's information criterion: for the GOC on average bit score regression we looked at linear models, log models, exponential models, and sigmoidal models. AIC indicated that a gompertz model fit the GOC and average bit score data the best (Table 2). This was not surprising because the data appears to be sigmoidal and asymmetrical. For the percent of functionally related syntenous genes on GOC regression we considered linear models, exponential decay models, and quadratic models. These models were forced through the point (0,1) because at a P_chance_ of zero, where the probability that two genes retain synteny due to chance is zero, the proability that sytnenous genes are functionally related must be equal to one. AIC indicated that the exponential model fit the data the best in this case (Table 2).

We found the t at GOC_n_ = P_GOCn_ = 0.05, an average normalized bit score of 0.3129, the evolutionary distance at which there is a 95% probability that syntenous genes are functionally related according to the STRING database information (Figure S1). At this evolutionary distance, there is at least a 95% probability that genes that retain synteny have done so for a reason (presumably selective pressures). In fact, the probability that syntenous genes have related function is likely higher than 95% because the STRING database does not have exhaustive protein interaction data.

Based on the derived values of t, we chose genomes that were sufficiently distant relatives that genes are not likely to be syntenous by chance so that synteny could be used to annotate genes in the AMD Archaea. For genome comparisons with a bit score of less than 0.3129, we assigned or improved annotations of genes that are found in syntenous blocks in AMD *Thermoplasmatales* Archaea. Each gene was then annotated with the annotation of its ortholog if that gene had an annotation, or as “functionally related to gene X” where gene X is its syntenous neighbor gene. If the orthologous genes in these pairs had the same annotation but one was poorly annotated, the poorly annotated gene was given an additional score that indicated a synteny-based annotation improvement.

### Method validation

Our annotation method was tested against 175 genes of known function in four genomes, two Bacterial genomes and two Archaeal genomes. The organisms used were *Escherichia coli* K12 MG1655, *Chlamydia trachomatis* D/UW-3/CX, *Haloferax volcanii*, and *Sulfolobus solfataricus* P2. These organisms were chosen for three reasons: 1. They are all very well experimentally characterized and have more than 600 articles on each of them in the ISI Web of Science database 2. They are sufficiently distant relatives that they pass the significance threshold for using our synteny-based method. It was particularly hard to find a well-characterized Bacterium that was sufficiently distant to *E. coli* K12. 3. With the exception of *Chlamydia trachomatis*, they all have genetic systems that have been used for a number of years, allowing for genetic confirmation of gene function. We chose *Chlamydia trachomatis* because it is very distant from *E. coli* and there have been recent advances in the development of a genetic system for this organism [31] that may lead to future confirmation of our findings.

The method was tested in the following manner. Syntenous orthologs were found between *Escherichia coli* K12 MG1655 and *Chlamydia trachomatis* D/UW-3/CX, and between *Haloferax volcanii* and *Sulfolobus solfataricus* P2. 88 syntenous orthologs were found between the two Bacteria and 117 syntenous orthologs were found between the two Archaea. Of these, we determined that 145 were unique to one or the other pairwise comparison based on KEGG identifiers and E.C. numbers. 30 genes were potentially shared between the two pairwise comparisons. We were able to analyze a total of 145 unique syntenous orthologs and 30 shared syntenous orthologs, thus 175 genes overall.

For these 175 syntenous orthologs, we chose to mask their function in one of the organisms in each pairwise comparison, reannotating the genes as “hypothetical proteins”. We chose to hide the functions of the genes in *E. coli* K12 in the first comparison and in *S. solfataricus* in the second comparison. We chose these organisms because they are better characterized than their pair in each case. We then took these “hypothetical proteins” and applied our synteny-based annotation method to them, determining their function solely based on the function of their counterpart in the given comparison. Then we compared the new function attributed to the “hypothetical protein” by our method to the original annotation of the protein. We considered the functions the same if they had the same KEGG identifier [32] or gene name and E.C. number in the cases where the gene did not have a KEGG identifier.

## Supporting Information

Figure S1 **Overview of the synteny-based annotation method.** 1. Comparison of GOC and average normalized bit score for NCBI database genomes. 2. Comparison of GOC and average normalized bit score for NCBI genomes split into groupins of genes found in predicted operons and non-operon genes. 3. Comparison of the percent of syntenous genes that are related and the probability that syntenous genes remain together due to chance for STRING database genomes. The green line illustrates where 95% of the syntenous genes are related. The blue line indicates the Pchance at 95% related. 4. This value is substituted into the model for evolutionary distance from the NCBI genomes to yield the average normalized bit score where 95% of syntenous genes have related functions (in red ∼0.31). 5 and 6. Based on the comparison of genomes more distantly related than this value, annotations for poorly-annotated genes are improved.
(TIF)

Figure S2 **GOC versus sequence divergence (average normalized bit score) in pairwise comparisons of genomes, including sheared Fer1 isolate genome.** The filled blue diamond indicates the comparison between Fer1 and Fer2 in the overall dataset. The open blue diamonds indicate the comparison between the fragmented Fer1 genome and the full Fer2 genome.
(TIF)

Figure S3 **Protein model of FdhF alpha subunit in I-plasma on the** ***E. coli*** **hydrogenase-linked formate dehydrogenase alpha subunit protein.** Conserved residues from active site are highlighted in red. Conserved residues from molybdenum coordinating site are highlighted in purple.
(TIF)

Table S1 **Estimation of genome completeness based on orthologous marker genes.** COG numbers and annotations for orthologous marker genes found in one copy in all prokaryotic genomes are given on the left. The number of occurrences and the gene number for each marker gene in the *Thermoplasmatales* AMD archaea are shown on the right.
(XLS)

Table S2 **Cobalamin synthesis genes and gene synteny conservation at P_related_>0.95.** Synteny conservation at P_related_>0.95 is indicated in yellow. Red indicates genes involved in the cobalamin salvage pathway whereas blue indicates genes involved in cobalamin biosynthesis.
(DOC)

Table S3 **Molybdopterin biosynthesis, utilization, and transport genes.** Synteny conservation at P_related_>0.95 is indicated in yellow. Annotations indicated in blue are for genes necessary for molybdopterin guanine dinucleotide biosynthesis. Red text indicates formate dehydrogenase genes. AS2TS model score indicates the protein structural modeling score assigned to these proteins. Only reasonable fits were included.
(DOC)

Table S4 **The mevalonate pathway and ether lipid biosynthesis genes.** Synteny conservation at P_related_>0.95 is indicated in yellow.
(DOC)

Table S5 **CRISPR-associated protein genes.** Synteny conservation at P_related_>0.95 is indicated in yellow. Gene numbers indicated in red have the same order as Cas system type 1.
(DOC)

Table S6 **Method validation with well-characterized genomes.** The query species' genes were annotated via the synteny-based method with the annotations of its ortholog in the subject species. White indicates correct annotations. Red indicates incorrect annotations. Gray indicates ambiguous cases. Bold indicates gene with shared function between the two comparisons. We only included the shared genes from the *E. coli* and *T. maritima* comparison.
(DOC)

Table S7 **List of organisms used in pairwise comparisons of GOC and sequence divergence.** Genomes were downloaded from the NCBI database or were from the in-house acid mine drainage dataset.
(DOC)

Table S8 **List of organisms used in pairwise comparisons of percentage of syntenous genes that are functionally related and GOC.** Genomes were downloaded from the STRING database.
(DOC)

Text S1 **Detailed explanation of methods.**
(DOC)
