# Supplementary figures and images for: A Semi-Quantitative, Synteny-Based Method to Improve Functional Predictions for Hypothetical and Poorly Annotated Bacterial and Archaeal Genes
Source: PLoS Comput Biol. 2011 Oct 20;7(10):e1002230. doi: 10.1371/journal.pcbi.1002230 (PMC3197636; doi:10.1371/journal.pcbi.1002230)

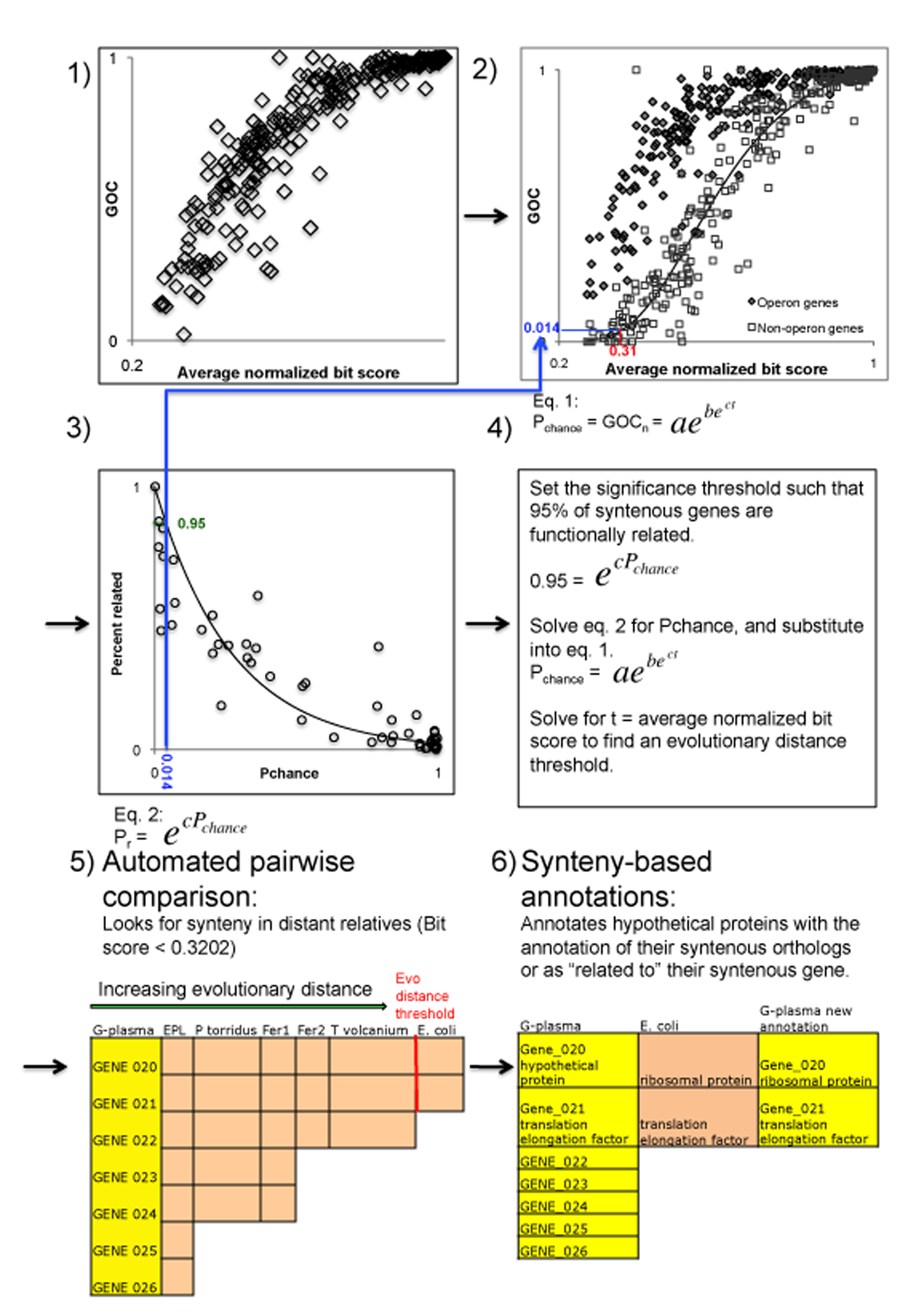

Supplement: Figure S1 — Overview of the synteny-based annotation method. 1. Comparison of GOC and average normalized bit score for NCBI database genomes. 2. Comparison of GOC and average normalized bit score for NCBI genomes split into groupins of genes found in predicted operons and non-operon genes. 3. Comparison of the percent of syntenous genes that are related and the probability that syntenous genes remain together due to chance for STRING database genomes. The green line illustrates where 95% of the syntenous genes are related. The blue line indicates the Pchance at 95% related. 4. This value is substituted into the model for evolutionary distance from the NCBI genomes to yield the average normalized bit score where 95% of syntenous genes have related functions (in red ∼0.31). 5 and 6. Based on the comparison of genomes more distantly related than this value, annotations for poorly-annotated genes are improved. (TIF) [file pcbi.1002230.s001.tif]

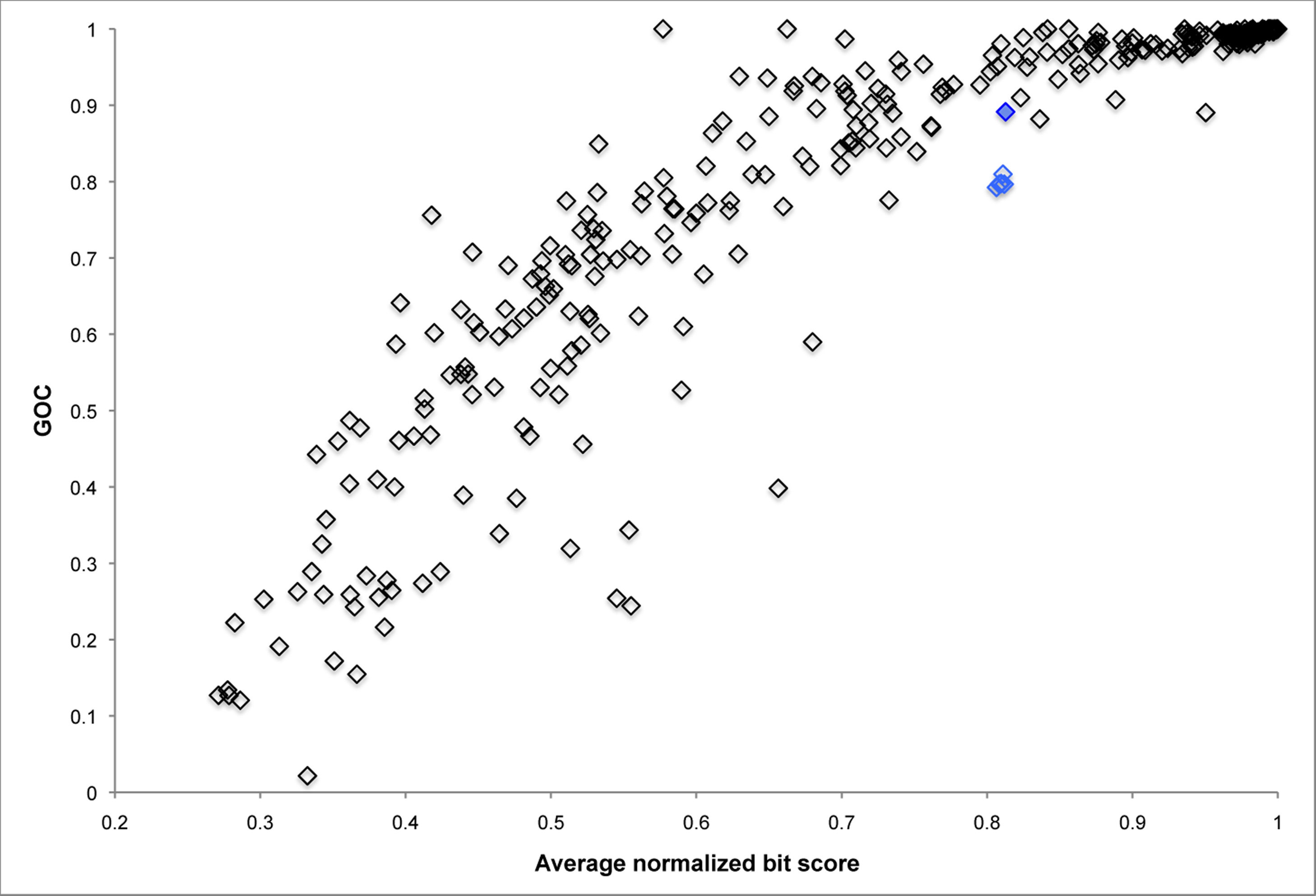

Supplement: Figure S2 — GOC versus sequence divergence (average normalized bit score) in pairwise comparisons of genomes, including sheared Fer1 isolate genome. The filled blue diamond indicates the comparison between Fer1 and Fer2 in the overall dataset. The open blue diamonds indicate the comparison between the fragmented Fer1 genome and the full Fer2 genome. (TIF) [file pcbi.1002230.s002.tif]

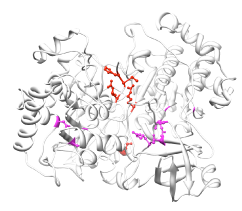

Supplement: Figure S3 — Protein model of FdhF alpha subunit in I-plasma on the E. coli hydrogenase-linked formate dehydrogenase alpha subunit protein. Conserved residues from active site are highlighted in red. Conserved residues from molybdenum coordinating site are highlighted in purple. (TIF) [file pcbi.1002230.s003.tif]
