# Supplementary material for: A Semi-Quantitative, Synteny-Based Method to Improve Functional Predictions for Hypothetical and Poorly Annotated Bacterial and Archaeal Genes
Source: PLoS Comput Biol. 2011 Oct 20;7(10):e1002230. doi: 10.1371/journal.pcbi.1002230 (PMC3197636; doi:10.1371/journal.pcbi.1002230)
Supplement: Table S2 — Cobalamin synthesis genes and gene synteny conservation at Prelated>0.95. Synteny conservation at Prelated>0.95 is indicated in yellow. Red indicates genes involved in the cobalamin salvage pathway whereas blue indicates genes involved in cobalamin biosynthesis. (DOC) [file pcbi.1002230.s005.doc]

| **Gene name** | **APL** | **GPL** | **EPL** | **IPL** | **FER1** | **FER2** |
| --- | --- | --- | --- | --- | --- | --- |
| CysG |  |  |  |  |  |  |
| CbiK |  |  |  |  |  |  |
| CobI/CbiL |  |  |  |  | fer1_1324 | fer2_scaff_72_0002 |
| CobG |  |  |  |  |  |  |
| CobJ/CbiH |  |  |  |  | fer1_0205 | fer2_scaff_557_0006 |
| CobM/CbiF |  |  |  |  | fer1_1323 | fer2_scaff_72_0003 |
| CobF |  |  |  |  |  |  |
| CbiG |  |  |  |  | fer1_1827 | fer2_scaff_18_0028 |
| CbiD |  |  |  |  | fer1_0365 | fer2_scaff_17_0018 |
| CobK/CbiJ |  |  |  |  | fer1_0363 | fer2_scaff_17_0016 |
| CbiE |  |  |  |  | fer1_1325 | fer2_scaff_618_0005 |
| CobL |  |  |  |  |  |  |
| CbiT |  |  |  |  | fer1_1325 | fer2_scaff_618_0005 |
| CobH/CbiC |  |  |  |  | fer1_0364 | fer2_scaff_17_0017 |
| CbiA/CobB | APL_17087_0043 | GPL_13334_0133 | EPL_15243_251 | IPL_15911_0376 | fer1_1828 | fer2_scaff_18_0029 |
| CbiA/CobB |  |  |  |  | fer1_1327 |  |
| CbiA N-terminus |  |  |  | IPL_13624_0310 |  |  |
| CbiA C-terminus |  |  |  | IPL_13624_0311 |  |  |
| BluB |  |  |  |  | fer1_0489 |  |
| CobNST |  |  |  |  | fer1_1606 | fer2_scaff_83_0014 |
| CobT | APL_13077_0015 | GPL_13477_0053 |  |  | fer1_1057 | fer2_scaff_47_0011 |
| CobT |  |  |  |  | fer1_1328 |  |
| CobN |  |  |  | IPL_15911_0378 |  |  |
| CobA* or CobO or BtuR |  |  |  |  | fer1_1326 |  |
| CobQ or CbiP | APL_13077_0011 | GPL_13477_0051 | EPL_15243_80 | IPL_15911_0382 | fer1_1059 | fer2_scaff_47_0014 |
| CobD1 | APL_13077_0014 | GPL_13477_0052 | EPL_15243_683 | IPL_15911_0383 | fer1_1058 | fer2_scaff_47_0015 |
| CobC | APL_13077_0013 |  |  | IPL_15911_0381 |  |  |
| CobU or CobP |  |  |  |  |  |  |
| CobS/CobS | APL_13077_0016 | GPL_13477_0049 |  |  | fer1_0558 | fer2_scaff_83_0014 |
| CobV |  |  |  | IPL_15911_0379 |  |  |
| CbiZ | APL_13077_0012 |  |  |  | fer1_1896 |  |
| PduO/EutT | APL_12068_0004 | GPL_13334_0122 | EPL_15243_46 | IPL_13624_0131 | fer1_0871 | fer2_scaff_11_0057 |
| CobD2/CbiB | APL_17325_0019 | GPL_13477_0050 |  | IPL_13624_0131 | fer1_1061 | fer2_scaff_47_0013 |
| CobD2/CbiB |  |  |  |  |  | fer2_scaff_47_0012 |
| CobY | APL_17325_0023 | GPL_13374_0175 |  | IPL_15911_0038 |  |  |
| CobZ C-terminus | APL_13077_0020 |  |  | IPL_15911_0380 |  | fer2_scaff_37_0018 |
| CobZ N-terminus | APL_13077_0021 |  |  |  |  |  |
| thioredoxin peroxidase |  |  |  | IPL_15911_0385 |  |  |
| methylmalonyl-CoA mutase, alpha subunit N-terminus | APL_13077_0002 |  |  |  |  |  |
