# Supplementary material for: A Semi-Quantitative, Synteny-Based Method to Improve Functional Predictions for Hypothetical and Poorly Annotated Bacterial and Archaeal Genes
Source: PLoS Comput Biol. 2011 Oct 20;7(10):e1002230. doi: 10.1371/journal.pcbi.1002230 (PMC3197636; doi:10.1371/journal.pcbi.1002230)
Supplement: Table S3 — Molybdopterin biosynthesis, utilization, and transport genes. Synteny conservation at Prelated>0.95 is indicated in yellow. Annotations indicated in blue are for genes necessary for molybdopterin guanine dinucleotide biosynthesis. Red text indicates formate dehydrogenase genes. AS2TS model score indicates the protein structural modeling score assigned to these proteins. Only reasonable fits were included. (DOC) [file pcbi.1002230.s006.doc]

| **Manual curation** | **APL** | **AS2TS model score** | **EPL** | **GPL** | **IPL** | **AS2TS model score** | **FER1** | **AS2TS model score** | **FER2** | **AS2TS model score** |
| --- | --- | --- | --- | --- | --- | --- | --- | --- | --- | --- |
| **Cluster 1** |  |  |  |  |  |  |  |  |  |  |
| MobB | 17445_0021 |  |  |  |  |  | 1918 |  | 17_0057 |  |
| MoeA-1 | 17445_0022 | **B** |  |  | 13606_0508 | **C1** | 1920 |  | 17_0059 |  |
| MoeA-2 | 17445_0023 | **B** |  |  | 13606_0509 | **B** | 1921 |  | 17_0060 17_0061 |  |
| moaB | 17445_0024 |  |  |  | 13606_0507 | **B** (MogA model) | 1786 |  | 64_0018 |  |
| MoaC | 17445_0025 | **A** |  |  | 13606_0506 |  | 1787 |  | 64_0019 |  |
| Possible MobA | 17445_0026 | **C1** |  |  | 13606_0517 | **B** | 1919 |  | 64_0029 |  |
| MoaE | 17445_0027 | **B** |  |  | 15911_0168 |  | 212 |  |  |  |
| fdhA1 | 17445_0030 | **C1** |  |  | 13606_0516 | **B** | 1916 | **C1** | 17_0055 | **B** |
| fdhD | 17445_0032 |  |  |  |  |  | 1914 |  | 17_0053 |  |
| moaD | 17445_0033 |  |  |  | 15911_0170 |  | 228 |  | 216_0008 |  |
| moaA | 17445_0034 |  | 17965_270 |  | 15911_0169 |  | 227 |  | 216_0009 |  |
| moaA |  |  |  |  |  |  |  |  | 9_0006 |  |
| **Cluster 3** |  |  |  |  |  |  |  |  |  |  |
| fdhA2 | 17306_0015 |  |  |  |  |  | 549 |  | 5_0011 | **B** |
| formate dehydrogenase (based on synteny) | 17306_0014 |  |  |  |  |  |  |  |  |  |
| **Cluster 4** |  |  |  |  |  |  |  |  |  |  |
| Aldehyde ferredoxin oxidoreductase with molybdopterin cofactor (based on synteny) | 17112_0007 |  |  |  |  |  |  |  |  |  |
| MoaD | 17112_0008 | **A** |  |  |  |  |  |  | 217_0004 |  |
| MoeA-1 | 17112_0010 | **C1** |  |  |  |  |  |  |  |  |
| MoeA-2 | 17112_0011 | **B** |  |  |  |  |  |  |  |  |
| MoaA |  |  |  |  |  |  |  |  | 217_0005 |  |
| tungstate/molybdate binding ABC transporter solute-binding component | 17112_0004 |  |  |  | 13606_0512 |  |  |  |  |  |
| molybdate ABC-transporter permease component | 17112_0005 |  |  |  | 13606_0511 | **B** |  |  |  |  |
| molybdate ABC-transporter ATPase component | 17112_0006 |  | 17965_470 |  | 13606_0510 |  |  |  |  |  |
| tungstate/molybdopterin-binding aldehyde ferredoxin oxidoreductase | 17112_0007 |  |  |  | 13606_0513 |  |  |  |  |  |
| Possible MoeB | 17454_0009 |  |  | 13290_0005 | 15911_0171 |  | 994 |  | 24_0011 |  |
| Possible MoeB | 17452_0003 |  |  |  |  |  |  |  | 863_0003 |  |
| thioredoxin related to molybdopterin synthesis |  |  | 15243_750 |  |  |  | 210 |  |  |  |
| surE: 5'/3'-nucleotidase SurE |  |  |  |  |  |  | 224 |  |  |  |
