# Supplementary material for: A Semi-Quantitative, Synteny-Based Method to Improve Functional Predictions for Hypothetical and Poorly Annotated Bacterial and Archaeal Genes
Source: PLoS Comput Biol. 2011 Oct 20;7(10):e1002230. doi: 10.1371/journal.pcbi.1002230 (PMC3197636; doi:10.1371/journal.pcbi.1002230)
Supplement: Table S4 — The mevalonate pathway and ether lipid biosynthesis genes. Synteny conservation at Prelated>0.95 is indicated in yellow. (DOC) [file pcbi.1002230.s007.doc]

| **Annotation** | **E.C.** | **APL** | **EPL** | **GPL** | **FER1** | **FER2** | **IPL** |
| --- | --- | --- | --- | --- | --- | --- | --- |
| **Mevalonate pathway** |  |  |  |  |  |  |  |
| hypothetical nucleic acid-binding protein likely involved in mevalonate pathway |  | 17433_0048 | 15243_104 | 13459_0238 | 1029 | 20_0023 | 13624_0355 |
| Acetyl-CoA acetyltransferase | 2.3.1.9 | 17433_0047 | 15243_105 | 13459_0237 | 1028 | 20_0024 | 13624_0356 |
| hydroxymethylglutaryl-CoA synthase | 2.3.3.10 |  | 15243_106 | 13459_0236 | 1027 | 20_0025 | 13624_0357 |
| hydroxymethylglutaryl-CoA reductase | 1.1.1.88 | 17387_0042 | 17965_86 | 13477_0039 |  | 82_0017 | 13624_0213 |
| hydroxymethylglutaryl-CoA reductase | 1.1.1.88 or | 17387_0043 |  |  |  |  |  |
| hydroxymethylglutaryl-CoA reductase | 1.1.1.34 |  |  |  | 271 |  |  |
| Mevalonate kinase | 2.7.1.36 | 12885_0014 | 15243_764 |  | 294 | 107_0008 | 13606_0039 |
| isopentenyl phosphate kinase |  |  | 12876_19* | 13334_0003 | 945 | 97_0016 | 13249_0046 |
| isopentenyl-diphosphate delta-isomerase | 5.3.3.2 |  | 12876_20 | 13334_0004 | 944 | 97_0015 | 13249_0045 |
|  |  |  |  |  |  |  |  |
| **To ether lipid biosynthesis (A. fulgidus pathway)** |  |  |  |  |  |  |  |
| geranylgeranyl diphosphate synthase | 2.5.1.29 | 17387_0030 | 17965_136 | 13459_0158 | 1283 | 56_0006 56_0007 | 13606_0087 |
| hypothetical protein likely involved in ether lipid synthesis |  |  | 15243_0189 | 13459_0274 | 1278 | 56_0002 | 13624_0051 |
| glycerol-1-phosphate dehydrogenase | 1.1.1.261 |  | 15243_188 | 13459_0275 | 1280 | 56_0003* | 13624_0050 |
| geranylgeranylglyceryl phosphate synthase | 2.5.1.41 |  | 17965_136 | 13327_0027 | 508 | 9_0060 | 13606_0280 |
| (S)-2,3-di-O-geranylgeranylglyceryl phosphate synthase | 2.5.1.42 | 17387_0029 | 17965_135 | 13327_0026 | 303 | 107_0020 | 13606_0281 |
| Digeranylgeranylglycerophospholipid reductase | 1.3.1.- | 17087_0052 |  | 13459_0113 check BLAST with proteins on bakerite | 166 | 18_0018 | 13624_0101 |
