# Supplementary material for: A Semi-Quantitative, Synteny-Based Method to Improve Functional Predictions for Hypothetical and Poorly Annotated Bacterial and Archaeal Genes
Source: PLoS Comput Biol. 2011 Oct 20;7(10):e1002230. doi: 10.1371/journal.pcbi.1002230 (PMC3197636; doi:10.1371/journal.pcbi.1002230)
Supplement: Table S5 — CRISPR-associated protein genes. Synteny conservation at Prelated>0.95 is indicated in yellow. Gene numbers indicated in red have the same order as Cas system type 1. (DOC) [file pcbi.1002230.s008.doc]

| **Cas gene** | **APL** | **EPL** | **GPL** | **IPL** | **FER1** | **FER2** |
| --- | --- | --- | --- | --- | --- | --- |
| Cas6 | 00190_7 | 15243_144 |  |  | 1216 | 362_0002 |
| Csc2 | 00190_10 | 15243_146 |  | 15911_0446 |  | 480_0004 |
| Csc1 | 00190_11 | 15243_147 |  | 15911_0445 | 1223 | 480_0003 |
| Cas3 | 00190_12 | 15243_148 | 13455_0101 |  | 1220 |  |
| Cas4 | 00190_13 | 15243_149 | 13455_0105 |  | 1221 | 480_0001 |
| Cas1 | 00190_14 | 15243_151 | 13455_0106 |  | 0003 |  |
| hypothetical CRISPR-protein |  |  |  |  | 0011 |  |
| Cst2 |  |  |  |  | 0010 |  |
| Cas5 |  | 17965_118 |  |  | 0009 |  |
| Cas3 | 0004 | 15243_155 |  | 15911_0444 | 0008 |  |
| Cas4 | 0003 | 17965_341 | 13290_0050 | 15911_0443 | 0007 |  |
| Cas1 | 0002 | 15243_159 |  | 15911_0442 | 0006 |  |
| Cas2 | 0001 partial | 15243_160 partial |  | 15911_0441 partial | 0005 full |  |
| Cas GSU0053 |  | 15243_157 | 13455_0103 |  |  |  |
| Cas GSU0054 |  | 15243_158 | 13455_0104 |  |  |  |
| Cas4 |  |  |  |  | 0013 |  |
| Cas4 |  |  |  |  | 0004 |  |
| Csh2 |  |  |  |  | 1218 |  |
| Cas5 |  |  |  |  | 1219 |  |
| Cas3 |  |  |  |  | 1220 |  |
| Cas2 | 00190_15 partial |  |  |  | 0002 full |  |
| Csh1 |  |  |  |  | 1217 |  |
| Cas1 |  |  |  |  | 1225 |  |
