# Supplementary material for: A Semi-Quantitative, Synteny-Based Method to Improve Functional Predictions for Hypothetical and Poorly Annotated Bacterial and Archaeal Genes
Source: PLoS Comput Biol. 2011 Oct 20;7(10):e1002230. doi: 10.1371/journal.pcbi.1002230 (PMC3197636; doi:10.1371/journal.pcbi.1002230)
Supplement: Table S6 — Method validation with well-characterized genomes. The query species' genes were annotated via the synteny-based method with the annotations of its ortholog in the subject species. White indicates correct annotations. Red indicates incorrect annotations. Gray indicates ambiguous cases. Bold indicates gene with shared function between the two comparisons. We only included the shared genes from the E. coli and T. maritima comparison. (DOC) [file pcbi.1002230.s009.doc]

| **Organism name** |
| --- |
| A-plasma |
| Acaryochloris_marina_MBIC11017 |
| Acholeplasma_laidlawii_PG_8A |
| Acidithiobacillus_ferrooxidans_ATCC_23270 |
| Acidithiobacillus_ferrooxidans_ATCC_53993 |
| Acidothermus_cellulolyticus_11B |
| Acidovorax_citrulli_AAC00_1 |
| Aciduliprofundum_boonei_T469 |
| Acinetobacter_baumannii_AB307_0294 |
| Acinetobacter_sp_ADP1 |
| Actinobacillus_pleuropneumoniae_serovar_3_JL03 |
| Actinobacillus_pleuropneumoniae_serovar_5b_L20 |
| Actinobacillus_pleuropneumoniae_serovar_7_AP76 |
| Actinobacillus_succinogenes_130Z |
| Aeromonas_hydrophila_ATCC_7966 |
| Aeropyrum_pernix_K1 |
| Agrobacterium_tumefaciens_C58 |
| Akkermansia_muciniphila_ATCC_BAA_835 |
| Alcanivorax_borkumensis_SK2 |
| Alkalilimnicola_ehrlichii_MLHE_1 |
| Alkaliphilus_metalliredigens_QYMF |
| Alkaliphilus_oremlandii_OhILAs |
| Alteromonas_macleodii__Deep_ecotype_ |
| Anaeromyxobacter_dehalogenans_2CP-C |
| Anaeromyxobacter_Fw109-5 |
| Anaeromyxobacter_K |
| Anaplasma_marginale_Maries |
| Anaplasma_phagocytophilum_HZ |
| Anoxybacillus_flavithermus_WK1 |
| Aquifex_aeolicus_VF5 |
| Archaeoglobus_fulgidus |
| Arcobacter_butzleri_RM4018 |
| Aromatoleum_aromaticum_EbN1 |
| Arthrobacter_aurescens_TC1 |
| Aster_yellows_witches_broom_phytoplasma_AYWB |
| Azoarcus_BH72 |
| Azorhizobium_caulinodans_ORS_571 |
| Bacillus_amyloliquefaciens_FZB42 |
| Bacillus_anthracis_Ames |
| Bacillus_anthracis_str_Sterne |
| Bacillus_cereus_ATCC_10987 |
| Bacillus_cereus_B4264 |
| Bacillus_cereus_E33L |
| Bacillus_cereus_G9842 |
| Bacillus_clausii_KSM-K16 |
| Bacillus_halodurans_C_125 |
| Bacillus_licheniformis_ATCC_14580 |
| Bacillus_licheniformis_DSM_13 |
| Bacillus_pumilus_SAFR_032 |
| Bacillus_subtilis |
| Bacillus_thuringiensis_konkukian |
| Bacteroides_fragilis_NCTC_9343 |
| Bacteroides_thetaiotaomicron_VPI-5482 |
| Bacteroides_vulgatus_ATCC_8482 |
| Bartonella_bacilliformis_KC583 |
| Bartonella_henselae_Houston_1 |
| Bartonella_quintana_Toulouse |
| Baumannia_cicadellinicola_Hc__Homalodisca_coagulata_ |
| Bdellovibrio_bacteriovorus |
| Bifidobacterium_adolescentis_ATCC_15703 |
| Bifidobacterium_animalis_lactis_AD011 |
| Bifidobacterium_longum_infantis_ATCC_15697 |
| Bifidobacterium_longum_NCC2705 |
| Bordetella_avium_197N |
| Bordetella_bronchiseptica_RB50 |
| Bordetella_parapertussis |
| Bordetella_pertussis_Tohama_I |
| Bordetella_petrii |
| Borrelia_hermsii_DAH |
| Borrelia_recurrentis_A1 |
| Borrelia_turicatae_91E135 |
| Bradyrhizobium_japonicum |
| Bradyrhizobium_ORS278 |
| Brucella_abortus_bv__1_9_941 |
| Brucella_abortus_S19 |
| Brucella_canis_ATCC_23365 |
| Brucella_melitensis_biovar_Abortus |
| Brucella_melitensis_bv__1_16M |
| Brucella_ovis |
| Brucella_suis_1330 |
| Buchnera_aphidicola_5A__Acyrthosiphon_pisum_ |
| Buchnera_aphidicola_Bp__Baizongia_pistaciae_ |
| Buchnera_aphidicola_Cc_Cinara_cedri |
| Buchnera_aphidicola_Sg |
| Buchnera_aphidicola_Tuc7__Acyrthosiphon_pisum_ |
| Burkholderia_383 |
| Burkholderia_ambifaria_MC40_6 |
| Burkholderia_cenocepacia_AU_1054 |
| Burkholderia_cenocepacia_HI2424 |
| Burkholderia_cenocepacia_J2315 |
| Burkholderia_cenocepacia_MC0_3 |
| Burkholderia_mallei_ATCC_23344 |
| Burkholderia_mallei_NCTC_10229 |
| Burkholderia_mallei_NCTC_10247 |
| Burkholderia_multivorans_ATCC_17616_Tohoku |
| Burkholderia_phymatum_STM815 |
| Burkholderia_phytofirmans_PsJN |
| Burkholderia_pseudomallei_1106a |
| Burkholderia_pseudomallei_1710b |
| Burkholderia_pseudomallei_668 |
| Burkholderia_pseudomallei_K96243 |
| Burkholderia_thailandensis_E264 |
| Burkholderia_xenovorans_LB400 |
| Caldicellulosiruptor_saccharolyticus_DSM_8903 |
| Campylobacter_fetus_82-40 |
| Campylobacter_jejuni_81116 |
| Campylobacter_jejuni_doylei_269_97 |
| Campylobacter_jejuni_NCTC_11168 |
| Campylobacter_jejuni_RM1221 |
| Candidatus_Amoebophilus_asiaticus_5a2 |
| Candidatus_Blochmannia_floridanus |
| Candidatus_Blochmannia_pennsylvanicus_BPEN |
| Candidatus_Desulforudis_audaxviator_MP104C |
| Candidatus_Korarchaeum_cryptofilum_OPF8 |
| Candidatus_Koribacter_versatilis_Ellin345 |
| Candidatus_Methanoregula_boonei_6A8 |
| Candidatus_Methanosphaerula_palustris_E1_9c |
| Candidatus_Pelagibacter_ubique_HTCC1062 |
| Candidatus_Phytoplasma_australiense |
| Candidatus_Phytoplasma_mali |
| Candidatus_Protochlamydia_amoebophila_UWE25 |
| Candidatus_Ruthia_magnifica_Cm__Calyptogena_magnifica_ |
| Candidatus_Sulcia_muelleri_GWSS |
| Candidatus_Vesicomyosocius_okutanii_HA |
| Carboxydothermus_hydrogenoformans_Z_2901 |
| Caulobacter_crescentus |
| Cellvibrio_japonicus_Ueda107 |
| Chlamydia_trachomatis_434_Bu |
| Chlamydia_trachomatis_A_HAR-13 |
| Chlamydia_trachomatis_D_UW_3_CX |
| Chlamydia_trachomatis_L2b_UCH_1_proctitis |
| Chlamydophila_abortus_S26_3 |
| Chlamydophila_caviae |
| Chlamydophila_felis_Fe_C-56 |
| Chlamydophila_pneumoniae_AR39 |
| Chlamydophila_pneumoniae_CWL029 |
| Chlamydophila_pneumoniae_J138 |
| Chlamydophila_pneumoniae_TW_183 |
| Chlorobaculum_parvum_NCIB_8327 |
| Chlorobium_chlorochromatii_CaD3 |
| Chlorobium_limicola_DSM_245 |
| Chlorobium_luteolum_DSM_273 |
| Chlorobium_phaeobacteroides_BS1 |
| Chlorobium_phaeobacteroides_DSM_266 |
| Chlorobium_tepidum_TLS |
| Chloroflexus_aggregans_DSM_9485 |
| Chloroflexus_aurantiacus_J_10_fl |
| Chloroherpeton_thalassium_ATCC_35110 |
| Chromobacterium_violaceum_ATCC_12472 |
| Chromohalobacter_salexigens_DSM_3043 |
| Citrobacter_koseri_ATCC_BAA_895 |
| Clostridium_beijerinckii_NCIMB_8052 |
| Clostridium_botulinum_A |
| Clostridium_botulinum_A_ATCC_19397 |
| Clostridium_botulinum_A_Hall |
| Clostridium_botulinum_B_Eklund_17B |
| Clostridium_botulinum_E3_Alaska_E43 |
| Clostridium_botulinum_F_Langeland |
| Clostridium_novyi_NT |
| Clostridium_perfringens_ATCC_13124 |
| Clostridium_perfringens_SM101 |
| Clostridium_phytofermentans_ISDg |
| Clostridium_tetani_E88 |
| Clostridium_thermocellum_ATCC_27405 |
| Colwellia_psychrerythraea_34H |
| Coprothermobacter_proteolyticus_DSM_5265 |
| Corynebacterium_diphtheriae_NCTC_13129 |
| Corynebacterium_efficiens_YS-314 |
| Corynebacterium_glutamicum_ATCC_13032_Bielefeld |
| Corynebacterium_glutamicum_ATCC_13032_Kitasato |
| Corynebacterium_glutamicum_R |
| Corynebacterium_urealyticum_DSM_7109 |
| Coxiella_burnetii_CbuG_Q212 |
| Coxiella_burnetii_RSA_493 |
| Cupriavidus_taiwanensis |
| Cyanobacteria_bacterium_Yellowstone_B-Prime |
| Cyanothece_PCC_7424 |
| Cytophaga_hutchinsonii_ATCC_33406 |
| Dechloromonas_aromatica_RCB |
| Dehalococcoides_BAV1 |
| Dehalococcoides_CBDB1 |
| Dehalococcoides_ethenogenes_195 |
| Deinococcus_geothermalis_DSM_11300 |
| Delftia_acidovorans_SPH-1 |
| Desulfatibacillum_alkenivorans_AK_01 |
| Desulfitobacterium_hafniense_DCB_2 |
| Desulfitobacterium_hafniense_Y51 |
| Desulfococcus_oleovorans_Hxd3 |
| Desulfotalea_psychrophila_LSv54 |
| Desulfotomaculum_reducens_MI_1 |
| Desulfovibrio_desulfuricans_G20 |
| Desulfovibrio_vulgaris__Miyazaki_F_ |
| Desulfovibrio_vulgaris_Hildenborough |
| Desulfurococcus_kamchatkensis_1221n |
| Dictyoglomus_thermophilum_H_6_12 |
| Dictyoglomus_turgidum_DSM_6724 |
| Dinoroseobacter_shibae_DFL_12 |
| E-plasma |
| Ehrlichia_canis_Jake |
| Ehrlichia_chaffeensis_Arkansas |
| Ehrlichia_ruminantium_Gardel |
| Ehrlichia_ruminantium_str._Welgevonden_CIRAD |
| Ehrlichia_ruminantium_Welgevonden_UPSA |
| Elusimicrobium_minutum_Pei191 |
| Enterobacter_sakazakii_ATCC_BAA-894 |
| Enterococcus_faecalis_V583 |
| Erwinia_carotovora_atroseptica_SCRI1043 |
| Erythrobacter_litoralis_HTCC2594 |
| Escherichia_coli_536 |
| Escherichia_coli_55989 |
| Escherichia_coli_APEC_O1 |
| Escherichia_coli_C_ATCC_8739 |
| Escherichia_coli_CFT073 |
| Escherichia_coli_ED1a |
| Escherichia_coli_HS |
| Escherichia_coli_IAI1 |
| Escherichia_coli_IAI39 |
| Escherichia_coli_K_12_substr__DH10B |
| Escherichia_coli_K_12_substr__MG1655 |
| Escherichia_coli_O127_H6_E2348_69 |
| Escherichia_coli_O157_H7_EDL933 |
| Escherichia_coli_S88 |
| Escherichia_coli_UMN026 |
| Escherichia_fergusonii_ATCC_35469 |
| Ferroplasma acidarmanus I |
| Ferroplasma acidarmanus II |
| Fervidobacterium_nodosum_Rt17-B1 |
| Flavobacterium_johnsoniae_UW101 |
| Flavobacterium_psychrophilum_JIP02_86 |
| Francisella_tularensis_FSC198 |
| Francisella_tularensis_holarctica_FTNF002_00 |
| Francisella_tularensis_holarctica_LVS |
| Francisella_tularensis_holarctica_OSU18 |
| Francisella_tularensis_mediasiatica_FSC147 |
| Francisella_tularensis_novicida_U112 |
| Francisella_tularensis_tularensis |
| Francisella_tularensis_WY96-3418 |
| Frankia_alni_ACN14a |
| Frankia_CcI3 |
| Frankia_EAN1pec |
| Fusobacterium_nucleatum_ATCC_25586 |
| G-plasma |
| Geobacillus_thermodenitrificans_NG80-2 |
| Geobacter_bemidjiensis_Bem |
| Geobacter_lovleyi_SZ |
| Geobacter_sulfurreducens_PCA |
| Geobacter_uraniumreducens_Rf4 |
| Gloeobacter_violaceus_PCC_7421 |
| Gluconacetobacter_diazotrophicus_PAl_5 |
| Gramella_forsetii_KT0803 |
| Granulobacter_bethesdensis_CGDNIH1 |
| Haemophilus_ducreyi_35000HP |
| Haemophilus_influenzae_86_028NP |
| Haemophilus_influenzae_PittEE |
| Haemophilus_influenzae_PittGG |
| Haemophilus_influenzae_Rd_KW20 |
| Haemophilus_parasuis_SH0165 |
| Haemophilus_somnus_2336 |
| Hahella_chejuensis_KCTC_2396 |
| Halobacterium_salinarum_R1 |
| Haloquadratum_walsbyi_DSM_16790 |
| Halorhodospira_halophila_SL1 |
| Helicobacter_acinonychis_Sheeba |
| Helicobacter_hepaticus_ATCC_51449 |
| Helicobacter_pylori_26695 |
| Helicobacter_pylori_G27 |
| Helicobacter_pylori_HPAG1 |
| Helicobacter_pylori_J99 |
| Helicobacter_pylori_P12 |
| Helicobacter_pylori_Shi470 |
| Heliobacterium_modesticaldum_Ice1 |
| Herpetosiphon_aurantiacus_ATCC_23779 |
| Hydrogenobaculum_Y04AAS1 |
| Hyperthermus_butylicus |
| Hyphomonas_neptunium_ATCC_15444 |
| I-plasma |
| Idiomarina_loihiensis_L2TR |
| Ignicoccus_hospitalis_KIN4_I |
| Janthinobacterium_Marseille |
| Kocuria_rhizophila_DC2201 |
| Lactobacillus_acidophilus_NCFM |
| Lactobacillus_brevis_ATCC_367 |
| Lactobacillus_casei |
| Lactobacillus_delbrueckii_bulgaricus_ATCC_11842 |
| Lactobacillus_delbrueckii_bulgaricus_ATCC_BAA-365 |
| Lactobacillus_fermentum_IFO_3956 |
| Lactobacillus_gasseri_ATCC_33323 |
| Lactobacillus_helveticus_DPC_4571 |
| Lactobacillus_johnsonii_NCC_533 |
| Lactobacillus_plantarum |
| Lactobacillus_reuteri_DSM_20016 |
| Lactobacillus_reuteri_F275_Kitasato |
| Lactobacillus_sakei_23K |
| Lactococcus_lactis_cremoris_MG1363 |
| Lactococcus_lactis_Il1403 |
| Lawsonia_intracellularis_PHE_MN1-00 |
| Legionella_pneumophila_Corby |
| Legionella_pneumophila_Philadelphia_1 |
| Leifsonia_xyli_xyli_CTCB0 |
| Leptospira_biflexa_serovar_Patoc__Patoc_1__Ames_ |
| Leptospira_biflexa_serovar_Patoc__Patoc_1__Paris_ |
| Leptospira_borgpetersenii_serovar_Hardjo-bovis_JB197 |
| Leptospira_borgpetersenii_serovar_Hardjo-bovis_L550 |
| Leptospira_interrogans_serovar_Lai |
| Leptospira_interrogans_serovar_Lai_56601 |
| Leptothrix_cholodnii_SP_6 |
| Listeria_innocua |
| Listeria_monocytogenes |
| Listeria_monocytogenes_4b_F2365 |
| Listeria_monocytogenes_HCC23 |
| Listeria_welshimeri_serovar_6b_SLCC5334 |
| Magnetococcus_MC_1 |
| Magnetospirillum_magneticum_AMB_1 |
| Mannheimia_succiniciproducens_MBEL55E |
| Maricaulis_maris_MCS10 |
| Marinomonas_MWYL1 |
| Mesoplasma_florum_L1 |
| Mesorhizobium_loti |
| Metallosphaera_sedula_DSM_5348 |
| Methanobrevibacter_smithii_ATCC_35061 |
| Methanococcoides_burtonii_DSM_6242 |
| Methanococcus_aeolicus_Nankai_3 |
| Methanococcus_jannaschii |
| Methanococcus_maripaludis_C5 |
| Methanococcus_maripaludis_C6 |
| Methanococcus_maripaludis_C7 |
| Methanococcus_maripaludis_S2 |
| Methanococcus_vannielii_SB |
| Methanocorpusculum_labreanum_Z |
| Methanoculleus_marisnigri_JR1 |
| Methanopyrus_kandleri |
| Methanosaeta_thermophila_PT |
| Methanosarcina_acetivorans_C2A |
| Methanosarcina_mazei |
| Methanosphaera_stadtmanae_DSM_3091 |
| Methanospirillum_hungatei_JF_1 |
| Methanothermobacter_thermautotrophicus_Delta_H |
| Methylacidiphilum_infernorum_V4 |
| Methylibium_petroleiphilum_PM1 |
| Methylobacillus_flagellatus_KT |
| Methylobacterium_chloromethanicum_CM4 |
| Methylobacterium_extorquens_PA1 |
| Methylocella_silvestris_BL2 |
| Methylococcus_capsulatus_Bath |
| Microcystis_aeruginosa_NIES_843 |
| Moorella_thermoacetica_ATCC_39073 |
| Mycobacterium_avium_104 |
| Mycobacterium_avium_paratuberculosis |
| Mycobacterium_bovis_AF2122_97 |
| Mycobacterium_bovis_BCG_Pasteur_1173P2 |
| Mycobacterium_gilvum_PYR_GCK |
| Mycobacterium_JLS |
| Mycobacterium_leprae_TN |
| Mycobacterium_MCS |
| Mycobacterium_smegmatis_MC2_155 |
| Mycobacterium_tuberculosis_F11 |
| Mycobacterium_tuberculosis_H37Ra |
| Mycobacterium_ulcerans_Agy99 |
| Mycobacterium_vanbaalenii_PYR-1 |
| Mycoplasma_agalactiae_PG2 |
| Mycoplasma_arthritidis_158L3_1 |
| Mycoplasma_capricolum_ATCC_27343 |
| Mycoplasma_gallisepticum |
| Mycoplasma_genitalium_G37 |
| Mycoplasma_hyopneumoniae_232 |
| Mycoplasma_hyopneumoniae_7448 |
| Mycoplasma_hyopneumoniae_J |
| Mycoplasma_mobile_163K |
| Mycoplasma_mycoides |
| Mycoplasma_penetrans_HF_2 |
| Mycoplasma_pneumoniae |
| Mycoplasma_pulmonis |
| Mycoplasma_synoviae_53 |
| Myxococcus_xanthus_DK_1622 |
| Nanoarchaeum_equitans_Kin4_M |
| Natronomonas_pharaonis |
| Neisseria_gonorrhoeae_FA_1090 |
| Neisseria_meningitidis_FAM18 |
| Neisseria_meningitidis_MC58 |
| Neisseria_meningitidis_Z2491 |
| Neorickettsia_sennetsu_Miyayama |
| Nitratiruptor_SB155_2 |
| Nitrobacter_winogradskyi_Nb_255 |
| Nitrosomonas_europaea_ATCC_19718 |
| Nitrosopumilus_maritimus_SCM1 |
| Nitrosospira_multiformis_ATCC_25196 |
| Nocardia_farcinica_IFM_10152 |
| Nostoc_punctiforme_PCC_73102 |
| Novosphingobium_aromaticivorans_DSM_12444 |
| Oceanobacillus_iheyensis_HTE831 |
| Ochrobactrum_anthropi_ATCC_49188 |
| Oenococcus_oeni_PSU-1 |
| Oligotropha_carboxidovorans_OM5 |
| Onion_yellows_phytoplasma |
| Opitutus_terrae_PB90_1 |
| Orientia_tsutsugamushi_Boryong |
| Orientia_tsutsugamushi_Ikeda |
| Parabacteroides_distasonis_ATCC_8503 |
| Paracoccus_denitrificans_PD1222 |
| Parvibaculum_lavamentivorans_DS_1 |
| Pasteurella_multocida_Pm70 |
| Pediococcus_pentosaceus_ATCC_25745 |
| Pelobacter_carbinolicus |
| Pelodictyon_phaeoclathratiforme_BU_1 |
| Pelotomaculum_thermopropionicum_SI |
| Petrotoga_mobilis_SJ95 |
| Photorhabdus_luminescens |
| Picrophilus_torridus_DSM_9790 |
| Pirellula_sp |
| Polaromonas_JS666 |
| Polynucleobacter_necessarius_asymbioticus_QLW_P1DMWA_1 |
| Polynucleobacter_necessarius_STIR1 |
| Porphyromonas_gingivalis_ATCC_33277 |
| Porphyromonas_gingivalis_W83 |
| Prochlorococcus_marinus_AS9601 |
| Prochlorococcus_marinus_CCMP1375 |
| Prochlorococcus_marinus_MIT_9211 |
| Prochlorococcus_marinus_MIT_9215 |
| Prochlorococcus_marinus_MIT_9301 |
| Prochlorococcus_marinus_MIT_9303 |
| Prochlorococcus_marinus_MIT_9312 |
| Prochlorococcus_marinus_MIT_9313 |
| Prochlorococcus_marinus_MIT_9515 |
| Prochlorococcus_marinus_NATL1A |
| Prochlorococcus_marinus_NATL2A |
| Prochlorococcus_marinus_pastoris_CCMP1986 |
| Propionibacterium_acnes_KPA171202 |
| Prosthecochloris_aestuarii_DSM_271 |
| Prosthecochloris_vibrioformis_DSM_265 |
| Proteus_mirabilis |
| Pseudoalteromonas_atlantica_T6c |
| Pseudoalteromonas_haloplanktis_TAC125 |
| Pseudomonas_aeruginosa_LESB58 |
| Pseudomonas_aeruginosa_PA7 |
| Pseudomonas_aeruginosa_PAO1 |
| Pseudomonas_aeruginosa_UCBPP_PA14 |
| Pseudomonas_fluorescens_Pf_5 |
| Pseudomonas_fluorescens_Pf0_1 |
| Pseudomonas_mendocina_ymp |
| Pseudomonas_putida_F1 |
| Pseudomonas_putida_GB_1 |
| Pseudomonas_putida_KT2440 |
| Pseudomonas_putida_W619 |
| Pseudomonas_stutzeri_A1501 |
| Pseudomonas_syringae_phaseolicola_1448A |
| Pseudomonas_syringae_pv_B728a |
| Pseudomonas_syringae_tomato_DC3000 |
| Psychrobacter_arcticus_273_4 |
| Psychromonas_ingrahamii_37 |
| Pyrobaculum_aerophilum |
| Pyrobaculum_arsenaticum_DSM_13514 |
| Pyrobaculum_calidifontis_JCM_11548 |
| Pyrobaculum_islandicum_DSM_4184 |
| Pyrococcus_furiosus_DSM_3638 |
| Pyrococcus_horikoshii |
| Ralstonia_pickettii_12J |
| Ralstonia_solanacearum_GMI1000 |
| Renibacterium_salmoninarum_ATCC_33209 |
| Rhizobium_etli_CIAT_652 |
| Rhodobacter_sphaeroides_ATCC_17025 |
| Rhodococcus_jostii_RHA1 |
| Rhodopseudomonas_palustris_BisA53 |
| Rhodopseudomonas_palustris_BisB18 |
| Rhodopseudomonas_palustris_BisB5 |
| Rhodopseudomonas_palustris_CGA009 |
| Rhodopseudomonas_palustris_HaA2 |
| Rhodopseudomonas_palustris_TIE_1 |
| Rhodospirillum_centenum_SW |
| Rickettsia_akari_Hartford |
| Rickettsia_bellii_OSU_85_389 |
| Rickettsia_bellii_RML369-C |
| Rickettsia_canadensis_McKiel |
| Rickettsia_conorii_Malish_7 |
| Rickettsia_felis_URRWXCal2 |
| Rickettsia_prowazekii |
| Rickettsia_rickettsii_Iowa |
| Rickettsia_rickettsii_Sheila_Smith |
| Rickettsia_typhi_Wilmington |
| Roseiflexus_castenholzii_DSM_13941 |
| Roseiflexus_RS_1 |
| Roseobacter_denitrificans_OCh_114 |
| Rubrobacter_xylanophilus_DSM_9941 |
| Ruegeria_pomeroyi_DSS_3 |
| Ruegeria_TM1040 |
| Saccharophagus_degradans_2-40 |
| Saccharopolyspora_erythraea_NRRL_2338 |
| Salinibacter_ruber_DSM_13855 |
| Salinispora_arenicola_CNS_205 |
| Salinispora_tropica_CNB-440 |
| Salmonella_enterica_arizonae_serovar_62_z4_z23__ |
| Salmonella_enterica_serovar_Enteritidis_P125109 |
| Salmonella_enterica_serovar_Gallinarum_287_91 |
| Salmonella_enterica_serovar_Paratyphi_A_AKU_12601 |
| Salmonella_enterica_serovar_Paratyphi_A_ATCC_9150 |
| Salmonella_enterica_serovar_Paratyphi_B_SPB7 |
| Salmonella_enterica_serovar_Typhi_Ty2 |
| Salmonella_typhi |
| Salmonella_typhimurium_LT2 |
| Shewanella_amazonensis_SB2B |
| Shewanella_baltica_OS195 |
| Shewanella_baltica_OS223 |
| Shewanella_denitrificans_OS217 |
| Shewanella_frigidimarina_NCIMB_400 |
| Shewanella_halifaxensis_HAW_EB4 |
| Shewanella_loihica_PV_4 |
| Shewanella_MR_4 |
| Shewanella_oneidensis_MR_1 |
| Shewanella_pealeana_ATCC_700345 |
| Shewanella_piezotolerans_WP3 |
| Shewanella_putrefaciens_CN-32 |
| Shewanella_sediminis_HAW_EB3 |
| Shewanella_W3-18-1 |
| Shewanella_woodyi_ATCC_51908 |
| Shigella_dysenteriae_Sd197 |
| Shigella_flexneri_2a |
| Shigella_flexneri_2a_2457T |
| Shigella_flexneri_5_8401 |
| Shigella_sonnei_Ss046 |
| Sodalis_glossinidius__morsitans_ |
| Solibacter_usitatus_Ellin6076 |
| Sorangium_cellulosum__So_ce_56_ |
| Staphylococcus_aureus_COL |
| Staphylococcus_aureus_Mu3 |
| Staphylococcus_aureus_Mu50 |
| Staphylococcus_aureus_MW2 |
| Staphylococcus_aureus_N315 |
| Staphylococcus_aureus_NCTC_8325 |
| Staphylococcus_aureus_Newman |
| Staphylococcus_aureus_RF122 |
| Staphylococcus_epidermidis_ATCC_12228 |
| Staphylococcus_epidermidis_RP62A |
| Staphylococcus_haemolyticus |
| Staphylococcus_saprophyticus_ATCC_15305 |
| Stenotrophomonas_maltophilia_K279a |
| Stenotrophomonas_maltophilia_R551_3 |
| Streptococcus_agalactiae_2603V_R |
| Streptococcus_agalactiae_A909 |
| Streptococcus_agalactiae_NEM316 |
| Streptococcus_equi_zooepidemicus_MGCS10565 |
| Streptococcus_gordonii_Challis_substr_CH1 |
| Streptococcus_mutans_UA159 |
| Streptococcus_pneumoniae_CGSP14 |
| Streptococcus_pneumoniae_D39 |
| Streptococcus_pneumoniae_G54 |
| Streptococcus_pneumoniae_Hungary19A_6 |
| Streptococcus_pneumoniae_R6 |
| Streptococcus_pneumoniae_TIGR4 |
| Streptococcus_pyogenes_M1_GAS |
| Streptococcus_pyogenes_MGAS10270 |
| Streptococcus_pyogenes_MGAS10394 |
| Streptococcus_pyogenes_MGAS10750 |
| Streptococcus_pyogenes_MGAS315 |
| Streptococcus_pyogenes_MGAS5005 |
| Streptococcus_pyogenes_MGAS8232 |
| Streptococcus_pyogenes_NZ131 |
| Streptococcus_sanguinis_SK36 |
| Streptococcus_suis_05ZYH33 |
| Streptococcus_suis_98HAH33 |
| Streptococcus_thermophilus_CNRZ1066 |
| Streptococcus_thermophilus_LMG_18311 |
| Streptomyces_avermitilis_MA_4680 |
| Streptomyces_coelicolor_A3_2_ |
| Streptomyces_griseus_NBRC_13350 |
| Sulfolobus_acidocaldarius_DSM_639 |
| Sulfolobus_solfataricus_P2 |
| Sulfolobus_tokodaii |
| Sulfurihydrogenibium_YO3AOP1 |
| Sulfurovum_NBC37_1 |
| Symbiobacterium_thermophilum_IAM14863 |
| Synechococcus_CC9311 |
| Synechococcus_CC9605 |
| Synechococcus_CC9902 |
| Synechococcus_elongatus_PCC_6301 |
| Synechococcus_JA_3_3Ab |
| Synechococcus_RCC307 |
| Synechococcus_sp_WH8102 |
| Synechococcus_WH_7803 |
| Synechocystis_PCC6803 |
| Syntrophobacter_fumaroxidans_MPOB |
| Syntrophomonas_wolfei_Goettingen |
| Syntrophus_aciditrophicus_SB |
| Thermoanaerobacter_pseudethanolicus_ATCC_33223 |
| Thermoanaerobacter_tengcongensis |
| Thermoanaerobacter_X514 |
| Thermobifida_fusca_YX |
| Thermococcus_kodakaraensis_KOD1 |
| Thermococcus_onnurineus_NA1 |
| Thermodesulfovibrio_yellowstonii_DSM_11347 |
| Thermoplasma_acidophilum |
| Thermoplasma_volcanium |
| Thermoproteus_neutrophilus_V24Sta |
| Thermosipho_africanus_TCF52B |
| Thermosipho_melanesiensis_BI429 |
| Thermosynechococcus_elongatus_BP_1 |
| Thermotoga_lettingae_TMO |
| Thermotoga_maritima |
| Thermotoga_petrophila_RKU-1 |
| Thermotoga_RQ2 |
| Thermus_thermophilus_HB27 |
| Thermus_thermophilus_HB8 |
| Thiobacillus_denitrificans_ATCC_25259 |
| Thiomicrospira_crunogena_XCL_2 |
| Thiomicrospira_denitrificans_ATCC_33889 |
| Treponema_denticola_ATCC_35405 |
| Treponema_pallidum_Nichols |
| Treponema_pallidum_SS14 |
| Trichodesmium_erythraeum_IMS101 |
| Tropheryma_whipplei_TW08_27 |
| Tropheryma_whipplei_Twist |
| uncultured_methanogenic_archaeon_RC-I |
| uncultured_Termite_group_1_bacterium_phylotype_Rs_D17 |
| Ureaplasma_parvum_serovar_3_ATCC_27815 |
| Ureaplasma_parvum_serovar_3_ATCC_700970 |
| Ureaplasma_urealyticum_serovar_10_ATCC_33699 |
| Vibrio_cholerae |
| Vibrio_fischeri_ES114 |
| Vibrio_parahaemolyticus_RIMD_2210633 |
| Vibrio_splendidus_LGP32 |
| Vibrio_vulnificus_CMCP6 |
| Wolbachia_endosymbiont_of_Culex_quinquefasciatus_Pel |
| Wolbachia_endosymbiont_of_Drosophila_melanogaster |
| Wolbachia_endosymbiont_TRS_of_Brugia_malayi |
| Wolinella_succinogenes |
| Xanthomonas_axonopodis_citri_306 |
| Xanthomonas_campestris_8004 |
| Xanthomonas_campestris_ATCC_33913 |
| Xanthomonas_campestris_B100 |
| Xanthomonas_oryzae_KACC10331 |
| Xanthomonas_oryzae_MAFF_311018 |
| Xanthomonas_oryzae_PXO99A |
| Xylella_fastidiosa |
| Xylella_fastidiosa_M12 |
| Xylella_fastidiosa_M23 |
| Yersinia_pestis_biovar_Microtus_91001 |
| Yersinia_pestis_KIM_10 |
| Yersinia_pseudotuberculosis_PB1_ |
| Yersinia_pseudotuberculosis_YPIII |
| Zymomonas_mobilis_ZM4 |
