# Supplementary material for: A Semi-Quantitative, Synteny-Based Method to Improve Functional Predictions for Hypothetical and Poorly Annotated Bacterial and Archaeal Genes
Source: PLoS Comput Biol. 2011 Oct 20;7(10):e1002230. doi: 10.1371/journal.pcbi.1002230 (PMC3197636; doi:10.1371/journal.pcbi.1002230)
Supplement: Table S8 — List of organisms used in pairwise comparisons of percentage of syntenous genes that are functionally related and GOC. Genomes were downloaded from the STRING database. (DOC) [file pcbi.1002230.s011.doc]

**Table S8: Method validation with well-characterized genomes. The query species’ genes were annotated via the synteny-based method with the annotations of its ortholog in the subject species. White indicates correct annotations. Red indicates incorrect annotations. Gray indicates ambiguous cases. Bold indicates gene with shared function between the two comparisons. We only included the shared genes from the E. coli T. maritima comparison.**

| **Query species** | **Subject species** | **Query gene** | **Subject gene** | **Original annotation** | **Synteny-based annotation** |
| --- | --- | --- | --- | --- | --- |
| Sulfolobus solfataricus P2 | Haloferax volcanii | NP_341639 | YP_003536791 | rpoN DNA-directed RNA polymerase subunit N (EC:2.7.7.6) K03058 DNA-directed RNA polymerase subunit N [EC:2.7.7.6] (db=KEGG evalue=6.0e-32 bit_score=139.0 identity=100.0 coverage=98.4848484848485) (BLAST) | rpoN; DNA-directed RNA polymerase subunit N (EC:2.7.7.6); K03058 DNA-directed RNA polymerase subunit N [EC:2.7.7.6] (db=KEGG evalue=2.0e-30 bit_score=134.0 identity=100.0 coverage=98.4375) (BLAST) |
| Sulfolobus solfataricus P2 | Haloferax volcanii | NP_341642 | YP_003536794 | rpl18E 50S ribosomal protein L18e K02883 large subunit ribosomal protein L18e (db=KEGG evalue=1.0e-62 bit_score=241.0 identity=100.0 coverage=99.1596638655462) (BLAST) | rpl18R; ribosomal protein L18.eR; K02883 large subunit ribosomal protein L18e (db=KEGG evalue=2.0e-60 bit_score=233.0 identity=100.0 coverage=99.1379310344828) (BLAST) |
| Sulfolobus solfataricus P2 | Haloferax volcanii | NP_341643 | YP_003536795 | sso:SSO0071 rpoD DNA-directed RNA polymerase, subunit D (RpoD) (EC:2.7.7.6) K03047 DNA-directed RNA polymerase subunit D [EC:2.7.7.6] (db=KEGG) (RBH) | hvo:HVO_2781 rpoD; DNA-directed RNA polymerase subunit D (EC:2.7.7.6); K03047 DNA-directed RNA polymerase subunit D [EC:2.7.7.6] (db=KEGG) (RBH) |
| Sulfolobus solfataricus P2 | Haloferax volcanii | NP_341645 | YP_003536797 | sso:SSO0073 rps4p 30S ribosomal protein S4P K02986 small subunit ribosomal protein S4 (db=KEGG) (RBH) | hvo:HVO_2783 rps4; ribosomal protein S4; K02986 small subunit ribosomal protein S4 (db=KEGG) (RBH) |
| Sulfolobus solfataricus P2 | Haloferax volcanii | NP_341667 | YP_003536952 | sso:SSO0101 pheT phenylalanyl-tRNA synthetase subunit beta (EC:6.1.1.20) K01890 phenylalanyl-tRNA synthetase beta chain [EC:6.1.1.20] (db=KEGG) (RBH) | hvo:HVO_2947 pheT; phenylalanyl-tRNA synthetase beta subunit (EC:6.1.1.20); K01890 phenylalanyl-tRNA synthetase beta chain [EC:6.1.1.20] (db=KEGG) (RBH) |
| Sulfolobus solfataricus P2 | Haloferax volcanii | NP_341711 | YP_003534119 | sso:SSO0155 argC N-acetyl-gamma-glutamyl-phosphate reductase (EC:1.2.1.38) K00145 N-acetyl-gamma-glutamyl-phosphate/N-acetyl-gamma-aminoadipyl-phosphate reductase [EC:1.2.1.38 1.2.1.-] (db=KEGG) (RBH) | hvo:HVO_0045 argC; N-acetyl-gamma-glutamyl-phosphate reductase (EC:1.2.1.38); K00145 N-acetyl-gamma-glutamyl-phosphate/N-acetyl-gamma-aminoadipyl-phosphate reductase [EC:1.2.1.38 1.2.1.-] (db=KEGG) (RBH) |
| Sulfolobus solfataricus P2 | Haloferax volcanii | NP_341712 | YP_003534118 | sso:SSO0156 argB acetylglutamate/acetylaminoadipate kinase (EC:2.7.2.8) K00930 acetylglutamate/acetylaminoadipate kinase [EC:2.7.2.8 2.7.2.-] (db=KEGG) (RBH) | hvo:HVO_0044 argB; acetylglutamate kinase (EC:2.7.2.8); K00930 acetylglutamate/acetylaminoadipate kinase [EC:2.7.2.8 2.7.2.-] (db=KEGG) (RBH) |
| Sulfolobus solfataricus P2 | Haloferax volcanii | NP_341716 | YP_003534117 | sso:SSO0160 argD acetylornithine aminotransferase ArgD (EC:2.6.1.11) K05830 acetylornithine/acetyl-lysine aminotransferase [EC:2.6.1.11 2.6.1.-] (db=KEGG) (RBH) | hvo:HVO_0043 argD; acetylornithine aminotransferase (EC:2.6.1.11); K00821 acetylornithine/N-succinyldiaminopimelate aminotransferase [EC:2.6.1.11 2.6.1.17] (db=KEGG) (RBH) |
| Sulfolobus solfataricus P2 | Haloferax volcanii | NP_341717 | YP_003534116 | sso:SSO0162 acetyl-lysine deacetylase K05831 acetyl-lysine deacetylase [EC:3.5.1.-] (db=KEGG) (RBH) | hvo:HVO_0042 argE; glutamate carboxypeptidase (EC:3.5.1.-); K05831 acetyl-lysine deacetylase [EC:3.5.1.-] (db=KEGG) (RBH) |
| Sulfolobus solfataricus P2 | Haloferax volcanii | NP_341731 | YP_003534720 | peptidyl-tRNA hydrolase (EC:3.1.1.29) K04794 peptidyl-tRNA hydrolase, PTH2 family [EC:3.1.1.29] (db=KEGG evalue=1.0e-62 bit_score=241.0 identity=100.0 coverage=99.1666666666667) (BLAST) | conserved hypothetical protein TIGR00283; K04794 peptidyl-tRNA hydrolase, PTH2 family [EC:3.1.1.29] (db=KEGG evalue=6.0e-58 bit_score=225.0 identity=100.0 coverage=99.1071428571429) (BLAST) |
| Sulfolobus solfataricus P2 | Haloferax volcanii | NP_341738 | YP_003534155 | sso:SSO0182 hemL glutamate-1-semialdehyde aminotransferase (EC:5.4.3.8) K01845 glutamate-1-semialdehyde 2,1-aminomutase [EC:5.4.3.8] (db=KEGG) (RBH) | hvo:HVO_0081 hemL; glutamate-1-semialdehyde 2,1-aminomutase (EC:5.4.3.8); K01845 glutamate-1-semialdehyde 2,1-aminomutase [EC:5.4.3.8] (db=KEGG) (RBH) |
| Sulfolobus solfataricus P2 | Haloferax volcanii | NP_341739 | YP_003534152 | sso:SSO0183 hemC porphobilinogen deaminase K01749 hydroxymethylbilane synthase [EC:2.5.1.61] (db=KEGG) (RBH) | hvo:HVO_0078 hemC; porphobilinogen deaminase (EC:2.5.1.61); K01749 hydroxymethylbilane synthase [EC:2.5.1.61] (db=KEGG) (RBH) |
| Sulfolobus solfataricus P2 | Haloferax volcanii | NP_341772 | YP_003534428 | rps12P 30S ribosomal protein S12P K02950 small subunit ribosomal protein S12 (db=KEGG evalue=2.0e-60 bit_score=234.0 identity=100.0 coverage=99.3333333333333) (BLAST) | rps12; ribosomal protein S12; K02950 small subunit ribosomal protein S12 (db=KEGG evalue=2.0e-76 bit_score=286.0 identity=100.0 coverage=99.2957746478873) (BLAST) |
| Sulfolobus solfataricus P2 | Haloferax volcanii | NP_341781 | YP_003536755 | ndk nucleoside diphosphate kinase (EC:2.7.4.6) K00940 nucleoside-diphosphate kinase [EC:2.7.4.6] (db=KEGG evalue=9.0e-75 bit_score=281.0 identity=100.0 coverage=99.2753623188406) (BLAST) | hvo:HVO_2740 ndk; nucleoside diphosphate kinase (EC:2.7.4.6); K00940 nucleoside-diphosphate kinase [EC:2.7.4.6] (db=KEGG) (RBH) |
| Sulfolobus solfataricus P2 | Haloferax volcanii | NP_341783 | YP_003536753 | rps28e 30S ribosomal protein S28e K02979 small subunit ribosomal protein S28e (db=KEGG evalue=2.0e-47 bit_score=191.0 identity=100.0 coverage=98.9795918367347) (BLAST) | rps28R; ribosomal protein S28.eR; K02979 small subunit ribosomal protein S28e (db=KEGG evalue=6.0e-35 bit_score=149.0 identity=100.0 coverage=98.6486486486486) (BLAST) |
| Sulfolobus solfataricus P2 | Haloferax volcanii | NP_341803 | YP_003534841 | sso:SSO0254 hypothetical protein K09134 hypothetical protein (db=KEGG) (RBH) | hvo:HVO_0781 hypothetical protein; K09134 hypothetical protein (db=KEGG) (RBH) |
| Sulfolobus solfataricus P2 | Haloferax volcanii | NP_341804 | YP_003534842 | nicotinamide mononucleotide adenylyltransferase, putative (EC:2.7.7.1) K00952 nicotinamide-nucleotide adenylyltransferase [EC:2.7.7.1] (db=KEGG evalue=2.0e-61 bit_score=237.0 identity=100.0 coverage=99.1452991452991) (BLAST) | hvo:HVO_0782 nicotinamide-nucleotide adenylyltransferase (EC:2.7.7.1); K00952 nicotinamide-nucleotide adenylyltransferase [EC:2.7.7.1] (db=KEGG) (RBH) |
| Sulfolobus solfataricus P2 | Haloferax volcanii | NP_341815 | YP_003535225 | sso:SSO0266 tfe transcription factor E K03136 transcription initiation factor TFIIE alpha subunit (db=KEGG) (RBH) | hvo:HVO_1174 TFIIE alpha subunit; K03136 transcription initiation factor TFIIE alpha subunit (db=KEGG) (RBH) |
| Sulfolobus solfataricus P2 | Haloferax volcanii | NP_341816 | YP_003535224 | sso:SSO0267 hypothetical protein K07254 hypothetical protein (db=KEGG) (RBH) | hvo:HVO_1173 SpoU-like RNA methylase; K07254 hypothetical protein (db=KEGG) (RBH) |
| Sulfolobus solfataricus P2 | Haloferax volcanii | NP_341870 | YP_003535038 | sso:SSO0325 nuoH NADH dehydrogenase subunit H (EC:1.6.5.3) K00337 NADH dehydrogenase I subunit H [EC:1.6.5.3] (db=KEGG) (RBH) | hvo:HVO_0981 nuoH; NADH dehydrogenase-like complex subunit H (EC:1.6.5.-); K00337 NADH dehydrogenase I subunit H [EC:1.6.5.3] (db=KEGG) (RBH) |
| Sulfolobus solfataricus P2 | Haloferax volcanii | NP_341871 | YP_003535039 | sso:SSO0326 nuoI NADH dehydrogenase subunit I (EC:1.6.5.3) K00338 NADH dehydrogenase I subunit I [EC:1.6.5.3] (db=KEGG) (RBH) | hvo:HVO_0982 nuoI; NADH dehydrogenase-like complex subunit I (EC:1.6.5.-); K00338 NADH dehydrogenase I subunit I [EC:1.6.5.3] (db=KEGG) (RBH) |
| Sulfolobus solfataricus P2 | Haloferax volcanii | NP_341883 | YP_003536771 | sso:SSO0344 rplP0 acidic ribosomal protein P0 K02864 large subunit ribosomal protein L10 (db=KEGG) (RBH) | hvo:HVO_2756 rpl10; ribosomal protein L10; K02864 large subunit ribosomal protein L10 (db=KEGG) (RBH) |
| Sulfolobus solfataricus P2 | Haloferax volcanii | NP_341945 | YP_003536758 | sso:SSO0406 metE-1 5-methyltetrahydropteroyltriglutamate--homocysteine methyltransferase (EC:2.1.1.14) K00549 5-methyltetrahydropteroyltriglutamate--homocysteine methyltransferase [EC:2.1.1.14] (db=KEGG) (RBH) | hvo:HVO_2743 methionine synthase vitamin-B12 independent; K00549 5-methyltetrahydropteroyltriglutamate--homocysteine methyltransferase [EC:2.1.1.14] (db=KEGG) (RBH) |
| Sulfolobus solfataricus P2 | Haloferax volcanii | NP_341946 | YP_003536757 | sso:SSO0407 metE-2 methionine synthase (EC:2.1.1.14) K00549 5-methyltetrahydropteroyltriglutamate--homocysteine methyltransferase [EC:2.1.1.14] (db=KEGG) (RBH) | hvo:HVO_2742 metE; 5-methyltetrahydropteroyltriglutamate-homocysteine methyltransferase (EC:2.1.1.-); K00549 5-methyltetrahydropteroyltriglutamate--homocysteine methyltransferase [EC:2.1.1.14] (db=KEGG) (RBH) |
| Sulfolobus solfataricus P2 | Haloferax volcanii | NP_341950 | YP_003535931 | sso:SSO0412 eif2G translation initiation factor IF-2 subunit gamma K03242 translation initiation factor eIF-2 gamma subunit (db=KEGG) (RBH) | hvo:HVO_1901 tif2g; translation initiation factor aIF-2 gamma subunit; K03242 translation initiation factor eIF-2 gamma subunit (db=KEGG) (RBH) |
| Sulfolobus solfataricus P2 | Haloferax volcanii | NP_341952 | YP_003535929 | sso:SSO0415 rpoE1 DNA-directed RNA polymerase subunit E' (EC:2.7.7.6) K03049 DNA-directed RNA polymerase subunit E' [EC:2.7.7.6] (db=KEGG) (RBH) | rpoE1; DNA-directed RNA polymerase subunit E (EC:2.7.7.6); K03049 DNA-directed RNA polymerase subunit E' [EC:2.7.7.6] (db=KEGG evalue=1.0e-79 bit_score=298.0 identity=100.0 coverage=99.4736842105263) (BLAST) |
| Sulfolobus solfataricus P2 | Haloferax volcanii | NP_341954 | YP_003535927 | sso:SSO0416 hypothetical protein K09735 hypothetical protein (db=KEGG) (RBH) | hypothetical protein; K09735 hypothetical protein (db=KEGG evalue=1.0e-66 bit_score=254.0 identity=100.0 coverage=99.4413407821229) (BLAST) |
| Sulfolobus solfataricus P2 | Haloferax volcanii | NP_342006 | YP_003535645 | sis:LS215_1758 type III restriction protein res subunit (db=KEGG) (RBH) | hvo:HVO_1598 rad25c; DNA repair helicase RAD25 (EC:3.6.1.-) (db=KEGG) (RBH) |
| Sulfolobus solfataricus P2 | Haloferax volcanii | NP_342007 | YP_003535643 | siy:YG5714_1721 protein of unknown function DUF790 K09744 hypothetical protein (db=KEGG) (RBH) | hvo:HVO_1596 protein of unknown function (DUF790) superfamily; K09744 hypothetical protein (db=KEGG) (RBH) |
| Sulfolobus solfataricus P2 | Haloferax volcanii | NP_342019 | YP_003536402 | siy:YG5714_1709 phosphate ABC transporter ATP-binding protein K02036 phosphate transport system ATP-binding protein [EC:3.6.3.27] (db=KEGG) (RBH) | hvo:HVO_2378 pstB1; ABC-type transport system ATP-binding protein (probable substrate phosphate) (EC:3.6.3.27); K02036 phosphate transport system ATP-binding protein [EC:3.6.3.27] (db=KEGG) (RBH) |
| Sulfolobus solfataricus P2 | Haloferax volcanii | NP_342021 | YP_003536400 | sso:SSO0490 pstC phosphate ABC transporter permease K02037 phosphate transport system permease protein (db=KEGG) (RBH) | hvo:HVO_2376 pstC1; ABC-type transport system permease protein (probable substrate phosphate); K02037 phosphate transport system permease protein (db=KEGG) (RBH) |
| Sulfolobus solfataricus P2 | Haloferax volcanii | NP_342057 | YP_003534553 | sso:SSO0527 pgk phosphoglycerate kinase (EC:2.7.2.3) K00927 phosphoglycerate kinase [EC:2.7.2.3] (db=KEGG) (RBH) | hvo:HVO_0480 pgk; phosphoglycerate kinase (EC:2.7.2.3); K00927 phosphoglycerate kinase [EC:2.7.2.3] (db=KEGG) (RBH) |
| Sulfolobus solfataricus P2 | Haloferax volcanii | NP_342058 | YP_003534551 | sso:SSO0528 gap glyceraldehyde-3-phosphate dehydrogenase (EC:1.2.1.59) K00150 glyceraldehyde-3-phosphate dehydrogenase (NAD(P)) [EC:1.2.1.59] (db=KEGG) (RBH) | hvo:HVO_0478 glyceraldehyde-3-phosphate dehydrogenase, type II (EC:1.2.1.59); K00150 glyceraldehyde-3-phosphate dehydrogenase (NAD(P)) [EC:1.2.1.59] (db=KEGG) (RBH) |
| Sulfolobus solfataricus P2 | Haloferax volcanii | NP_342089 | YP_003534393 | sso:SSO0563 atpA V-type ATP synthase subunit A (EC:3.6.3.14) K02117 V-type H+-transporting ATPase subunit A [EC:3.6.3.14] (db=KEGG) (RBH) | hvo:HVO_0316 atpA; A-type ATP synthase subunit A (EC:3.6.3.14); K02117 V-type H+-transporting ATPase subunit A [EC:3.6.3.14] (db=KEGG) (RBH) |
| Sulfolobus solfataricus P2 | Haloferax volcanii | NP_342090 | YP_003534394 | sso:SSO0564 atpB V-type ATP synthase subunit B (EC:3.6.3.14) K02118 V-type H+-transporting ATPase subunit B [EC:3.6.3.14] (db=KEGG) (RBH) | hvo:HVO_0317 atpB; A-type ATP synthase subunit B (EC:3.6.3.14); K02118 V-type H+-transporting ATPase subunit B [EC:3.6.3.14] (db=KEGG) (RBH) |
| Sulfolobus solfataricus P2 | Haloferax volcanii | NP_342100 | YP_003535555 | sso:SSO0576 ilvC-1 ketol-acid reductoisomerase (EC:1.1.1.86) K00053 ketol-acid reductoisomerase [EC:1.1.1.86] (db=KEGG) (RBH) | hvo:HVO_1506 ilvC; ketol-acid reductoisomerase (EC:1.1.1.86); K00053 ketol-acid reductoisomerase [EC:1.1.1.86] (db=KEGG) (RBH) |
| Sulfolobus solfataricus P2 | Haloferax volcanii | NP_342102 | YP_003535557 | sso:SSO0579 ilvB-2 acetolactate synthase catalytic subunit (EC:2.2.1.6) K01652 acetolactate synthase I/II/III large subunit [EC:2.2.1.6] (db=KEGG) (RBH) | hvo:HVO_1508 ilvB1; acetolactate synthase large subunit (EC:2.2.1.6); K01652 acetolactate synthase I/II/III large subunit [EC:2.2.1.6] (db=KEGG) (RBH) |
| Sulfolobus solfataricus P2 | Haloferax volcanii | NP_342116 | YP_003536993 | sso:SSO0594 hisA 1-(5-phosphoribosyl)-5-[(5-phosphoribosylamino)methylideneamino] imidazole-4-carboxamide isomerase (EC:5.3.1.16) K01814 phosphoribosylformimino-5-aminoimidazole carboxamide ribotide isomerase [EC:5.3.1.16] (db=KEGG) (RBH) | hvo:HVO_2988 hisA; phosphoribosylformimino-5-aminoimidazole carboxamide ribotide isomerase (EC:5.3.1.16); K01814 phosphoribosylformimino-5-aminoimidazole carboxamide ribotide isomerase [EC:5.3.1.16] (db=KEGG) (RBH) |
| Sulfolobus solfataricus P2 | Haloferax volcanii | NP_342117 | YP_003536991 | sso:SSO0596 hisB imidazoleglycerol-phosphate dehydratase (EC:4.2.1.19) K01693 imidazoleglycerol-phosphate dehydratase [EC:4.2.1.19] (db=KEGG) (RBH) | hvo:HVO_2986 hisB; imidazoleglycerol-phosphate dehydratase (EC:4.2.1.19); K01693 imidazoleglycerol-phosphate dehydratase [EC:4.2.1.19] (db=KEGG) (RBH) |
| Sulfolobus solfataricus P2 | Haloferax volcanii | NP_342134 | YP_003535504 | sso:SSO0613 pyrI aspartate carbamoyltransferase regulatory subunit (EC:2.1.3.2) K00610 aspartate carbamoyltransferase regulatory subunit (db=KEGG) (RBH) | hvo:HVO_1455 pyrI; aspartate carbamoyltransferase, regulatory subunit; K00610 aspartate carbamoyltransferase regulatory subunit (db=KEGG) (RBH) |
| Sulfolobus solfataricus P2 | Haloferax volcanii | NP_342135 | YP_003535503 | sso:SSO0614 pyrB aspartate carbamoyltransferase catalytic subunit (EC:2.1.3.2) K00609 aspartate carbamoyltransferase catalytic subunit [EC:2.1.3.2] (db=KEGG) (RBH) | hvo:HVO_1454 pyrB; aspartate carbamoyltransferase (EC:2.1.3.2); K00609 aspartate carbamoyltransferase catalytic subunit [EC:2.1.3.2] (db=KEGG) (RBH) |
| Sulfolobus solfataricus P2 | Haloferax volcanii | NP_342156 | YP_003534123 | sso:SSO0638 argG argininosuccinate synthase (EC:6.3.4.5) K01940 argininosuccinate synthase [EC:6.3.4.5] (db=KEGG) (RBH) | hvo:HVO_0049 argG; argininosuccinate synthase (EC:6.3.4.5); K01940 argininosuccinate synthase [EC:6.3.4.5] (db=KEGG) (RBH) |
| Sulfolobus solfataricus P2 | Haloferax volcanii | NP_342157 | YP_003534122 | sso:SSO0639 argH argininosuccinate lyase (EC:4.3.2.1) K01755 argininosuccinate lyase [EC:4.3.2.1] (db=KEGG) (RBH) | hvo:HVO_0048 argH; argininosuccinate lyase (EC:4.3.2.1); K01755 argininosuccinate lyase [EC:4.3.2.1] (db=KEGG) (RBH) |
| Sulfolobus solfataricus P2 | Haloferax volcanii | NP_342208 | YP_003536561 | sso:SSO0697 rpl30p 50S ribosomal protein L30P K02907 large subunit ribosomal protein L30 (db=KEGG) (RBH) | hvo:HVO_2543 rpl30; ribosomal protein L30; K02907 large subunit ribosomal protein L30 (db=KEGG) (RBH) |
| Sulfolobus solfataricus P2 | Haloferax volcanii | NP_342212 | YP_003536565 | rpl32e 50S ribosomal protein L32e K02912 large subunit ribosomal protein L32e (db=KEGG evalue=5.0e-73 bit_score=275.0 identity=100.0 coverage=99.2753623188406) (BLAST) | hvo:HVO_2547 rpl32R; ribosomal protein L32.eR; K02912 large subunit ribosomal protein L32e (db=KEGG) (RBH) |
| Sulfolobus solfataricus P2 | Haloferax volcanii | NP_342217 | YP_003536570 | sso:SSO0705 rps4E 30S ribosomal protein S4e K02987 small subunit ribosomal protein S4e (db=KEGG) (RBH) | hvo:HVO_2552 rps4R; ribosomal protein S4.eR; K02987 small subunit ribosomal protein S4e (db=KEGG) (RBH) |
| Sulfolobus solfataricus P2 | Haloferax volcanii | NP_342218 | YP_003536571 | rpl24p 50S ribosomal protein L24P K02895 large subunit ribosomal protein L24 (db=KEGG evalue=1.0e-60 bit_score=234.0 identity=100.0 coverage=99.1596638655462) (BLAST) | rpl24; ribosomal protein L24; K02895 large subunit ribosomal protein L24 (db=KEGG evalue=8.0e-60 bit_score=231.0 identity=100.0 coverage=99.1525423728814) (BLAST) |
| Sulfolobus solfataricus P2 | Haloferax volcanii | NP_342227 | YP_003536581 | sso:SSO0718 rpl4lp 50S ribosomal protein L4P K02930 large subunit ribosomal protein L4e (db=KEGG) (RBH) | hvo:HVO_2563 rpl4R; ribosomal protein L4.eR; K02930 large subunit ribosomal protein L4e (db=KEGG) (RBH) |
| Sulfolobus solfataricus P2 | Haloferax volcanii | NP_342255 | YP_003536762 | sso:SSO0750 hypothetical protein K07572 putative nucleotide binding protein (db=KEGG) (RBH) | hvo:HVO_2747 predicted RNA-binding protein; K07572 putative nucleotide binding protein (db=KEGG) (RBH) |
| Sulfolobus solfataricus P2 | Haloferax volcanii | NP_342257 | YP_003536764 | rpl21E 50S ribosomal protein L21e K02889 large subunit ribosomal protein L21e (db=KEGG evalue=2.0e-42 bit_score=174.0 identity=100.0 coverage=99.009900990099) (BLAST) | rpl21R; ribosomal protein L21.eR; K02889 large subunit ribosomal protein L21e (db=KEGG evalue=8.0e-49 bit_score=195.0 identity=100.0 coverage=98.9583333333333) (BLAST) |
| Sulfolobus solfataricus P2 | Haloferax volcanii | NP_342335 | YP_003536084 | sso:SSO0830 rfbB-1 dTDP-glucose 4,6-dehydratase (RfbB-1) (EC:4.2.1.46) K01710 dTDP-glucose 4,6-dehydratase [EC:4.2.1.46] (db=KEGG) (RBH) | hvo:HVO_2059 galE5; UDP-glucose 4-epimerase (EC:5.1.3.2); K01710 dTDP-glucose 4,6-dehydratase [EC:4.2.1.46] (db=KEGG) (RBH) |
| Sulfolobus solfataricus P2 | Haloferax volcanii | NP_342336 | YP_003536082 | sso:SSO0831 sugar phosphate nucleotydyl transferase (EC:2.7.7.-) K00973 glucose-1-phosphate thymidylyltransferase [EC:2.7.7.24] (db=KEGG) (RBH) | hvo:HVO_2057 graD2; sugar nucleotidyltransferase (EC:2.7.7.-); K00973 glucose-1-phosphate thymidylyltransferase [EC:2.7.7.24] (db=KEGG) (RBH) |
| Sulfolobus solfataricus P2 | Haloferax volcanii | NP_342337 | YP_003536083 | sso:SSO0832 rfbD-1 dTDP-4-dehydrorhamnose reductase (RfbD-1) (EC:1.1.1.133) K00067 dTDP-4-dehydrorhamnose reductase [EC:1.1.1.133] (db=KEGG) (RBH) | hvo:HVO_2058 RmlD substrate binding domain superfamily; K00067 dTDP-4-dehydrorhamnose reductase [EC:1.1.1.133] (db=KEGG) (RBH) |
| Sulfolobus solfataricus P2 | Haloferax volcanii | NP_342383 | YP_003536477 | sso:SSO0890 trpD anthranilate phosphoribosyltransferase (EC:2.4.2.18) K00766 anthranilate phosphoribosyltransferase [EC:2.4.2.18] (db=KEGG) (RBH) | hvo:HVO_2456 trpD1; anthranilate phosphoribosyltransferase (EC:2.4.2.18); K00766 anthranilate phosphoribosyltransferase [EC:2.4.2.18] (db=KEGG) (RBH) |
| Sulfolobus solfataricus P2 | Haloferax volcanii | NP_342385 | YP_003536475 | sso:SSO0893 trpE anthranilate synthase component I K01657 anthranilate synthase component I [EC:4.1.3.27] (db=KEGG) (RBH) | hvo:HVO_2454 trpE; anthranilate synthase component I (EC:4.1.3.27); K01657 anthranilate synthase component I [EC:4.1.3.27] (db=KEGG) (RBH) |
| Sulfolobus solfataricus P2 | Haloferax volcanii | NP_342386 | YP_003536474 | sso:SSO0894 trpGD anthranilate synthase component II (EC:4.1.3.27) K01658 anthranilate synthase component II [EC:4.1.3.27] (db=KEGG) (RBH) | hvo:HVO_2453 trpG; anthranilate synthase component II (EC:4.1.3.27); K01658 anthranilate synthase component II [EC:4.1.3.27] (db=KEGG) (RBH) |
| Sulfolobus solfataricus P2 | Haloferax volcanii | NP_342409 | YP_003536424 | sso:SSO0917 glycine dehydrogenase subunit 2 (EC:1.4.4.2) K00283 glycine dehydrogenase subunit 2 [EC:1.4.4.2] (db=KEGG) (RBH) | hvo:HVO_2401 glycine cleavage system P-protein (EC:1.4.4.2); K00283 glycine dehydrogenase subunit 2 [EC:1.4.4.2] (db=KEGG) (RBH) |
| Sulfolobus solfataricus P2 | Haloferax volcanii | NP_342410 | YP_003536425 | sso:SSO0918 glycine dehydrogenase subunit 1 (EC:1.4.4.2) K00282 glycine dehydrogenase subunit 1 [EC:1.4.4.2] (db=KEGG) (RBH) | hvo:HVO_2402 gcvP; glycine dehydrogenase (decarboxylating) (glycine cleavage system protein P-1) (EC:1.4.4.2); K00282 glycine dehydrogenase subunit 1 [EC:1.4.4.2] (db=KEGG) (RBH) |
| Sulfolobus solfataricus P2 | Haloferax volcanii | NP_342411 | YP_003536427 | sso:SSO0919 gcvT glycine cleavage system aminomethyltransferase T (EC:2.1.2.10) K00605 aminomethyltransferase [EC:2.1.2.10] (db=KEGG) (RBH) | hvo:HVO_2404 gcvT; aminomethyltransferase (glycine cleavage system protein T) (EC:2.1.2.10); K00605 aminomethyltransferase [EC:2.1.2.10] (db=KEGG) (RBH) |
| Sulfolobus solfataricus P2 | Haloferax volcanii | NP_342412 | YP_003536426 | gcvH glycine cleavage system protein H K02437 glycine cleavage system H protein (db=KEGG evalue=1.0e-59 bit_score=231.0 identity=100.0 coverage=99.2700729927007) (BLAST) | gcvH; glycine cleavage system protein H; K02437 glycine cleavage system H protein (db=KEGG evalue=3.0e-65 bit_score=249.0 identity=100.0 coverage=99.2063492063492) (BLAST) |
| Sulfolobus solfataricus P2 | Haloferax volcanii | NP_342425 | YP_003535715 | sso:SSO0939 C/D box methylation guide ribonucleoprotein complex aNOP56 subunit (db=KEGG) (RBH) | hvo:HVO_1670 nop56; archaeal nucleolar protein-like protein (db=KEGG) (RBH) |
| Sulfolobus solfataricus P2 | Haloferax volcanii | NP_342426 | YP_003535714 | sso:SSO0940 fibrillarin K04795 fibrillarin-like pre-rRNA processing protein (db=KEGG) (RBH) | hvo:HVO_1669 fib; fibrillarin-like pre-rRNA processing protein; K04795 fibrillarin-like pre-rRNA processing protein (db=KEGG) (RBH) |
| Sulfolobus solfataricus P2 | Haloferax volcanii | NP_342477 | YP_003536595 | sso:SSO0996 nicotinate-nucleotide pyrophosphorylase (EC:2.4.2.19) K00767 nicotinate-nucleotide pyrophosphorylase (carboxylating) [EC:2.4.2.19] (db=KEGG) (RBH) | hvo:HVO_2579 nadC; nicotinate-nucleotide pyrophosphorylase (carboxylating) (EC:2.4.2.19); K00767 nicotinate-nucleotide pyrophosphorylase (carboxylating) [EC:2.4.2.19] (db=KEGG) (RBH) |
| Sulfolobus solfataricus P2 | Haloferax volcanii | NP_342478 | YP_003536596 | sso:SSO0997 nadB aspartate oxidase (NadB) (EC:1.4.3.16) K00278 L-aspartate oxidase [EC:1.4.3.16] (db=KEGG) (RBH) | hvo:HVO_2580 FAD binding domain, putative; K00278 L-aspartate oxidase [EC:1.4.3.16] (db=KEGG) (RBH) |
| Sulfolobus solfataricus P2 | Haloferax volcanii | NP_342479 | YP_003536597 | sso:SSO0998 nadA quinolinate synthetase K03517 quinolinate synthase [EC:2.5.1.72] (db=KEGG) (RBH) | hvo:HVO_2581 nadA; quinolinate synthetase complex, A subunit; K03517 quinolinate synthase [EC:2.5.1.72] (db=KEGG) (RBH) |
| Sulfolobus solfataricus P2 | Haloferax volcanii | NP_342537 | YP_003535034 | purE phosphoribosylaminoimidazole carboxylase catalytic subunit (PurE) (EC:4.1.1.21) K01588 5-(carboxyamino)imidazole ribonucleotide mutase [EC:5.4.99.18] (db=KEGG evalue=1.0e-77 bit_score=290.0 identity=100.0 coverage=99.3670886075949) (BLAST) | hvo:HVO_0977 purE; phosphoribosylaminoimidazole carboxylase, catalytic subunit (EC:4.1.1.21); K01588 5-(carboxyamino)imidazole ribonucleotide mutase [EC:5.4.99.18] (db=KEGG) (RBH) |
| Sulfolobus solfataricus P2 | Haloferax volcanii | NP_342538 | YP_003535033 | sso:SSO1065 purK phosphoribosylaminoimidazole carboxylase ATPase subunit (EC:4.1.1.21) K01589 5-(carboxyamino)imidazole ribonucleotide synthase [EC:6.3.4.18] (db=KEGG) (RBH) | hvo:HVO_0976 purK; phosphoribosylaminoimidazole carboxylase, ATPase subunit (EC:4.1.1.21); K01589 5-(carboxyamino)imidazole ribonucleotide synthase [EC:6.3.4.18] (db=KEGG) (RBH) |
| Sulfolobus solfataricus P2 | Haloferax volcanii | NP_342626 | YP_003534628 | sso:SSO1168 sugar ABC transporter K02023 multiple sugar transport system ATP-binding protein (db=KEGG) (RBH) | hvo:HVO_0565 malK; ABC-type transport system ATP-binding protein (probable substrate maltose); K10112 maltose/maltodextrin transport system ATP-binding protein (db=KEGG) (RBH) |
| Sulfolobus solfataricus P2 | Haloferax volcanii | NP_342628 | YP_003534626 | sso:SSO1170 sugar transport protein K02025 multiple sugar transport system permease protein (db=KEGG) (RBH) | hvo:HVO_0563 malF; ABC-type transport system permease protein (probable substrate maltose); K10114 maltooligosaccharide transport system permease protein (db=KEGG) (RBH) |
| Sulfolobus solfataricus P2 | Haloferax volcanii | NP_342813 | YP_003536611 | sso:SSO1369 pdhA-1 pyruvate dehydrogenase alpha subunit (lipoamide) (EC:1.2.4.1) K00161 pyruvate dehydrogenase E1 component subunit alpha [EC:1.2.4.1] (db=KEGG) (RBH) | hvo:HVO_2595 oadhA2; 2-oxoacid dehydrogenase E1 component alpha subunit (EC:1.2.4.-); K00161 pyruvate dehydrogenase E1 component subunit alpha [EC:1.2.4.1] (db=KEGG) (RBH) |
| Sulfolobus solfataricus P2 | Haloferax volcanii | NP_342814 | YP_003536612 | sso:SSO1370 pdhB-1 pyruvate dehydrogenase beta subunit (lipoamide) (EC:1.2.4.1) K00162 pyruvate dehydrogenase E1 component subunit beta [EC:1.2.4.1] (db=KEGG) (RBH) | hvo:HVO_2596 oadhB2; 2-oxoacid dehydrogenase E1 component beta subunit (EC:1.2.4.-); K00162 pyruvate dehydrogenase E1 component subunit beta [EC:1.2.4.1] (db=KEGG) (RBH) |
| Sulfolobus solfataricus P2 | Haloferax volcanii | NP_342958 | YP_003534730 | sso:SSO1525 pdhA-2 pyruvate dehydrogenase alpha subunit (lipoamide) (EC:1.2.4.1) K00161 pyruvate dehydrogenase E1 component subunit alpha [EC:1.2.4.1] (db=KEGG) (RBH) | hvo:HVO_0669 oadhA3; 2-oxoacid dehydrogenase E1 component alpha subunit (EC:1.2.4.-); K00161 pyruvate dehydrogenase E1 component subunit alpha [EC:1.2.4.1] (db=KEGG) (RBH) |
| Sulfolobus solfataricus P2 | Haloferax volcanii | NP_342960 | YP_003534728 | sso:SSO1527 acoX acetoin catabolism protein AcoX (db=KEGG) (RBH) | hvo:HVO_0667 ATP-NAD kinase (db=KEGG) (RBH) |
| Sulfolobus solfataricus P2 | Haloferax volcanii | NP_343639 | YP_003534891 | sso:SSO2265 hypothetical protein K07588 LAO/AO transport system kinase [EC:2.7.-.-] (db=KEGG) (RBH) | hvo:HVO_0831 argK; ArgK-type transport ATPase (EC:2.7.-.-); K07588 LAO/AO transport system kinase [EC:2.7.-.-] (db=KEGG) (RBH) |
| Sulfolobus solfataricus P2 | Haloferax volcanii | NP_343640 | YP_003534890 | mcmA2 methylmalonyl-CoA mutase, alpha-subunit, chain B (mcmA2) (EC:5.4.99.2) K01849 methylmalonyl-CoA mutase, C-terminal domain [EC:5.4.99.2] (db=KEGG evalue=3.0e-63 bit_score=243.0 identity=100.0 coverage=99.290780141844) (BLAST) | methylmalonyl-CoA mutase , subunit B; K01849 methylmalonyl-CoA mutase, C-terminal domain [EC:5.4.99.2] (db=KEGG evalue=8.0e-74 bit_score=278.0 identity=100.0 coverage=99.2805755395683) (BLAST) |
| Sulfolobus solfataricus P2 | Haloferax volcanii | NP_343703 | YP_003534755 | sso:SSO2342 gpT-1 purine phosphoribosyltransferase (gpT-1) (EC:2.4.2.-) K07101 (db=KEGG) (RBH) | hvo:HVO_0694 gptA; purine phosphoribosyltransferase (EC:2.4.2.22); K07101 (db=KEGG) (RBH) |
| Sulfolobus solfataricus P2 | Haloferax volcanii | NP_343704 | YP_003534753 | sso:SSO2343 mtaP 5'-methylthioadenosine phosphorylase II (EC:2.4.2.28) K00772 5'-methylthioadenosine phosphorylase [EC:2.4.2.28] (db=KEGG) (RBH) | hvo:HVO_0692 mtaP; methylthioadenosine phosphorylase (EC:2.4.2.28); K00772 5'-methylthioadenosine phosphorylase [EC:2.4.2.28] (db=KEGG) (RBH) |
| Sulfolobus solfataricus P2 | Haloferax volcanii | NP_343734 | YP_003534209 | sso:SSO2373 putative RNA-processing protein K06961 (db=KEGG) (RBH) | hvo:HVO_0134 RNA-binding Pno1 homolog; K06961 (db=KEGG) (RBH) |
| Sulfolobus solfataricus P2 | Haloferax volcanii | NP_343735 | YP_003534210 | sso:SSO2374 hypothetical protein K07178 RIO kinase 1 [EC:2.7.11.1] (db=KEGG) (RBH) | hvo:HVO_0135 rio1; atypical protein kinase; K07178 RIO kinase 1 [EC:2.7.11.1] (db=KEGG) (RBH) |
| Sulfolobus solfataricus P2 | Haloferax volcanii | NP_343736 | YP_003534211 | eiF1A translation initiation factor IF-1A K03236 translation initiation factor eIF-1A (db=KEGG evalue=5.0e-56 bit_score=219.0 identity=100.0 coverage=99.0740740740741) (BLAST) | tif1A1; translation initiation factor aIF-1A; K03236 translation initiation factor eIF-1A (db=KEGG evalue=3.0e-48 bit_score=193.0 identity=100.0 coverage=98.9583333333333) (BLAST) |
| Sulfolobus solfataricus P2 | Haloferax volcanii | NP_344136 | YP_003534948 | sso:SSO2815 2-oxoacid--ferredoxin oxidoreductase, alpha chain (EC:1.2.7.-) K00174 2-oxoglutarate ferredoxin oxidoreductase subunit alpha [EC:1.2.7.3] (db=KEGG) (RBH) | hvo:HVO_0888 korA; oxoglutarate--ferredoxin oxidoreductase alpha subunit (EC:1.2.7.3); K00174 2-oxoglutarate ferredoxin oxidoreductase subunit alpha [EC:1.2.7.3] (db=KEGG) (RBH) |
| Sulfolobus solfataricus P2 | Haloferax volcanii | NP_344137 | YP_003534947 | sso:SSO2816 2-oxoglutarate ferredoxin oxidoreductase subunit beta (EC:1.2.7.-) K00175 2-oxoglutarate ferredoxin oxidoreductase subunit beta [EC:1.2.7.3] (db=KEGG) (RBH) | hvo:HVO_0887 korB; oxoglutarate--ferredoxin oxidoreductase beta subunit (EC:1.2.7.3); K00175 2-oxoglutarate ferredoxin oxidoreductase subunit beta [EC:1.2.7.3] (db=KEGG) (RBH) |
| Sulfolobus solfataricus P2 | Haloferax volcanii | NP_344168 | YP_003534597 | sso:SSO2848 ABC transporter, permease (glucose) K10197 glucose/arabinose transport system permease protein (db=KEGG) (RBH) | hvo:HVO_0531 tsgB1; ABC-type transport system permease protein (probable substrate sugar); K02025 multiple sugar transport system permease protein (db=KEGG) (RBH) |
| Sulfolobus solfataricus P2 | Haloferax volcanii | NP_344169 | YP_003534598 | sso:SSO2849 ABC transporter, permease (glucose) K10198 glucose/arabinose transport system permease protein (db=KEGG) (RBH) | hvo:HVO_0532 tsgC1; ABC-type transport system permease protein (probable substrate sugar); K02026 multiple sugar transport system permease protein (db=KEGG) (RBH) |
| Sulfolobus solfataricus P2 | Haloferax volcanii | NP_341854 | YP_003535358 | sso:SSO0307 aroC; chorismate synthase (EC:4.2.3.5); K01736 chorismate synthase [EC:4.2.3.5] (db=KEGG) (RBH) | hvo:HVO_1306 aroC; chorismate synthase (EC:4.2.3.5); K01736 chorismate synthase [EC:4.2.3.5] (db=KEGG) (RBH) |
| Sulfolobus solfataricus P2 | Haloferax volcanii | NP_341856 | YP_003535360 | sso:SSO0309 aroA; 3-phosphoshikimate 1-carboxyvinyltransferase (EC:2.5.1.19); K00800 3-phosphoshikimate 1-carboxyvinyltransferase [EC:2.5.1.19] (db=KEGG) (RBH) | hvo:HVO_1308 aroA; 3-phosphoshikimate 1-carboxyvinyltransferase (EC:2.5.1.19); K00800 3-phosphoshikimate 1-carboxyvinyltransferase [EC:2.5.1.19] (db=KEGG) (RBH) |
| Sulfolobus solfataricus P2 | Haloferax volcanii | NP_343945 | YP_003536146 | sso:SSO2615 dppF-3; peptide ABC transporter ATP-binding protein; K02032 peptide/nickel transport system ATP-binding protein (db=KEGG) (RBH) | hvo:HVO_2122 dppF4; ABC-type transport system ATP-binding protein (probable substrate dipeptides/oligopeptides); K02032 peptide/nickel transport system ATP-binding protein (db=KEGG) (RBH) |
| Escherichia coli K12 | Chlamydia trachomatis D/UW-3/CX | NP_414624 | NP_219777 | ecv:APECO1_1904 mraW; S-adenosyl-methyltransferase MraW; K03438 S-adenosyl-methyltransferase [EC:2.1.1.-] (db=KEGG) (RBH) | cta:CTA_0294 mraW S-adenosyl-methyltransferase MraW (EC:2.1.1.-) K03438 S-adenosyl-methyltransferase [EC:2.1.1.-] (db=KEGG) (RBH) |
| Escherichia coli K12 | Chlamydia trachomatis D/UW-3/CX | NP_414626 | NP_219775 | sbo:SBO_0072 ftsI; penicillin-binding protein 3; K03587 cell division protein FtsI (penicillin-binding protein 3) [EC:2.4.1.129] (db=KEGG) (RBH) | cta:CTA_0292 pbp3 penicillin-binding protein K03587 cell division protein FtsI (penicillin-binding protein 3) [EC:2.4.1.129] (db=KEGG) (RBH) |
| Escherichia coli K12 | Chlamydia trachomatis D/UW-3/CX | NP_414627 | NP_219774 | eco:b0085 murE, ECK0086, JW0083; UDP-N-acetylmuramoyl-L-alanyl-D-glutamate:meso-diaminopimelate ligase (EC:6.3.2.13); K01928 UDP-N-acetylmuramoylalanyl-D-glutamate--2,6-diaminopimelate ligase [EC:6.3.2.13] (db=KEGG) (RBH) | ctr:CT269 murE UDP-N-acetylmuramoylalanyl-D-glutamate--2,6-diaminopimelate ligase (EC:6.3.2.13) K01928 UDP-N-acetylmuramoylalanyl-D-glutamate--2,6-diaminopimelate ligase [EC:6.3.2.13] (db=KEGG) (RBH) |
| Escherichia coli K12 | Chlamydia trachomatis D/UW-3/CX | NP_414628 | NP_220275 | eco:b0086 murF, ECK0087, JW0084, mra; UDP-N-acetylmuramoyl-tripeptide:D-alanyl-D-alanine ligase (EC:6.3.2.10); K01929 UDP-N-acetylmuramoylalanyl-D-glutamyl-2,6-diaminopimelate--D-alanyl-D-alanine ligase [EC:6.3.2.10] (db=KEGG) (RBH) | ctr:CT756 murF UDP-N-acetylmuramoyl-tripeptide--D-alanyl-D-alanine ligase K01929 UDP-N-acetylmuramoylalanyl-D-glutamyl-2,6-diaminopimelate--D-alanyl-D-alanine ligase [EC:6.3.2.10] (db=KEGG) (RBH) |
| Escherichia coli K12 | Chlamydia trachomatis D/UW-3/CX | NP_414629 | NP_220276 | ecm:EcSMS35_0092 mraY; phospho-N-acetylmuramoyl-pentapeptide-transferase (EC:2.7.8.13); K01000 phospho-N-acetylmuramoyl-pentapeptide-transferase [EC:2.7.8.13] (db=KEGG) (RBH) | ctz:CTB_7621 mraY phospho-N-acetylmuramoyl-pentapeptide-transferase K01000 phospho-N-acetylmuramoyl-pentapeptide-transferase [EC:2.7.8.13] (db=KEGG) (RBH) |
| Escherichia coli K12 | Chlamydia trachomatis D/UW-3/CX | NP_414630 | NP_220277 | ecd:ECDH10B_0070 murD; UDP-N-acetylmuramoyl-L-alanyl-D-glutamate synthetase; K01925 UDP-N-acetylmuramoylalanine--D-glutamate ligase [EC:6.3.2.9] (db=KEGG) (RBH) | ctz:CTB_7631 murD UDP-N-acetylmuramoyl-L-alanyl-D-glutamate synthetase K01925 UDP-N-acetylmuramoylalanine--D-glutamate ligase [EC:6.3.2.9] (db=KEGG) (RBH) |
| Escherichia coli K12 | Chlamydia trachomatis D/UW-3/CX | NP_414632 | NP_220280 | ebw:BWG_0085 murG; undecaprenyldiphospho-muramoylpentapeptide beta-N-acetylglucosaminyltransferase; K02563 UDP-N-acetylglucosamine--N-acetylmuramyl-(pentapeptide) pyrophosphoryl-undecaprenol N-acetylglucosamine transferase [EC:2.4.1.227] (db=KEGG) (RBH) | cta:CTA_0831 murG undecaprenyldiphospho-muramoylpentapeptide beta-N-acetylglucosaminyltransferase (EC:2.4.1.227) K02563 UDP-N-acetylglucosamine--N-acetylmuramyl-(pentapeptide) pyrophosphoryl-undecaprenol N-acetylglucosamine transferase [EC:2.4.1.227] (db=KEGG) (RBH) |
| Escherichia coli K12 | Chlamydia trachomatis D/UW-3/CX | NP_414633 | NP_220281 | ebw:BWG_0086 murC; UDP-N-acetylmuramate--L-alanine ligase; K01924 UDP-N-acetylmuramate--alanine ligase [EC:6.3.2.8] (db=KEGG) (RBH) | ctr:CT762 murC, ddlA bifunctional D-alanyl-alanine synthetase A/UDP-N-acetylmuramate--L-alanine ligase K01921 D-alanine-D-alanine ligase [EC:6.3.2.4] K01924 UDP-N-acetylmuramate--alanine ligase [EC:6.3.2.8] (db=KEGG) (RBH) |
| Escherichia coli K12 | Chlamydia trachomatis D/UW-3/CX | NP_414711 | NP_220199 | efe:EFER_0191 rpsB; 30S ribosomal protein S2; K02967 small subunit ribosomal protein S2 (db=KEGG) (RBH) | ctr:CT680 rpsB 30S ribosomal protein S2 K02967 small subunit ribosomal protein S2 (db=KEGG) (RBH) |
| Escherichia coli K12 | Chlamydia trachomatis D/UW-3/CX | NP_414712 | NP_220198 | ssn:SSON_0182 tsf; elongation factor Ts; K02357 elongation factor EF-Ts (db=KEGG) (RBH) | ctr:CT679 tsf elongation factor Ts K02357 elongation factor EF-Ts (db=KEGG) (RBH) |
| Escherichia coli K12 | Chlamydia trachomatis D/UW-3/CX | NP_414713 | NP_220197 | sbc:SbBS512_E0164 pyrH; uridylate kinase (EC:2.7.4.22); K09903 uridylate kinase [EC:2.7.4.22] (db=KEGG) (RBH) | ctr:CT678 pyrH uridylate kinase K09903 uridylate kinase [EC:2.7.4.22] (db=KEGG) (RBH) |
| Escherichia coli K12 | Chlamydia trachomatis D/UW-3/CX | NP_414714 | NP_220196 | ssn:SSON_0184 frr; ribosome recycling factor; K02838 ribosome recycling factor (db=KEGG) (RBH) | ctr:CT677 frr ribosome recycling factor K02838 ribosome recycling factor (db=KEGG) (RBH) |
| Escherichia coli K12 | Chlamydia trachomatis D/UW-3/CX | NP_414719 | NP_219746 | ssn:SSON_0189 yaeT; outer membrane protein assembly factor YaeT; K07277 outer membrane protein (db=KEGG) (RBH) | ctr:CT241 yaeT OMP85 family membrane protein K07277 outer membrane protein (db=KEGG) (RBH) |
| Escherichia coli K12 | Chlamydia trachomatis D/UW-3/CX | NP_414721 | NP_219748 | sdy:SDY_0195 lpxD; UDP-3-O-[3-hydroxymyristoyl] glucosamine N-acyltransferase; K02536 UDP-3-O-[3-hydroxymyristoyl] glucosamine N-acyltransferase [EC:2.3.1.-] (db=KEGG) (RBH) | cta:CTA_0265 lpxD UDP-3-O-[3-hydroxymyristoyl] glucosamine N-acyltransferase (EC:2.3.1.-) K02536 UDP-3-O-[3-hydroxymyristoyl] glucosamine N-acyltransferase [EC:2.3.1.-] (db=KEGG) (RBH) |
| Escherichia coli K12 | Chlamydia trachomatis D/UW-3/CX | NP_414722 | NP_220047 | ssn:SSON_0192 fabZ; (3R)-hydroxymyristoyl-ACP dehydratase; K02372 3R-hydroxymyristoyl ACP dehydrase [EC:4.2.1.-] (db=KEGG) (RBH) | cta:CTA_0581 fabZ (3R)-hydroxymyristoyl-ACP dehydratase (EC:4.2.1.-) K02372 3R-hydroxymyristoyl ACP dehydrase [EC:4.2.1.-] (db=KEGG) (RBH) |
| Escherichia coli K12 | Chlamydia trachomatis D/UW-3/CX | NP_414723 | NP_220046 | ssn:SSON_0193 lpxA; UDP-N-acetylglucosamine acyltransferase (EC:2.3.1.129); K00677 UDP-N-acetylglucosamine acyltransferase [EC:2.3.1.129] (db=KEGG) (RBH) | cta:CTA_0580 lpxA UDP-N-acetylglucosamine acyltransferase (EC:2.3.1.129) K00677 UDP-N-acetylglucosamine acyltransferase [EC:2.3.1.129] (db=KEGG) (RBH) |
| Escherichia coli K12 | Chlamydia trachomatis D/UW-3/CX | NP_414948 | NP_220249 | eco:b0414 ribD, ECK0408, JW0404, ribG, ybaE; fused diaminohydroxyphosphoribosylaminopyrimidine deaminase and 5-amino-6-(5-phosphoribosylamino) uracil reductase (EC:3.5.4.26 1.1.1.193); K11752 diaminohydroxyphosphoribosylaminopyrimidine deaminase / 5-amino-6-(5-phosphoribosylamino)uracil reductase [EC:3.5.4.26 1.1.1.193] (db=KEGG) (RBH) | ctr:CT730 ribD riboflavin deaminase K11752 diaminohydroxyphosphoribosylaminopyrimidine deaminase / 5-amino-6-(5-phosphoribosylamino)uracil reductase [EC:3.5.4.26 1.1.1.193] (db=KEGG) (RBH) |
| Escherichia coli K12 | Chlamydia trachomatis D/UW-3/CX | NP_414949 | NP_220251 | sfv:SFV_0380 ribH; 6,7-dimethyl-8-ribityllumazine synthase; K00794 riboflavin synthase beta chain [EC:2.5.1.-] (db=KEGG) (RBH) | ctj:JALI_7371 ribH 6,7-dimethyl-8-ribityllumazine synthase K00794 riboflavin synthase beta chain [EC:2.5.1.-] (db=KEGG) (RBH) |
| Escherichia coli K12 | Chlamydia trachomatis D/UW-3/CX | NP_414971 | NP_220225 | ssn:SSON_0420 clpP; ATP-dependent Clp protease proteolytic subunit (EC:3.4.21.92); K01358 ATP-dependent Clp protease, protease subunit [EC:3.4.21.92] (db=KEGG) (RBH) | cta:CTA_0767 clpP ATP-dependent Clp protease proteolytic subunit (EC:3.4.21.92) K01358 ATP-dependent Clp protease, protease subunit [EC:3.4.21.92] (db=KEGG) (RBH) |
| Escherichia coli K12 | Chlamydia trachomatis D/UW-3/CX | NP_414972 | NP_220224 | ssn:SSON_0421 clpX; ATP-dependent protease ATP-binding subunit ClpX; K03544 ATP-dependent Clp protease ATP-binding subunit ClpX (db=KEGG) (RBH) | ctr:CT705 clpX ATP-dependent protease ATP-binding subunit ClpX K03544 ATP-dependent Clp protease ATP-binding subunit ClpX (db=KEGG) (RBH) |
| Escherichia coli K12 | Chlamydia trachomatis D/UW-3/CX | NP_415254 | NP_219557 | ecy:ECSE_0785 sucA; 2-oxoglutarate dehydrogenase E1 component; K00164 2-oxoglutarate dehydrogenase E1 component [EC:1.2.4.2] (db=KEGG) (RBH) | ctr:CT054 sucA 2-oxoglutarate dehydrogenase E1 component (EC:1.2.4.2) K00164 2-oxoglutarate dehydrogenase E1 component [EC:1.2.4.2] (db=KEGG) (RBH) |
| Escherichia coli K12 | Chlamydia trachomatis D/UW-3/CX | NP_415255 | NP_219558 | eck:EC55989_0710 sucB; dihydrolipoamide succinyltransferase (EC:2.3.1.61); K00658 2-oxoglutarate dehydrogenase E2 component (dihydrolipoamide succinyltransferase) [EC:2.3.1.61] (db=KEGG) (RBH) | ctl:CTLon_0306 sucB dihydrolipoamide succinyltransferase K00658 2-oxoglutarate dehydrogenase E2 component (dihydrolipoamide succinyltransferase) [EC:2.3.1.61] (db=KEGG) (RBH) |
| **Escherichia coli K12** | **Chlamydia trachomatis D/UW-3/CX** | **NP_415256** | **NP_220342** | **efe:EFER_2385 sucC; succinyl-CoA synthetase subunit beta (EC:6.2.1.5); K01903 succinyl-CoA synthetase beta subunit [EC:6.2.1.5] (db=KEGG) (RBH); B** | **ctr:CT821 sucC succinyl-CoA synthetase subunit beta (EC:6.2.1.5) K01903 succinyl-CoA synthetase beta subunit [EC:6.2.1.5] (db=KEGG) (RBH)** |
| **Escherichia coli K12** | **Chlamydia trachomatis D/UW-3/CX** | **NP_415257** | **NP_220343** | **eci:UTI89_C0724 sucD; succinyl-CoA synthetase subunit alpha (EC:6.2.1.5); K01902 succinyl-CoA synthetase alpha subunit [EC:6.2.1.5] (db=KEGG) (RBH)** | **cta:CTA_0896 sucD succinyl-CoA synthetase subunit alpha (EC:6.2.1.5) K01902 succinyl-CoA synthetase alpha subunit [EC:6.2.1.5] (db=KEGG) (RBH)** |
| Escherichia coli K12 | Chlamydia trachomatis D/UW-3/CX | NP_415609 | NP_219744 | ssn:SSON_1111 fabH; 3-oxoacyl-(acyl carrier protein) synthase III (EC:2.3.1.41); K00648 3-oxoacyl-[acyl-carrier-protein] synthase III [EC:2.3.1.180] (db=KEGG) (RBH) | cta:CTA_0261 fabH 3-oxoacyl-(acyl carrier protein) synthase III (EC:2.3.1.41) K00648 3-oxoacyl-[acyl-carrier-protein] synthase III [EC:2.3.1.180] (db=KEGG) (RBH) |
| Escherichia coli K12 | Chlamydia trachomatis D/UW-3/CX | NP_415610 | NP_219743 | ecw:EcE24377A_1213 fabD; malonyl CoA-acyl carrier protein transacylase (EC:2.3.1.39); K00645 [acyl-carrier-protein] S-malonyltransferase [EC:2.3.1.39] (db=KEGG) (RBH) | ctj:JALI_2331 fabD malonyl-CoA-[acyl-carrier-protein] transacylase K00645 [acyl-carrier-protein] S-malonyltransferase [EC:2.3.1.39] (db=KEGG) (RBH) |
| Escherichia coli K12 | Chlamydia trachomatis D/UW-3/CX | NP_415611 | NP_219742 | sbc:SbBS512_E2231 fabG; 3-ketoacyl-(acyl-carrier-protein) reductase (EC:1.1.1.100); K00059 3-oxoacyl-[acyl-carrier protein] reductase [EC:1.1.1.100] (db=KEGG) (RBH) | ctr:CT237 fabG 3-ketoacyl-(acyl-carrier-protein) reductase (EC:1.1.1.100) K00059 3-oxoacyl-[acyl-carrier protein] reductase [EC:1.1.1.100] (db=KEGG) (RBH) |
| Escherichia coli K12 | Chlamydia trachomatis D/UW-3/CX | NP_415759 | NP_219702 | eco:b1243 oppA, ECK1237, JW1235; oligopeptide transporter subunit; K02035 peptide/nickel transport system substrate-binding protein (db=KEGG) (RBH) | ctr:CT198 oppA_3 oligopeptide binding protein K02035 peptide/nickel transport system substrate-binding protein (db=KEGG) (RBH) |
| Escherichia coli K12 | Chlamydia trachomatis D/UW-3/CX | NP_415760 | NP_219703 | sbo:SBO_1823 oppB; oligopeptide transporter permease; K02033 peptide/nickel transport system permease protein (db=KEGG) (RBH) | ctr:CT199 oppB_1 oligopeptide permease K02033 peptide/nickel transport system permease protein (db=KEGG) (RBH) |
| **Escherichia coli K12** | **Chlamydia trachomatis D/UW-3/CX** | **NP_415761** | **NP_219704** | **eoj:ECO26_1756 oppC; oligopeptide transporter subunit OppC; K02034 peptide/nickel transport system permease protein (db=KEGG) (RBH)** | **ctz:CTB_1941 oppC oligopeptide transport system membrane permease K02034 peptide/nickel transport system permease protein (db=KEGG) (RBH)** |
| Escherichia coli K12 | Chlamydia trachomatis D/UW-3/CX | NP_415776 | NP_219674 | sfl:SF1263 trpA; tryptophan synthase subunit alpha (EC:4.2.1.20); K01695 tryptophan synthase alpha chain [EC:4.2.1.20] (db=KEGG) (RBH) | ctr:CT171 trpA tryptophan synthase subunit alpha K01695 tryptophan synthase alpha chain [EC:4.2.1.20] (db=KEGG) (RBH) |
| Escherichia coli K12 | Chlamydia trachomatis D/UW-3/CX | NP_415777 | NP_219673 | sfl:SF1264 trpB; tryptophan synthase subunit beta (EC:4.2.1.20); K01696 tryptophan synthase beta chain [EC:4.2.1.20] (db=KEGG) (RBH) | ctr:CT170 trpB tryptophan synthase subunit beta (EC:4.2.1.20) K01696 tryptophan synthase beta chain [EC:4.2.1.20] (db=KEGG) (RBH) |
| Escherichia coli K12 | Chlamydia trachomatis D/UW-3/CX | NP_416195 | NP_220206 | eco:b1680 sufS, csdB, ECK1676, JW1670, ynhB; cysteine desulfurase, stimulated by SufE; selenocysteine lyase, PLP-dependent (EC:4.4.1.16); K11717 cysteine desulfurase / selenocysteine lyase [EC:2.8.1.7 4.4.1.16] (db=KEGG) (RBH) | ctr:CT687 yfhO_1 cysteine desulfurase K11717 cysteine desulfurase / selenocysteine lyase [EC:2.8.1.7 4.4.1.16] (db=KEGG) (RBH) |
| Escherichia coli K12 | Chlamydia trachomatis D/UW-3/CX | NP_416197 | NP_220204 | eco:b1682 sufC, ECK1678, JW1672, ynhD; component of SufBCD complex, ATP-binding component of ABC superfamily; K09013 Fe-S cluster assembly ATP-binding protein (db=KEGG) (RBH) | cta:CTA_0746 ABC transporter ATP-binding protein K09013 Fe-S cluster assembly ATP-binding protein (db=KEGG) (RBH) |
| **Escherichia coli K12** | **Chlamydia trachomatis D/UW-3/CX** | **NP_416229** | **NP_220357** | **ecr:ECIAI1_1770 pheS; phenylalanyl-tRNA synthetase subunit alpha (EC:6.1.1.20); K01889 phenylalanyl-tRNA synthetase alpha chain [EC:6.1.1.20] (db=KEGG) (RBH)** | **ctr:CT836 pheS phenylalanyl-tRNA synthetase subunit alpha (EC:6.1.1.20) K01889 phenylalanyl-tRNA synthetase alpha chain [EC:6.1.1.20] (db=KEGG) (RBH)** |
| Escherichia coli K12 | Chlamydia trachomatis D/UW-3/CX | NP_416231 | NP_220356 | ribosomal protein L20; K02887 large subunit ribosomal protein L20 (db=KEGG evalue=8.0e-50 bit_score=198.0 identity=99.15 coverage=99.1525423728814) (BLAST) | rplT 50S ribosomal protein L20 K02887 large subunit ribosomal protein L20 (db=KEGG evalue=5.0e-65 bit_score=249.0 identity=100.0 coverage=99.1869918699187) (BLAST) |
| Escherichia coli K12 | Chlamydia trachomatis D/UW-3/CX | NP_416233 | NP_220354 | eoi:ECO111_2227 infC; translation initiation factor IF-3; K02520 translation initiation factor IF-3 (db=KEGG) (RBH) | ctr:CT833 infC translation initiation factor IF-3 K02520 translation initiation factor IF-3 (db=KEGG) (RBH) |
| Escherichia coli K12 | Chlamydia trachomatis D/UW-3/CX | NP_416375 | NP_220016 | ecx:EcHS_A1954 ruvA; Holliday junction DNA helicase RuvA; K03550 holliday junction DNA helicase RuvA (db=KEGG) (RBH) | ctr:CT501 ruvA Holliday junction DNA helicase RuvA K03550 holliday junction DNA helicase RuvA (db=KEGG) (RBH) |
| Escherichia coli K12 | Chlamydia trachomatis D/UW-3/CX | NP_416377 | NP_220017 | sbc:SbBS512_E1109 ruvC; Holliday junction resolvase (EC:3.1.22.4); K01159 crossover junction endodeoxyribonuclease RuvC [EC:3.1.22.4] (db=KEGG) (RBH) | ctb:CTL0764 ruvC Holliday junction resolvase K01159 crossover junction endodeoxyribonuclease RuvC [EC:3.1.22.4] (db=KEGG) (RBH) |
| Escherichia coli K12 | Chlamydia trachomatis D/UW-3/CX | NP_416737 | NP_220348 | ecj:JW2228 nrdA; ribonucleoside diphosphate reductase 1, alpha subunit; K00525 ribonucleoside-diphosphate reductase alpha chain [EC:1.17.4.1] (db=KEGG) (RBH) | ctr:CT827 nrdA ribonucleotide-diphosphate reductase subunit alpha (EC:1.17.4.1) K00525 ribonucleoside-diphosphate reductase alpha chain [EC:1.17.4.1] (db=KEGG) (RBH) |
| Escherichia coli K12 | Chlamydia trachomatis D/UW-3/CX | NP_416738 | NP_220349 | sfv:SFV_2308 nrdB; ribonucleotide-diphosphate reductase subunit beta (EC:1.17.4.1); K00526 ribonucleoside-diphosphate reductase beta chain [EC:1.17.4.1] (db=KEGG) (RBH) | ctr:CT828 nrdB ribonucleotide-diphosphate reductase subunit beta (EC:1.17.4.1) K00526 ribonucleoside-diphosphate reductase beta chain [EC:1.17.4.1] (db=KEGG) (RBH) |
| Escherichia coli K12 | Chlamydia trachomatis D/UW-3/CX | NP_417097 | NP_219530 | rplS; 50S ribosomal protein L19; K02884 large subunit ribosomal protein L19 (db=KEGG evalue=4.0e-60 bit_score=232.0 identity=100.0 coverage=99.1304347826087) (BLAST) | rplS 50S ribosomal protein L19 K02884 large subunit ribosomal protein L19 (db=KEGG evalue=4.0e-63 bit_score=243.0 identity=100.0 coverage=99.1735537190083) (BLAST) |
| Escherichia coli K12 | Chlamydia trachomatis D/UW-3/CX | NP_417098 | NP_219529 | sfl:SF2667 trmD; tRNA (guanine-N(1)-)-methyltransferase (EC:2.1.1.31); K00554 tRNA (guanine-N1-)-methyltransferase [EC:2.1.1.31] (db=KEGG) (RBH) | ctr:CT027 trmD tRNA (guanine-N(1)-)-methyltransferase/unknown domain fusion protein (EC:2.1.1.31) K00554 tRNA (guanine-N1-)-methyltransferase [EC:2.1.1.31] (db=KEGG) (RBH) |
| Escherichia coli K12 | Chlamydia trachomatis D/UW-3/CX | NP_417101 | NP_219527 | sbo:SBO_2746 ffh; signal recognition particle protein; K03106 signal recognition particle subunit SRP54 (db=KEGG) (RBH) | ctr:CT025 ffh signal recognition particle, subunit FFH/SRP54 K03106 signal recognition particle subunit SRP54 (db=KEGG) (RBH) |
| **Escherichia coli K12** | **Chlamydia trachomatis D/UW-3/CX** | **NP_417635** | **NP_219597** | **eum:ECUMN_3648 truB; tRNA pseudouridine synthase B (EC:4.2.1.70); K03177 tRNA pseudouridine synthase B [EC:5.4.99.12] (db=KEGG) (RBH)** | **ctr:CT094 truB tRNA pseudouridine synthase B K03177 tRNA pseudouridine synthase B [EC:5.4.99.12] (db=KEGG) (RBH)** |
| Escherichia coli K12 | Chlamydia trachomatis D/UW-3/CX | NP_417637 | NP_219599 | ecz:ECS88_3552 infB; translation initiation factor IF-2; K02519 translation initiation factor IF-2 (db=KEGG) (RBH) | ctr:CT096 infB translation initiation factor IF-2 K02519 translation initiation factor IF-2 (db=KEGG) (RBH) |
| **Escherichia coli K12** | **Chlamydia trachomatis D/UW-3/CX** | **NP_417638** | **NP_219600** | **ecz:ECS88_3553 nusA; transcription elongation factor NusA; K02600 N utilization substance protein A (db=KEGG) (RBH)** | **cta:CTA_0103 nusA transcription elongation factor NusA K02600 N utilization substance protein A (db=KEGG) (RBH)** |
| Escherichia coli K12 | Chlamydia trachomatis D/UW-3/CX | NP_417650 | NP_219928 | ecd:ECDH10B_3357 obgE; GTPase ObgE; K03979 GTP-binding protein (db=KEGG) (RBH) | ctr:CT418 obgE, cgtA, obg, yhbZ GTPase ObgE K03979 GTP-binding protein (db=KEGG) (RBH) |
| Escherichia coli K12 | Chlamydia trachomatis D/UW-3/CX | NP_417652 | NP_219929 | rpmA; 50S ribosomal protein L27; K02899 large subunit ribosomal protein L27 (db=KEGG evalue=6.0e-41 bit_score=169.0 identity=100.0 coverage=98.8235294117647) (BLAST) | rpmA 50S ribosomal protein L27 K02899 large subunit ribosomal protein L27 (db=KEGG evalue=2.0e-40 bit_score=167.0 identity=100.0 coverage=98.7951807228916) (BLAST) |
| Escherichia coli K12 | Chlamydia trachomatis D/UW-3/CX | NP_417653 | NP_219930 | rplU; 50S ribosomal protein L21; K02888 large subunit ribosomal protein L21 (db=KEGG evalue=4.0e-52 bit_score=206.0 identity=99.03 coverage=99.0291262135922) (BLAST) | rplU 50S ribosomal protein L21 K02888 large subunit ribosomal protein L21 (db=KEGG evalue=2.0e-46 bit_score=187.0 identity=100.0 coverage=99.0654205607477) (BLAST) |
| **Escherichia coli K12** | **Chlamydia trachomatis D/UW-3/CX** | **NP_417697** | **NP_219629** | **rpsI; 30S ribosomal protein S9; K02996 small subunit ribosomal protein S9 (db=KEGG evalue=9.0e-69 bit_score=261.0 identity=100.0 coverage=99.2307692307692) (BLAST)** | **rpsI 30S ribosomal protein S9 K02996 small subunit ribosomal protein S9 (db=KEGG evalue=2.0e-57 bit_score=224.0 identity=100.0 coverage=99.2248062015504) (BLAST)** |
| **Escherichia coli K12** | **Chlamydia trachomatis D/UW-3/CX** | **NP_417698** | **NP_219628** | **rplM; 50S ribosomal protein L13; K02871 large subunit ribosomal protein L13 (db=KEGG evalue=5.0e-78 bit_score=292.0 identity=100.0 coverage=99.2957746478873) (BLAST)** | **ctz:CTB_1241 rplM 50S ribosomal protein L13 K02871 large subunit ribosomal protein L13 (db=KEGG) (RBH)** |
| Escherichia coli K12 | Chlamydia trachomatis D/UW-3/CX | NP_417721 | NP_219626 | accB; acetyl-CoA carboxylase biotin carboxyl carrier protein subunit (EC:6.4.1.2); K02160 acetyl-CoA carboxylase biotin carboxyl carrier protein (db=KEGG evalue=1.0e-53 bit_score=211.0 identity=100.0 coverage=99.3589743589744) (BLAST) | ctb:CTL0378 accB acetyl-CoA carboxylase biotin carboxyl carrier protein subunit K02160 acetyl-CoA carboxylase biotin carboxyl carrier protein (db=KEGG) (RBH) |
| Escherichia coli K12 | Chlamydia trachomatis D/UW-3/CX | NP_417722 | NP_219627 | ecp:ECP_3341 biotin carboxylase C-terminal domain; region: Biotin_carb_C; cl08365 (EC:6.4.1.2); K01961 acetyl-CoA carboxylase, biotin carboxylase subunit [EC:6.4.1.2 6.3.4.14] (db=KEGG) (RBH) | ctr:CT124 accC acetyl-CoA carboxylase biotin carboxylase subunit (EC:6.4.1.2) K01961 acetyl-CoA carboxylase, biotin carboxylase subunit [EC:6.4.1.2 6.3.4.14] (db=KEGG) (RBH) |
| Escherichia coli K12 | Chlamydia trachomatis D/UW-3/CX | NP_417753 | NP_220021 | rplQ; 50S ribosomal protein L17; K02879 large subunit ribosomal protein L17 (db=KEGG evalue=6.0e-68 bit_score=258.0 identity=100.0 coverage=99.2125984251969) (BLAST) | rplQ 50S ribosomal protein L17 K02879 large subunit ribosomal protein L17 (db=KEGG evalue=1.0e-76 bit_score=287.0 identity=100.0 coverage=99.290780141844) (BLAST) |
| **Escherichia coli K12** | **Chlamydia trachomatis D/UW-3/CX** | **NP_417754** | **NP_220022** | **stt:t4090 rpoA; DNA-directed RNA polymerase subunit alpha (EC:2.7.7.6); K03040 DNA-directed RNA polymerase subunit alpha [EC:2.7.7.6] (db=KEGG) (RBH)** | **ctr:CT507 rpoA DNA-directed RNA polymerase subunit alpha (EC:2.7.7.6) K03040 DNA-directed RNA polymerase subunit alpha [EC:2.7.7.6] (db=KEGG) (RBH)** |
| **Escherichia coli K12** | **Chlamydia trachomatis D/UW-3/CX** | **NP_417756** | **NP_220023** | **rpsK; 30S ribosomal protein S11; K02948 small subunit ribosomal protein S11 (db=KEGG evalue=3.0e-69 bit_score=263.0 identity=100.0 coverage=99.2248062015504) (BLAST)** | **rpsK 30S ribosomal protein S11 K02948 small subunit ribosomal protein S11 (db=KEGG evalue=3.0e-55 bit_score=216.0 identity=100.0 coverage=99.2424242424242) (BLAST)** |
| **Escherichia coli K12** | **Chlamydia trachomatis D/UW-3/CX** | **NP_417757** | **NP_220024** | **rpsM; 30S ribosomal protein S13; K02952 small subunit ribosomal protein S13 (db=KEGG evalue=3.0e-61 bit_score=236.0 identity=100.0 coverage=99.1525423728814) (BLAST)** | **rpsM 30S ribosomal protein S13 K02952 small subunit ribosomal protein S13 (db=KEGG evalue=1.0e-54 bit_score=214.0 identity=100.0 coverage=86.8852459016393) (BLAST)** |
| **Escherichia coli K12** | **Chlamydia trachomatis D/UW-3/CX** | **NP_417759** | **NP_220025** | **sbc:SbBS512_E3685 secY; preprotein translocase subunit SecY; K03076 preprotein translocase subunit SecY (db=KEGG) (RBH)** | **ctr:CT510 secY preprotein translocase subunit SecY K03076 preprotein translocase subunit SecY (db=KEGG) (RBH)** |
| **Escherichia coli K12** | **Chlamydia trachomatis D/UW-3/CX** | **NP_417762** | **NP_220027** | **sfv:SFV_3323 rpsE; 30S ribosomal protein S5; K02988 small subunit ribosomal protein S5 (db=KEGG) (RBH)** | **ctb:CTL0774 rpsE 30S ribosomal protein S5 K02988 small subunit ribosomal protein S5 (db=KEGG) (RBH)** |
| **Escherichia coli K12** | **Chlamydia trachomatis D/UW-3/CX** | **NP_417764** | **NP_220029** | **sfl:SF3337 rplF; 50S ribosomal protein L6; K02933 large subunit ribosomal protein L6 (db=KEGG) (RBH)** | **ctz:CTB_5171 rplF 50S ribosomal protein L6 K02933 large subunit ribosomal protein L6 (db=KEGG) (RBH)** |
| **Escherichia coli K12** | **Chlamydia trachomatis D/UW-3/CX** | **NP_417765** | **NP_220030** | **rpsH; 30S ribosomal protein S8; K02994 small subunit ribosomal protein S8 (db=KEGG evalue=2.0e-69 bit_score=263.0 identity=100.0 coverage=99.2307692307692) (BLAST)** | **rpsH 30S ribosomal protein S8 K02994 small subunit ribosomal protein S8 (db=KEGG evalue=5.0e-72 bit_score=272.0 identity=100.0 coverage=99.2481203007519) (BLAST)** |
| **Escherichia coli K12** | **Chlamydia trachomatis D/UW-3/CX** | **NP_417767** | **NP_220031** | **enc:ECL_04685 50S ribosomal protein L5; K02931 large subunit ribosomal protein L5 (db=KEGG) (RBH)** | **ctj:JALI_5191 rplE 50S ribosomal protein L5 K02931 large subunit ribosomal protein L5 (db=KEGG) (RBH)** |
| **Escherichia coli K12** | **Chlamydia trachomatis D/UW-3/CX** | **NP_417769** | **NP_220033** | **rplN; 50S ribosomal protein L14; K02874 large subunit ribosomal protein L14 (db=KEGG evalue=2.0e-64 bit_score=247.0 identity=100.0 coverage=99.1869918699187) (BLAST)** | **rplN 50S ribosomal protein L14 K02874 large subunit ribosomal protein L14 (db=KEGG evalue=6.0e-63 bit_score=242.0 identity=100.0 coverage=99.1803278688525) (BLAST)** |
| **Escherichia coli K12** | **Chlamydia trachomatis D/UW-3/CX** | **NP_417770** | **NP_220034** | **rpsQ; 30S ribosomal protein S17; K02961 small subunit ribosomal protein S17 (db=KEGG evalue=2.0e-41 bit_score=171.0 identity=100.0 coverage=98.8095238095238) (BLAST)** | **rpsQ 30S ribosomal protein S17 K02961 small subunit ribosomal protein S17 (db=KEGG evalue=1.0e-39 bit_score=164.0 identity=100.0 coverage=98.7951807228916) (BLAST)** |
| Escherichia coli K12 | Chlamydia trachomatis D/UW-3/CX | NP_417772 | NP_220036 | rplP; 50S ribosomal protein L16; K02878 large subunit ribosomal protein L16 (db=KEGG evalue=2.0e-72 bit_score=274.0 identity=100.0 coverage=99.2647058823529) (BLAST) | rplP 50S ribosomal protein L16 K02878 large subunit ribosomal protein L16 (db=KEGG evalue=7.0e-74 bit_score=278.0 identity=100.0 coverage=99.2753623188406) (BLAST) |
| **Escherichia coli K12** | **Chlamydia trachomatis D/UW-3/CX** | **NP_417773** | **NP_220037** | **sfv:SFV_3334 rpsC; 30S ribosomal protein S3; K02982 small subunit ribosomal protein S3 (db=KEGG) (RBH)** | **ctr:CT522 rpsC 30S ribosomal protein S3 K02982 small subunit ribosomal protein S3 (db=KEGG) (RBH)** |
| **Escherichia coli K12** | **Chlamydia trachomatis D/UW-3/CX** | **NP_417774** | **NP_220038** | **rplV; 50S ribosomal protein L22; K02890 large subunit ribosomal protein L22 (db=KEGG evalue=4.0e-49 bit_score=196.0 identity=99.09 coverage=99.0909090909091) (BLAST)** | **rplV 50S ribosomal protein L22 K02890 large subunit ribosomal protein L22 (db=KEGG evalue=7.0e-58 bit_score=225.0 identity=100.0 coverage=99.0990990990991) (BLAST)** |
| **Escherichia coli K12** | **Chlamydia trachomatis D/UW-3/CX** | **NP_417775** | **NP_220039** | **rpsS; 30S ribosomal subunit protein S19; K02965 small subunit ribosomal protein S19 (db=KEGG evalue=4.0e-47 bit_score=189.0 identity=100.0 coverage=98.9130434782609) (BLAST)** | **rpsS 30S ribosomal protein S19 K02965 small subunit ribosomal protein S19 (db=KEGG evalue=6.0e-44 bit_score=179.0 identity=100.0 coverage=98.8636363636364) (BLAST)** |
| **Escherichia coli K12** | **Chlamydia trachomatis D/UW-3/CX** | **NP_417776** | **NP_220040** | **sfl:SF3349 rplB; 50S ribosomal protein L2; K02886 large subunit ribosomal protein L2 (db=KEGG) (RBH)** | **ctb:CTL0787 rplB 50S ribosomal protein L2 K02886 large subunit ribosomal protein L2 (db=KEGG) (RBH)** |
| Escherichia coli K12 | Chlamydia trachomatis D/UW-3/CX | NP_417778 | NP_220042 | sty:STY4359 rplD; 50S ribosomal protein L4; K02926 large subunit ribosomal protein L4 (db=KEGG) (RBH) | ctj:JALI_5301 rplD 50S ribosomal protein L4 K02926 large subunit ribosomal protein L4 (db=KEGG) (RBH) |
| **Escherichia coli K12** | **Chlamydia trachomatis D/UW-3/CX** | **NP_417779** | **NP_220043** | **sfx:S4410 rplC; 50S ribosomal protein L3; K02906 large subunit ribosomal protein L3 (db=KEGG) (RBH)** | **ctj:JALI_5311 rplC 50S ribosomal protein L3 K02906 large subunit ribosomal protein L3 (db=KEGG) (RBH)** |
| Escherichia coli K12 | Chlamydia trachomatis D/UW-3/CX | NP_417785 | NP_220086 | ecx:EcHS_A3520 gspE1; general secretory pathway protein E; K02454 general secretion pathway protein E (db=KEGG) (RBH) | ctj:JALI_5741 gspE general secretion pathway protein E K02454 general secretion pathway protein E (db=KEGG) (RBH) |
| Escherichia coli K12 | Chlamydia trachomatis D/UW-3/CX | NP_417786 | NP_220085 | ecj:JW3289 gspF; general secretory pathway component, cryptic; K02455 general secretion pathway protein F (db=KEGG) (RBH) | ctr:CT570 gspF general secretion pathway protein F K02455 general secretion pathway protein F (db=KEGG) (RBH) |
| Escherichia coli K12 | Chlamydia trachomatis D/UW-3/CX | NP_417799 | NP_219949 | sbc:SbBS512_E3714 fusA; elongation factor G; K02355 elongation factor EF-G [EC:3.6.5.3] (db=KEGG) (RBH) | ctr:CT437 fusA elongation factor G K02355 elongation factor EF-G [EC:3.6.5.3] (db=KEGG) (RBH) |
| **Escherichia coli K12** | **Chlamydia trachomatis D/UW-3/CX** | **NP_417800** | **NP_219950** | **ebw:BWG_3032 rpsG; 30S ribosomal protein S7; K02992 small subunit ribosomal protein S7 (db=KEGG) (RBH)** | **ctz:CTB_4391 rpsG 30S ribosomal protein S7 K02992 small subunit ribosomal protein S7 (db=KEGG) (RBH)** |
| Escherichia coli K12 | Chlamydia trachomatis D/UW-3/CX | NP_418155 | NP_219577 | ssn:SSON_3650 recF; recombination protein F; K03629 DNA replication and repair protein RecF (db=KEGG) (RBH) | ctr:CT074 recF recombination protein F K03629 DNA replication and repair protein RecF (db=KEGG) (RBH) |
| Escherichia coli K12 | Chlamydia trachomatis D/UW-3/CX | NP_418156 | NP_219578 | sbc:SbBS512_E4224 dnaN; DNA polymerase III subunit beta (EC:2.7.7.7); K02338 DNA polymerase III subunit beta [EC:2.7.7.7] (db=KEGG) (RBH) | ctl:CTLon_0326 dnaN DNA polymerase III subunit beta K02338 DNA polymerase III subunit beta [EC:2.7.7.7] (db=KEGG) (RBH) |
| Escherichia coli K12 | Chlamydia trachomatis D/UW-3/CX | NP_418407 | NP_219827 | sbc:SbBS512_E4468 tuf1; elongation factor Tu; K02358 elongation factor EF-Tu [EC:3.6.5.3] (db=KEGG) (RBH) | ctz:CTB_3171 tufA elongation factor Tu K02358 elongation factor EF-Tu [EC:3.6.5.3] (db=KEGG) (RBH) |
| Escherichia coli K12 | Chlamydia trachomatis D/UW-3/CX | NP_418409 | NP_219825 | sfx:S3680 nusG; transcription antitermination protein NusG; K02601 transcriptional antiterminator NusG (db=KEGG) (RBH) | ctj:JALI_3151 nusG transcription antitermination protein NusG K02601 transcriptional antiterminator NusG (db=KEGG) (RBH) |
| Escherichia coli K12 | Chlamydia trachomatis D/UW-3/CX | NP_418410 | NP_219824 | rplK; 50S ribosomal protein L11; K02867 large subunit ribosomal protein L11 (db=KEGG evalue=3.0e-68 bit_score=259.0 identity=100.0 coverage=99.2957746478873) (BLAST) | rplK 50S ribosomal protein L11 K02867 large subunit ribosomal protein L11 (db=KEGG evalue=8.0e-61 bit_score=235.0 identity=100.0 coverage=99.290780141844) (BLAST) |
| Escherichia coli K12 | Chlamydia trachomatis D/UW-3/CX | NP_418411 | NP_219823 | ssn:SSON_4157 rplA; 50S ribosomal protein L1; K02863 large subunit ribosomal protein L1 (db=KEGG) (RBH) | ctl:CTLon_0566 rplA 50S ribosomal protein L1 K02863 large subunit ribosomal protein L1 (db=KEGG) (RBH) |
| Escherichia coli K12 | Chlamydia trachomatis D/UW-3/CX | NP_418414 | NP_219820 | sfx:S3675 rpoB; DNA-directed RNA polymerase subunit beta (EC:2.7.7.6); K03043 DNA-directed RNA polymerase subunit beta [EC:2.7.7.6] (db=KEGG) (RBH) | ctr:CT315 rpoB DNA-directed RNA polymerase subunit beta (EC:2.7.7.6) K03043 DNA-directed RNA polymerase subunit beta [EC:2.7.7.6] (db=KEGG) (RBH) |
| Escherichia coli K12 | Chlamydia trachomatis D/UW-3/CX | NP_418415 | NP_219819 | sfl:SF4061 rpoC; DNA-directed RNA polymerase subunit beta' (EC:2.7.7.6); K03046 DNA-directed RNA polymerase subunit beta' [EC:2.7.7.6] (db=KEGG) (RBH) | cta:CTA_0336 rpoC DNA-directed RNA polymerase subunit beta' (EC:2.7.7.6) K03046 DNA-directed RNA polymerase subunit beta' [EC:2.7.7.6] (db=KEGG) (RBH) |
| Escherichia coli K12 | Chlamydia trachomatis D/UW-3/CX | NP_418566 | NP_219614 | groES; co-chaperonin GroES; K04078 chaperonin GroES (db=KEGG evalue=9.0e-47 bit_score=188.0 identity=100.0 coverage=98.9690721649485) (BLAST) | groES co-chaperonin GroES K04078 chaperonin GroES (db=KEGG evalue=2.0e-50 bit_score=201.0 identity=100.0 coverage=99.0196078431373) (BLAST) |
| Escherichia coli K12 | Chlamydia trachomatis D/UW-3/CX | NP_418567 | NP_219613 | sfx:S4564 groEL; chaperonin GroEL; K04077 chaperonin GroEL (db=KEGG) (RBH) | ctr:CT110 groEL chaperonin GroEL K04077 chaperonin GroEL (db=KEGG) (RBH) |
| Escherichia coli K12 | Chlamydia trachomatis D/UW-3/CX | NP_418577 | NP_220106 | sfx:S4576 frdB; fumarate reductase iron-sulfur subunit (EC:1.3.99.1); K00245 fumarate reductase iron-sulfur protein [EC:1.3.99.1] (db=KEGG) (RBH) | ctr:CT591 sdhB succinate dehydrogenase iron-sulfur subunit (EC:1.3.99.1) K00240 succinate dehydrogenase iron-sulfur protein [EC:1.3.99.1] (db=KEGG) (RBH) |
| Escherichia coli K12 | Chlamydia trachomatis D/UW-3/CX | NP_418578 | NP_220107 | sdy:SDY_4398 frdA; fumarate reductase flavoprotein subunit (EC:1.3.99.1); K00244 fumarate reductase flavoprotein subunit [EC:1.3.99.1] (db=KEGG) (RBH) | ctr:CT592 sdhA succinate dehydrogenase flavoprotein subunit (EC:1.3.5.1) K00239 succinate dehydrogenase flavoprotein subunit [EC:1.3.99.1] (db=KEGG) (RBH) |
