# Supplementary material for: A Semi-Quantitative, Synteny-Based Method to Improve Functional Predictions for Hypothetical and Poorly Annotated Bacterial and Archaeal Genes
Source: PLoS Comput Biol. 2011 Oct 20;7(10):e1002230. doi: 10.1371/journal.pcbi.1002230 (PMC3197636; doi:10.1371/journal.pcbi.1002230)
Supplement: Text S1 — Detailed explanation of methods. (DOC) [file pcbi.1002230.s012.doc]

**Sampling and genome reconstruction**

Samples for metagenomic sequencing were taken from biofilms collected from the Richmond Mine, Iron Mountain, California (40° 40’ 38.42” N and 122” 31’ 19.90” W, Elevation ~ 3,100’) in March, 2002 (5-way site) and the Ultraback A drift (UBA site) in June, 2005. A third biofilm was collected from a few meters away from the UBA site (UBA BS) in November, 2005. The 5-way site biofilm was floating on a pH 0.83 solution at 42 °C. The UBA sample was a pink biofilm partially submerged in pH 1.1, 38 °C solution, whereas the UBA BS sample was a thick, pink, gelatinous, biofilm floating on pH 1.5, 39 °C solution.

After DNA extraction, ~700 base pair-long mate-paired reads were generated by random shotgun sequencing of small insert (~3 kb) plasmid libraries (Joint Genome Institute). Each dataset, comprising ~130 Mb of sequence from the 5-way biofilm, ~110 Mb from the UBA biofilm, and ~100 Mb from the UBA BS biofilm, was assembled independently using Phred/Phrap and manually curated using Consed to resolve assembly errors. The resulting composite genome fragments (contigs) were tentatively binned based on read depth and GC content to generate well-defined genomic bins for *Leptospirillum* Group II and *Ferroplasma* Type II. Manually curated genomic datasets were generated for *Leptospirillum* Group II from the 5-way site (Simmons et al, 2008) and *Leptospirillum* Group II and III from the UBA site (Lo I, 2007; Goltsman DSA, 2009). Contigs from *Ferroplasma* Type I and Type II populations assembled largely independently, but were binned by comparison to the *Ferroplasma* *acidarmanus* isolate genome (Allen EE, 2007; Eppley JM, 2007). Remaining contigs were determined to derive from novel lineages of ARMAN Archaea (Baker BJ, 2006) and multiple populations of lower abundance *Thermoplasmatales* Archaea and Bacteria based on sampling of their 16S rRNA genes. Genome fragments of ARMAN2, a relatively abundant organism in the UBA BS dataset, were separated from other Archaea based on GC content and read depth (Comolli L, 2009). Contigs from low abundance Archaea were separated from Bacteria based on their GC content (Bacteria 52-68% vs. Archaea 34-47%) and read depth, manually curated to resolve assembly errors, and scaffolded into fragments of up to ~ 200 kb in length based on paired end sequence placement in Consed (Gordon, 2004).

**Comparative method for correlation analysis**

According to phylogenetic comparative method, genome pairs were chosen to maximize the number of pairs (Maddison WP, 2009). In order to prevent pseudo-replication, pairs were chosen from non-overlapping branches. These non-overlapping evolutionary histories preserve independence in genome rearrangements because rearrangements are not shared across separate evolutionary trajectories. It should be noted that though this method preserves phylogenetic independence, it does not necessarily find statistically independent pairs.

**Genome shearing**

The Fer1 isolate genome was sheared into pieces and included in the genome comparisons in order to test the applicability of gene and genome evolution trends to incomplete genomes. Gene calls were made on the newly sheared fragments and the synteny analysis was carried out between this organism and organisms from the NCBI genome database. In order to better represent the distribution of contig lengths found in our dataset, the number and length of fragments was chosen based on the lengths of the fragments in genomes reconstructed from all of the AMD metagenomic data. The range of fragmentation applied was from seven to 132 fragments.

**16S rRNA distance divergence measurement**

16S rRNA gene phylogeny was generated using the ARB software (Ludwig W, 2004) that calculated distance trees based on a neighbor joining method. Alignments were prepared with fast aligner in ARB and were then manually refined. A pairwise identity matrix was generated to calculate percent similarities between sequences, which are referred to as 16S rRNA distance/divergence in this study. Bootstrap values were determined using a distance-based method with default parameters.

**Gene Annotations**

Our annotation pipeline begins with gene prediction by Prodigal (ref). Protein sequences are then blasted again the KEGG database and the UniRef90 database. Reciprocal best BLAST hits with a bit score of at least 300 to a known gene in KEGG or UniRef 90. Hits to a gene containing any of the following terms were flagged as “unknowns”: hypothetical, unknown, unassigned, unclassified, undetermined, uncharacterized, putative, probable, or predicted. One-way BLAST hits with a minimum bit score of 60 are ranked next and these matches to unknown proteins were noted. Finally, we used InterproScan to identify protein family and domain hits. Rankings are assigned using the following scheme: Rank A – reserved for any gene that is manually annotated; Rank B – a gene with a reciprocal best BLAST hit to a known gene in KEGG/ UniRef90; Rank C – a gene with a BLAST hit to KEGG/UniRef90; Rank D - a gene with only InterProScan annotation; Rank E – a predicted gene with no supporting annotation (pure hypothetical).

**Operon prediction**

We assigned genes to operons based on a very conservative approach, where adjacent genes have both the same direction of transcription and are separated by no more than thirty bases of intergenic space. Synteny is commonly used in operon prediction, but we chose not to make use of this information to avoid circularity in our analysis.
